# Selective Modulation of Trk Receptors by *Cyclo*-Organopeptides

**DOI:** 10.1021/acschemneuro.4c00833

**Published:** 2025-07-09

**Authors:** Shaon Joy, Tianxiong Mi, Rui-Liang Lyu, Thitima Pewklang, Tye Thompson, Arthur Sefiani, Anyanee Kamkaew, Kevin Burgess

**Affiliations:** † Department of Chemistry, 14736 Texas A&M University, Box 30012, College Station, Texas 77842-3012, United States; ‡ School of Chemistry, Institute of Science, 506976 Suranaree University of Technology, Nakhon Ratchasima 30000, Thailand; § Department of Neuroscience and Experimental Therapeutics, Health Science Center, 12333 Texas A&M University, Bryan, Texas 77807, United States; ∥ NeuroCreis, Inc., College Station, Texas 77840, United States

**Keywords:** cyclo-organopeptides, Trk receptors, neurotrophins, loop mimics, agonist, potentiator

## Abstract

Neurotrophins (NTs, including NGF, BDNF, NT-4, and NT-3) are extracellular cytokines which modulate the survival and growth of cells expressing tropomyosin receptor kinases (Trks) A–C. Cells which express Trks include many neural tissues. For instance, corneal nerves secrete NTs to counteract epithelium disruption. Potential therapeutic applications of Trk agonism are numerous, but the use of NTs is limited by problems with production, in vivo stability, and side effects of the protein. Only humanized recombinant NGF has been clinically approved: Cenegermin for treatment of neurotrophic keratitis (NK) in the eye. Consequently, low molecular mass Trk agonists are of interest as surrogates for humanized NTs. One low molecular mass TrkA modulator from our lab, a *cyclo*-organopeptide **D3**, emerged as a clinical candidate for treatment of dry eye disease and reached phase 3 trials. However, it remains to be determined whether similar agonists or modulators of other Trks might exhibit similar effects. Moreover, **D3** was moved into trials without much optimization. This work was undertaken to identify *cyclo*-organopeptides which would activate TrkA, B, and/or C and to compare their potencies to **D3**. The immediate goal was to select compounds for studies to probe relief from desiccating stress to the eye in a mouse model relative to **D3**. In fact, in vivo assays on select compounds developed in the work described here have already been published. Three new *cyclo*-organopeptides selected for Trk agonism or modulation and **D3** were tested, and a superior lead for relief of desiccating stress in vivo was identified. Interestingly, that lead compound was designed to mimic NT-3, not NGF. This paper describes how those new *cyclo*-organopeptides were designed, prepared, and then selected via screens on Trk-transfected cells. It also outlines and explains obstacles which limit progress in this type of study.

## Graphical abstract

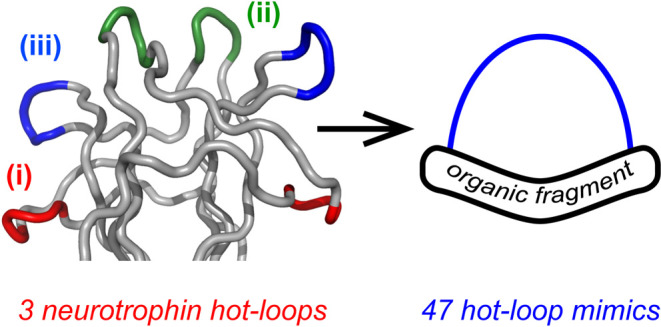

## Introduction

Tropomyosin receptor kinases (Trks) are activated by extracellular cytokines called neurotrophins (NTs, Figure 1a). This interaction initiates conformational changes in Trk intracellular regions, which activate intracellular signaling pathways including RAS-ERK, PI3K-AKT, and PLCγ-PKC, ultimately leading to cell growth, survival, and differentiation of neurons and other neuroectoderm tissues.^1^ NTs are selective for Trks (nerve growth factor, NGF for A; brain-derived neurotrophic factor, BDNF and neurotrophin-4, NT-4, for B; neurotrophin-3, NT-3 for C), but not specific.^2^ They also bind the p75 receptors which can promote apoptosis, survival, or otherwise regulate Trk activities.^3–12^ Thus, nature uses complex NT•Trk•p75 interactions to maintain and propagate neural cells.

**Figure 1:**
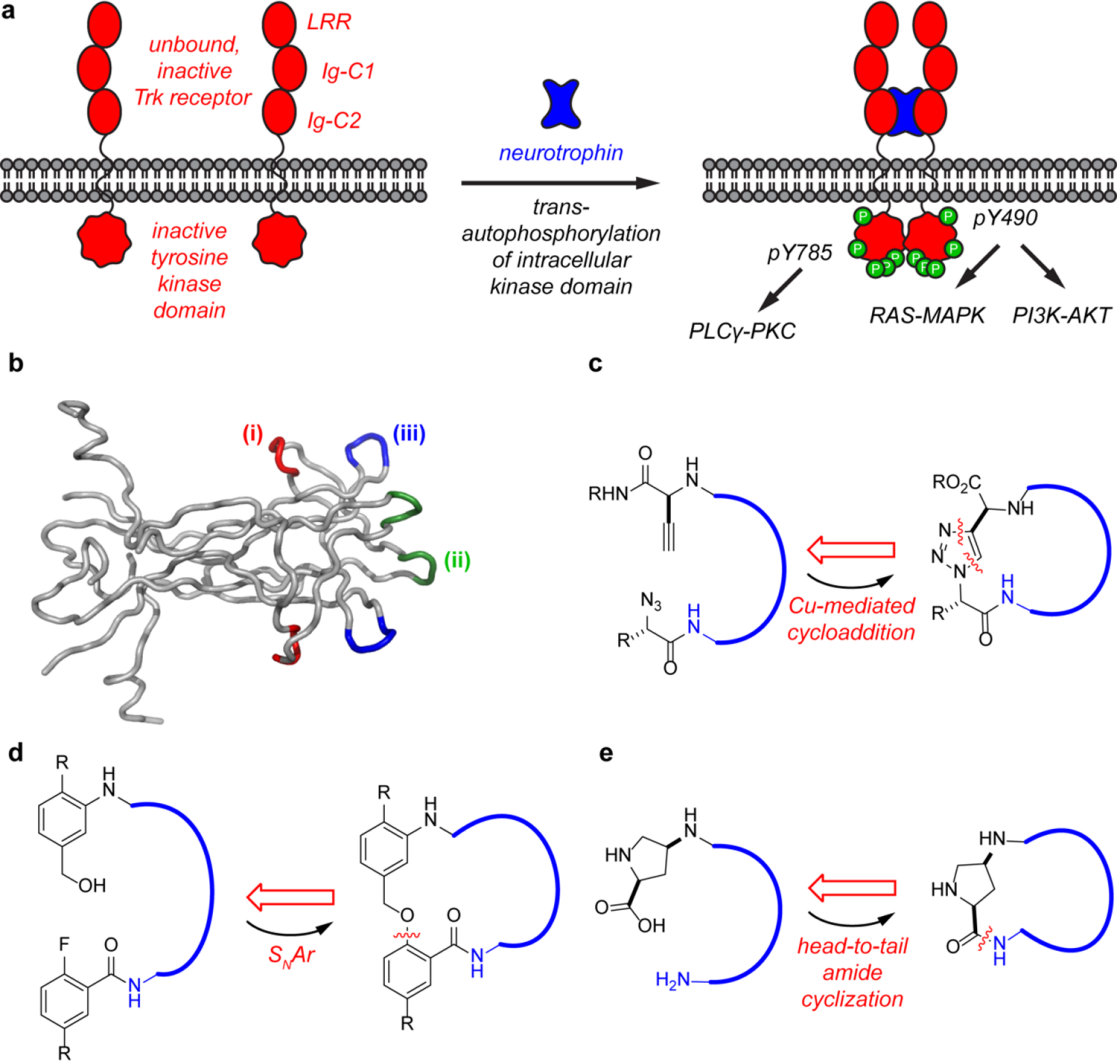
(a) Simple illustration of Trk activation by neurotrophins and subsequent intracellular signaling cascades promoting cell survival, neurite outgrowth, differentiation, and synaptic plasticity. (b) Structure of dimeric NGF and loops (i)–(iii) mimicked in this work. (c) Cu-mediated azide–alkyne cycloaddition was used to develop compound series **1**. (d) S_N_Ar reactions gave series **2**. (e) Head-to-tail peptide cyclization based on *cis*-aminoproline was used for series **3** and **4**.

Disease states influenced by NTs are numerous and include many involving neurodegeneration, trauma (including stroke), and diseases of the eye. NTs, or engineered derivatives of these, have reached clinical trials,^13,14^ but, as far as we are aware, the only one to reach the clinic is recombinant human NGF (Cenegermin) for the treatment of neurotrophic keratitis (in the eye).^15^ Clinical development of NTs is limited because their blood half-lives are on the order of minutes,^16^ they have other pharmacokinetic (PK) restrictions that disfavor them reaching the organs of interest,^16–18^ and they induce side effects.^13,14^ An alternative to NT administration is their induced endogenous expression in gene therapy strategies. However, these may induce uncontrolled neuronal growth and hence are unlikely to be approved unless they can be closely controlled. Consequently, the development of preclinical candidates from small molecule Trk agonists^13,19^ is appealing. They should have more robust PK profiles, lower costs of production, better batch-to-batch synthesis reproducibility, and more favorable shelf lives.

Strategies to identify small molecule Trk agonists can be divided into three: the first being random, or loosely guided, high throughput screening of compound libraries. Research in this area has led to several leads, some of which may be under further investigation. Approval agencies will be justifiably skeptical that compounds discovered in this way will selectively activate Trks without off-target effects because they were not designed according to any, or only tenuous, guidance from Trk or NT structural data.

The second strategy to Trk-activator design is to use peptides with sequences corresponding to NT hot segments^20^ largely responsible for Trk affinities and selectivities. Coordinates for NT•Trk interactions are available only for the Trk extracellular domains (ECDs), but they do not include the linker connecting these ECDs to the transmembrane regions. That linker participates in NT•Trk interactions, so we cannot know in detail how only the NT loop regions bind to it. Evidence from site-directed mutagenesis and chimeric NTs strongly implicates three hot loops^21–23^ in each monomer [labeled (i)–(iii) in Figure 1b] as determinants of Trk affinities and selectivities. Several cyclic peptides have been designed to mimic these hot loops. Most of those are cyclized via head-to-tail amide or Cys-to-Cys disulfides, for increased conformational rigidity and improved resistance to proteolytic degradation. However, cyclic peptides are not ideal mimics because head-to-tail cyclizations or introduction of a disulfide bond still allow significant conformational flexibility, hence unfavorable entropy losses on binding. Cyclic peptides also tend to be hydrophilic and rapidly excreted through the kidneys.

The third strategy to mimic NT hot loops was developed by our group. This involves cyclization of peptidic “warhead” fragments via endocyclic, nonpeptidic, and “organic” fragments to give *cyclo*-organopeptides. This approach was relatively successful. Prior to 1998, one of us (KB) designed and reported^24^ a *cyclo*-organopeptide **D3** (Figure 2b) which mimics one of the β-turns in NGF.^25^ **D3** is a partial agonist and NGF potentiator (synergistic activity with NGF).^26^ We used the same strategy to prepare similar NT loop mimics,^24,25,27,28^ hence generating NT-3 modulators.^29–34^ Since then, **D3** (now called Tavilermide) reached phase 3 trials for the treatment of dry eye disease and could still progress further.

**Figure 2:**
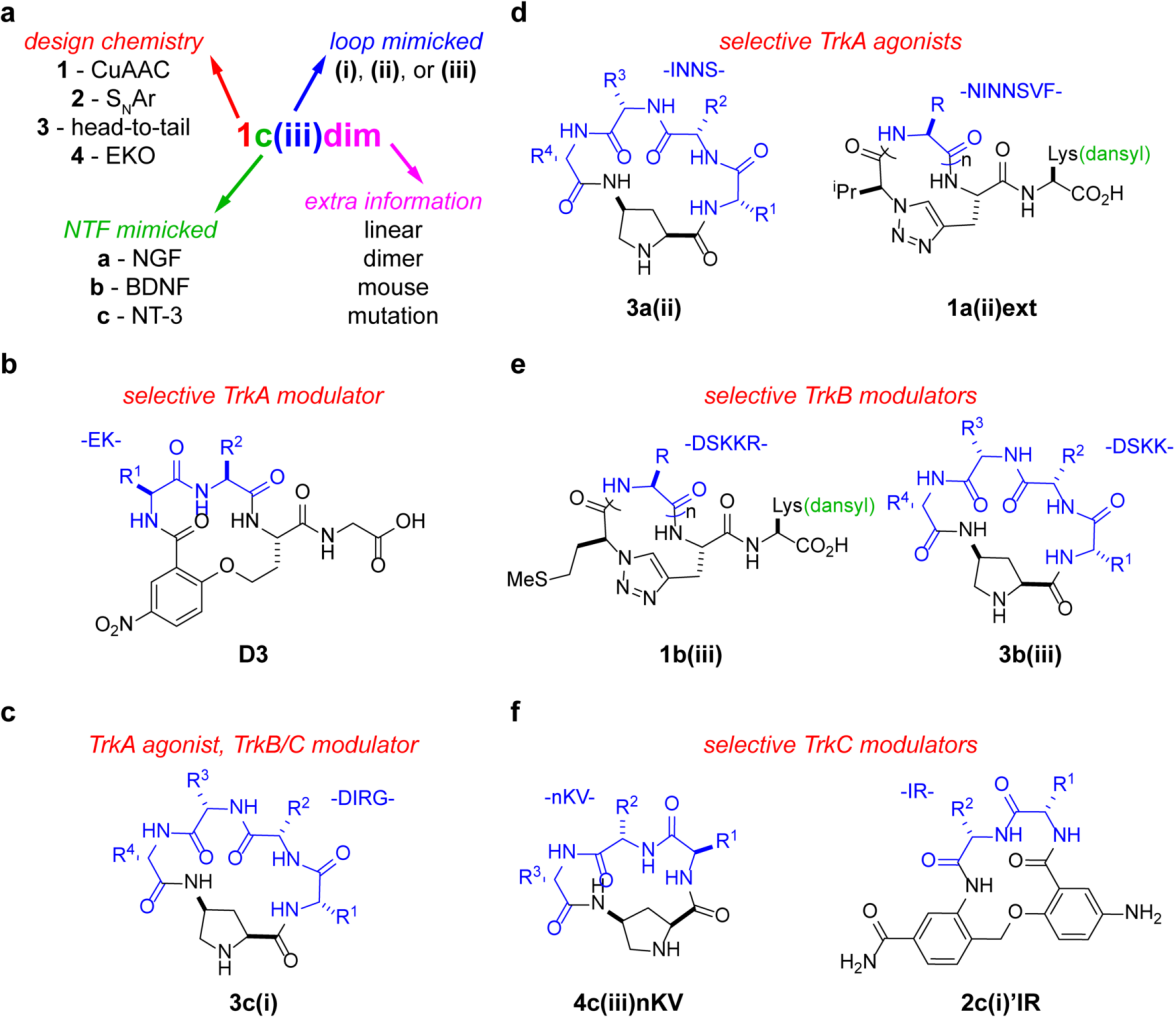
(a) Nomenclature used for the new neurotrophins (NTF) mimicked compounds. (b) The structure of previously published **D3**. (c) The structure of **3c­(i)**, best able to promote the cell survival of TrkA, B, and C-expressing cells. (d) Structures of selective TrkA agonists. (e) Structures of selective TrkB modulators. (f) Structures of selective TrkC modulators.

Our previous work primarily targeted loops (i)–(iii) of NGF, reported less on mimicry of NT-3 loops,^35^ and nothing for BDNF/NT-4. This contribution describes a broader investigation of *cyclo*-organopeptide designs based on NGF, NT-3, and BDNF warheads.

These mimics featured here were cyclized using diverse chemistries as outlined in Figure 1c–e. Forty-seven compounds of this type were prepared and tested via cell survival assays, and seven were identified for their notable effects or selectivities on cells transfected to express TrkA, B, or C. We attempted to prepare transfectants in one cell line, but this was difficult. Trk proteins were expressed but apparently did not fold properly; therefore, no tyrosine phosphorylation was observed on treatment with the neurotrophin. Consequently, we were forced to use three different Trk-transfected cell lines (HeLa, HEK293, and NIH3T3 expressing TrkA, TrkB, and TrkC, respectively). These are good options because none of those parent cell lines natively express Trk or p75.

Our objectives were to identify if any compounds within the test set were (a) Trk agonists, (b) NT potentiators (*i.e.*, compounds that modulate the Trk receptors by increasing the effects of low levels of NT), or (c) notably active and selective, or just active. Our efforts to develop secondary assays based on Trk phosphorylation were largely unsuccessful, mirroring conclusions reached by others in this field.^36,37^ Descriptions of these efforts are also outlined here, and a detailed rationale for the failure of blotting for phosphorylation is suggested.

## Results and Discussion

### Compound Design

Our numbering system is outlined in Figure 2a. Series **1**–**3** compounds were designed using only L-amino acids (*i.e.*, protein-encoded amino acids) corresponding exactly to the loops (**i**–**iii**) they were designed to mimic. Series 4 compounds are based on slightly different criteria. For these, every accessible low-energy conformation of *cis*-aminoproline-containing cyclic tetrapeptide **4** (where R1–3 = −CH_3_ with all combinations of l- and d-Ala) was simulated and then overlaid on crystal structures of the NT loops. Further, the algorithm matches sets of three amino acids Cα–Cβ on both contiguous and noncontiguous amino acids. The procedure used for this was based on the algorithm Exploring Key Orientations (EKO) designed in our group.^38^ Thus, only series **4** compounds might contain D- and noncontiguous amino acids. In total, a library of 47 compounds was prepared.

### Fixed-Dose Cell Survival Assays

Assays featuring three transfectants (for TrkA, B, and C) were used to test the original pool of 47 compounds (see Figures S2–S4). Cell viability assays were first conducted to eliminate cytotoxic compounds from the pool: none were cytotoxic in any of the cell lines tested (Figure S1). Throughout, compounds were tested at 50 μM in TrkA- and C-expressing cells and 0.4 μM in TrkB-expressing cells. NT concentrations which resulted in maximal cell survival were determined in dose–response studies, then those giving ∼25% of this value were selected for experiments featuring suboptimal NT. Samples were tested for promoting cell survival in the absence of NTs (agonism) or in the presence of suboptimal concentrations of NTs whose loops they were designed to mimic (NT potentiation). Throughout, the term agonist is reserved for compounds that induce cell survival in the absence of added NTs, partial agonist is one that has a lower activity ceiling than the native NT but still gives measurable agonism, while those only giving a significant effect in the presence of suboptimal NT are referred to as potentiators. For instance, **D3** is predominantly a potentiator, working most effectively to increase the efficacy of NGF.^35,39^

Seven loop mimics were selected for further studies (Figure 2c–f). Full data from all screens are given in Figures S2–S4, and details of fixed-dose cell survival data for the seven select compounds are in Figure 3, revealing how the compounds were selected. The top two compounds (agonists or potentiators) for each Trk, A–C, were represented. Additionally, **3c­(i)** was chosen because it was one of the most active for all three Trks, whereas the other six showed selectivities. Interestingly, **3c­(i)** and **2c­(i)’IR** are the only loop (i) mimics in the selected seven. Four amino acids are incorporated in **3c­(i) in Figure 5a**, –DIRG–, whereas **2c­(i)’IR** has –IR–; this might be part of the reason **3c­(i)**, and not **2c­(i)** activates all of the Trks, a *pan* Trk agonist or NT potentiator.

**Figure 3:**
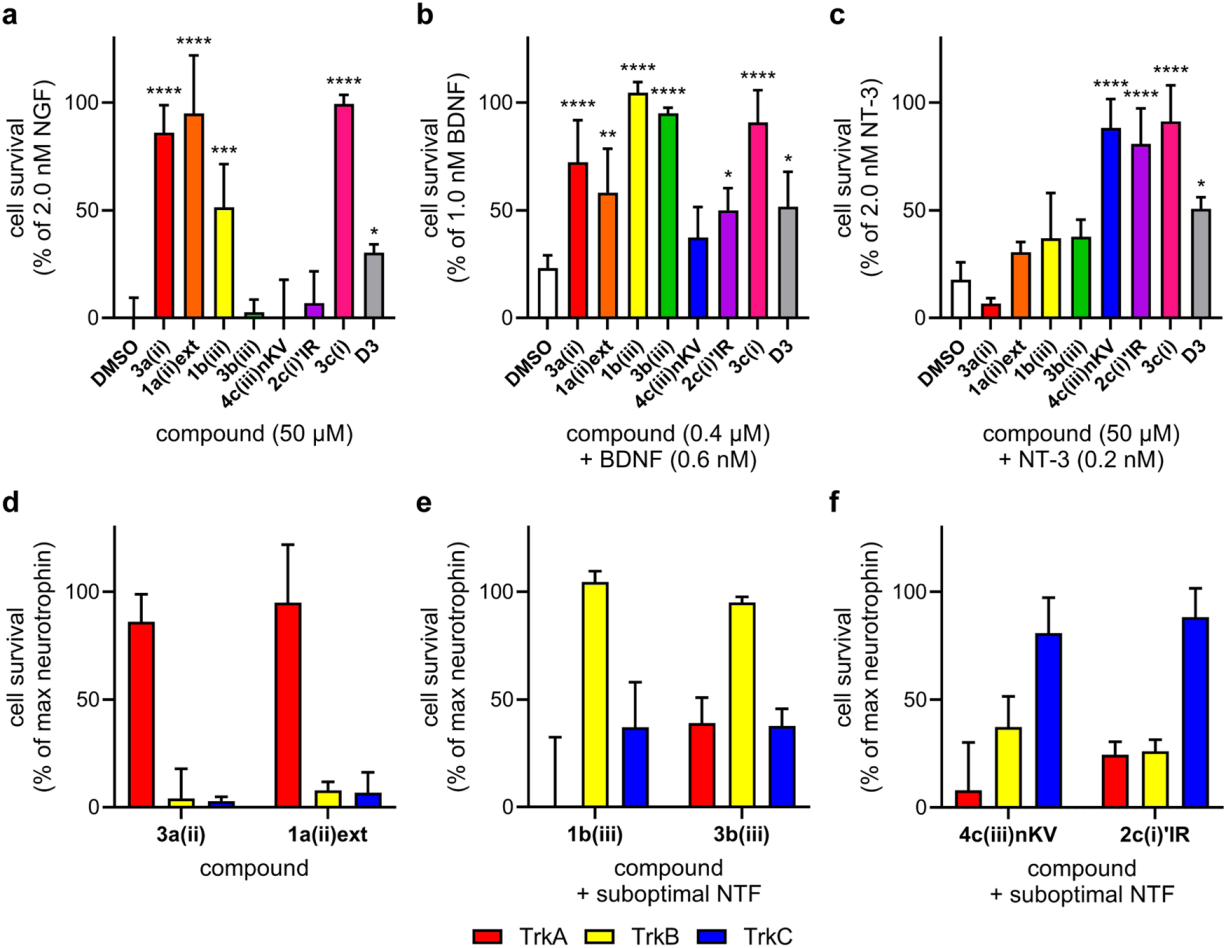
Cell survival induced by the best compounds in (a) HeLa-TrkA, (b) HEK293-TrkB, and (c) NIH3T3-TrkC cells. Selectivity of the top compounds in cell survival assays. (d) Selective TrkA agonists **3a­(ii)** and **1a­(ii)­ext**. (e) BDNF potentiators are **1b­(iii)** and **3b­(iii)**. (f) NT-3 potentiators are **4c­(iii)­nKV** and **2c­(i)’IR**. Data analyzed by one-way ANOVA followed by Dunnett’s *t* test, where *p < 0.05, **p < 0.01, ***p < 0.001, ****p < 0.0001 relative to the DMSO control.

Data for **3c­(i)** is excluded in Figure 3d–f to highlight selectivities of the other six hit compounds, which show **3a­(ii)** and **1a­(ii)­ext** are selective TrkA agonists (Figure 3d). This is important because our clinical lead **D3** is only a partial agonist, and it is primarily an NGF potentiator (data for parallel assays on **D3** are presented in Figure 3 for comparison). Hits **1b­(iii)** and **3b­(iii)** are less selective, and both of these compounds are BDNF potentiators rather than direct TrkB agonists. These observations are exciting insofar as these compounds are selective (though unfortunately not specific) mimics of BDNF or NT-3 modulation. Moreover, Figure 3c shows **2c­(i)’IR** and **4c­(iii)­nKV** selectively modulate cell survival through the TrkC receptors. The next step was to establish whether these trends are dose dependent.

### Dose-Dependent Cell Survival

Dose dependence data for the key compounds are shown in Figure 4 (recall that Figure 3 compares fixed compound doses). Figure 4a shows peptidomimetics that selectively promoted the survival of the TrkA transfectants without NGF. Mimics **3a­(ii)** and **1a­(ii)­ext** did so with a sigmoidal dose dependence, and the EC_50_ values were almost the same (10.2 and 7.1 μM, respectively). However, compounds that selectively potentiated the survival of TrkB transfectants with suboptimal neurotrophin, **1b­(iii)** and **3b­(iii)**, exhibited a bell-shaped dose–response curve (Figure 4b), so EC_50_s could not be determined. Compound solubility limits in these assays were ∼100 μM, hence it was impossible to determine whether or not all of the compounds would give bell-shaped responses if the concentration ranges were expanded. NT-3 potentiator **4c­(iii)­nKV** gave a sigmoidal dose response (EC_50_ 8.7), but the data for **2c­(i)’IR** was not well behaved (a crude EC_50_ estimate is 15.9 μM), so this is excluded from further discussion.

**Figure 4:**
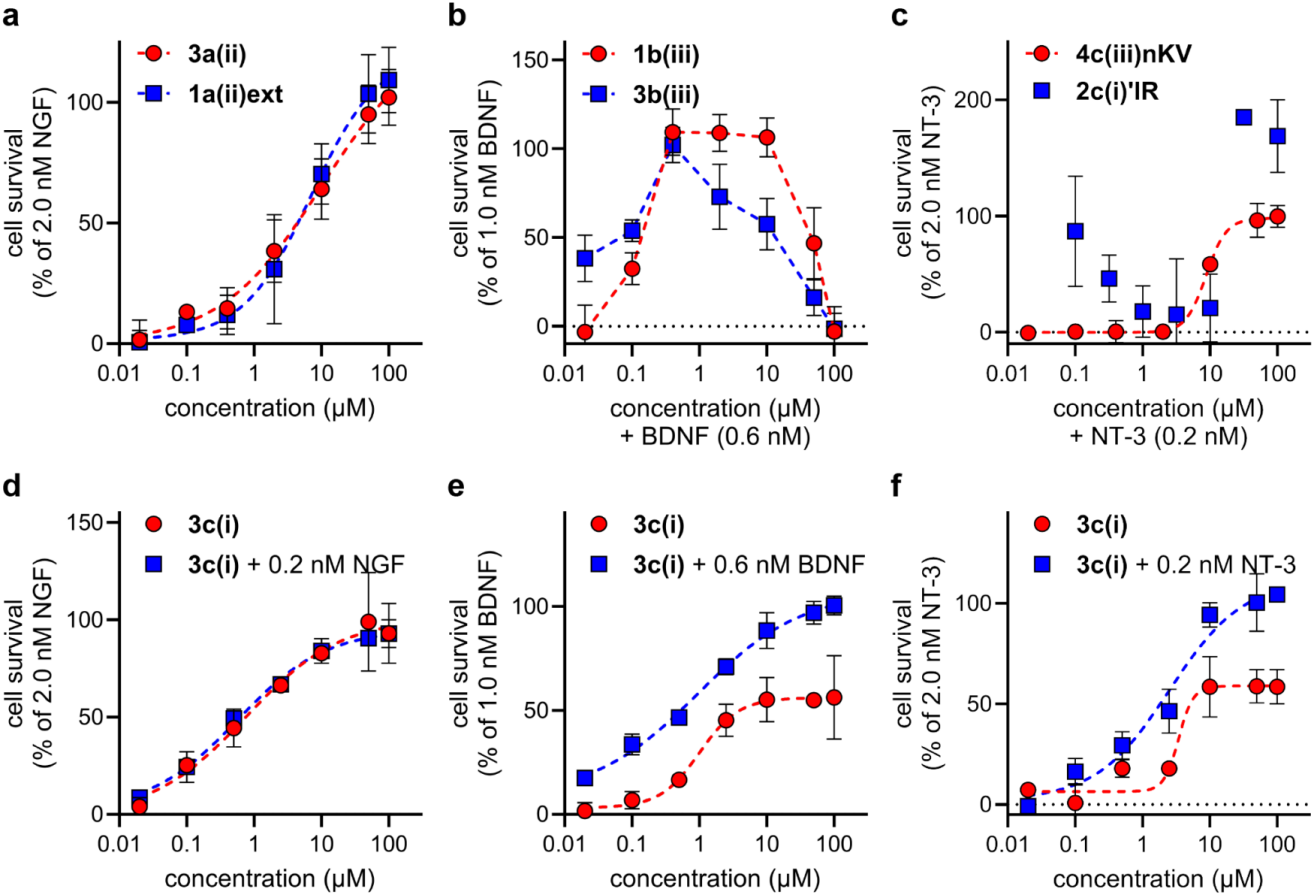
(a) **3a­(ii)** and **1a­(ii)­ext** dose response in HeLa-TrkA cells. (b) **1b­(iii)** and **3b­(iii)** dose response with 0.6 nM BDNF in HEK293-TrkB cells. (c) **4c­(iii)­nKV** and **2c­(i)’IR** dose response with 0.2 nM NT-3 in NIH3T3-TrkC cells. (d–f) Dose–response experiments with **3c­(i)** in TrkA-, B-, and C-expressing cells, respectively. Dose–response curves for (a, c–f) generated using nonlinear regression- {agonist} vs response-variable slope (four parameters) function in GraphPad Prism 10.2.

Recall **3c­(i)** is an agonist of TrkA (no NGF required) and a *potentiator* of BDNF and NT-3. Figure 4d shows the presence or absence of suboptimal NGF has no impact on tests for agonism featuring the TrkA transfectants; EC_50_s measured were similar in both cases (0.8 and 0.6 μM). Conversely, **3c­(i)** potentiated suboptimal BDNF and NT-3 without agonism (Figure 4e,f, respectively). For TrkB-expressing cells, **3c­(i)**, an EC_50_ of 1.0 μM was measured, added BDNF had no effect, and the mimic’s activity reached a maximum of ∼53% of the maximum survival imparted by BDNF (Figure 4e). In TrkC cells, **3c­(i)** gave 3.4 and 2.5 μM EC_50_ without and with suboptimal NT-3, respectively. Similar to its effects in TrkB-expressing cells, on its own, **3c­(i)** reaches a maximum activity of ∼52% survival compared with that imparted by NT-3 (Figure 4f).

Extensive efforts were made to monitor Trk activation by these compounds via conventional Western blotting (data not shown) and to observe downstream phosphorylation of AKT and MAPK via {more robust} enzyme-linked fixed-cell immuno (ELFI) assays (Figures S5 and S6). All procedures were successfully validated using the parent neurotrophin (*Z*’ > 0.5),^40^ but no significant effects were observed for the seven select compounds up to the 100 μM concentration ceiling.

### In Vivo Assays for Treatment of Dry Eye Disease

Three of the compound sets were selected for advanced murine models for the discovery of potential treatments of dry eye disease.^41^ Thus, **3a­(ii)** was selected because it promoted the cell survival of TrkA transfectants via *agonism*. Recall that the clinical candidate **D3** is a potentiator with only weak agonistic activity: this was also tested in these assays for comparison. One of the compounds which selectively potentiated suboptimal NT-3 in cell survival assays featuring TrkC cells, **4c­(iii)­nKV**, and **3c­(i)**, the *pan* activator (agonist of TrkA, and potentiator of BDNF and NT-3) was also tested. These in vivo assays are time-consuming, expensive, and involve sacrificing many mice; therefore, the number of compounds selected was limited. Other loop mimics in this study could also be active, but that remains to be determined.

All of the data for these in vivo assays is now published^42,43^ and will only be summarized here. One of the compounds showed no significant activity in the preliminary in vivo screen: **3a­(ii)**, the agonist of TrkA. This negative result is surprising because **D3** is a potentiator of NGF in TrkA transfectants. Further, **4c­(iii)­nKV** (a selective NT-3 potentiator) showed increased corneal barrier function. The *pan* loop mimic **3c­(i)** also increased corneal barrier function, consistent with agonism of the TrkA transfectants.

Overall, **4c­(iii)­nKV** was judged the most promising lead, and it was selected for further studies; it was shown to increase conjunctival goblet cell densities. Biochemical experiments featuring **4c­(iii)­nKV** indicate that it decreases NFκB activation and increases expression of some other proteins, all markers of decreased inflammation.

## Conclusions

In summary, our findings indicate that *cyclo*-organopeptides designed to resemble NT hot loops induced cell survival in stable transfectants derived from Trk- and p75-negative cell lines. All were tested against TrkA–C transfectants in the fixed-dose assays. Two designed to mimic NGF loops, **3a­(ii)** and **1a­(ii)­ext**, stood out for inducing cell survival of TrkA-expressing cells (recall that **a** in our nomenclature denotes those designed to mimic NGF loops). Two *cyclo*-organopeptides (**1b­(iii)** and **3b­(iii)**, both of loop **(iii)**) had selectivities for TrkB and were designed to be BDNF loop mimics. Similarly, **4c­(iii)­nKV** and **2c­(i)’IR** selectively promoted the survival of TrkC-expressing cells, and both were based on NT-3 loops.

Compound **3c­(i)** was, as the name describes, designed to mimic the NT-3 loop **(i)**. It is an agonist of TrkA and potentiator of BDNF and NT-3, acting through TrkB and TrkC, respectively. Figure 5 explains why this type of result is unsurprising. Lead **3c­(i)** has the peptide warhead sequence of human NT-3 loop **(i)**, and hence DIRG in Figure 5 is color-coded blue. However, loop **(i)** in the natural TrkA ligand NGF has only one residue difference, and the natural TrkB-selective ligands, BDNF and NT-4, have two and one, respectively (coded red in Figure 5). Further, several of the residue changes are conservative, e.g., Met and Leu for *iso*-Leu, and Lys for Arg.

**Figure 5:**
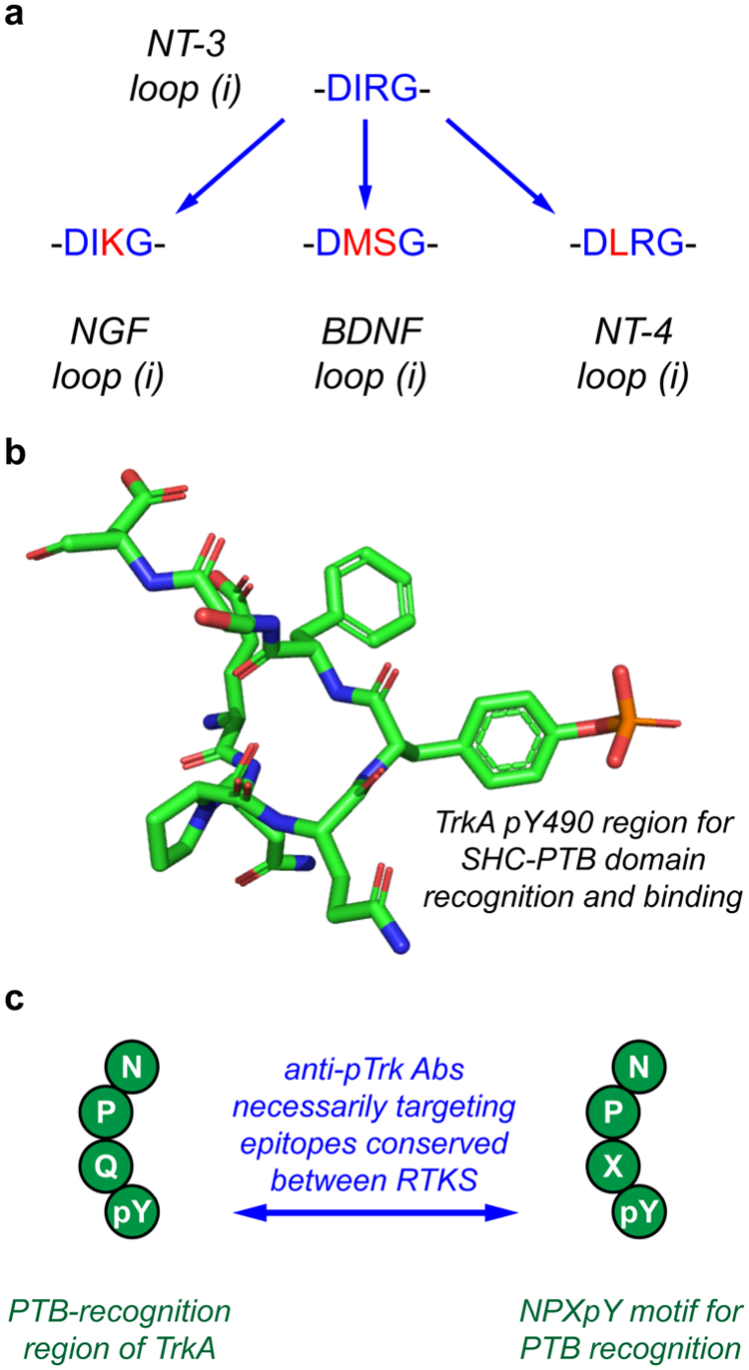
(a) Loop 1 sequence homology between NGF, BDNF, NT-3, and NT-4 could account for **3c­(i)** cross-reactivity. (b, c) TrkA amino acids surrounding pY490 residue that interact with the PTB-domain of SHC (PDB 1SHC) (b)^44^ resemble conserved epitopes recognized by PTB and SH2 domains of effector proteins (c).^45^

Hot loop sequence overlap is consistent with cross-talk of NTs binding Trks.^2^ It is reasonable that *cyclo*-organopeptides based on only 3 or 4 warhead residues affect cells transfected with Trks, they were not specifically designed to impact.

Seven compounds were selected based on potency and selectivity in cell survival assays from the initial library of 47, but others also had positive effects. Overall, we assert these findings, in conjunction with our cumulative data on loop mimics targeting TrkA and C,^29,33,35,39,41–43,46,47,48,49,50,51,52,53,54,55,56,57,58,59,60,61^ indicate *cyclo*-organopeptides are privileged structures for neurotrophin loop mimicry.

Progress in this field is limited by a lack of convenient secondary assays to bridge the gap between cell survival preliminary screens and in vivo studies. Radiolabeling is possible, but impractical for libraries of this size. Other methods, such as those based on NMR, are unreliable because they involve solubilized Trk receptors which may be incorrectly folded, aberrantly assembled into Trk oligomers, and do not necessarily contain the loop binding domains. It is a serious impediment that Western and ELFI assays do not work in this type of study.

Rationales that might explain why Western and ELFI assays cannot be routinely used to differentiate activation by NT loop mimics include the following: (i) half-lives for TrkA activation by loop mimics are considerably less (we estimate ∼10 min) than the parent NTs (∼1–2 h); (ii) their activation effects are relatively weak; (iii) observation of phosphorylated Trk in Western blotting experiments relies on pTrk antibodies, which may not be reliable; (iv) lack of sensitivity of these antibodies increases noise in blotting. Some of these assertions require further comment. Half-lives for activation via small molecules that permeate in and out of receptor environments relatively rapidly are likely to be short (i). This assertion may also account for their weak activation effects (ii). With regard to the lack of Ab sensitivity (iv), many commercial pTrk Abs are selected to detect intracellular regions homologous to other RTK receptors. Interactions of “pTrk” Abs with off-target RTKs (Figure 5c) decrease sensitivities by increasing noise. Factors (iii) and (iv) do not apply in ELFI assays, however. ELFI for Trk has higher throughput and features robust Abs, *e.g.*, pAKT and pMAPK,^36^ but apparently still does not have the sensitivity required.

Currently, we are working on loop mimics, which are cyclized using endocyclic fluorophores, giving intrinsically fluorescent loop mimics to enable certain secondary assays.^60,61^ These loop mimics binding live Trk-expressing cells and localizing in certain organelles can be observed via fluorescence-based methods.

This study was not undertaken to validate the EKO procedure mentioned in the introduction, and to do so would have required more calculations, compound syntheses, and testing. However, it is interesting that **4c­(iii)­nKV**, designed using EKO, emerged as the best candidate. Recall that design **4** is the only one that included d-amino acids (selected via EKO-guided virtual screening). Mimic **4c­(iii)­nKV** was designed on loop **(iii)** of the NT-3 sequence –ENNKLV–. Thus, **4c­(iii)­nKV** is exceptional because it comprises d-Asn, l-Lys, and l-Val, *i.e.*, nKV rather than NKV, and because these are *not* contiguous in the parent NT-3 loop. This compound was selected as one of three to be tested in vivo and proved the most promising. This may be a coincidence or it could be indicative of an extra insight given by applying the EKO algorithm.

Parenthetically, we developed another algorithm *Backbone Matching* (BM)^62^ after the data was collected for this study. BM is designed specifically for *cyclo*-organopeptides, whereas EKO^38^ matches less comprehensively on molecules designed to bear three amino acid side chains.

We suggest the development of a robust secondary assay for small molecular Trk activators and further applications of computational techniques such as EKO and BM to NT loop mimicry are the types of innovations which will drive progress in this area.

## Supplementary Material



## Notes

Synthesis and characterization data (HPLC and HRMS) for all novel compounds in this publication, procedures and results from cytotoxicity assays (Figure S1), cell survival assays (Figures S2–S4), and ELFI (Figures S5 and S6) (PDF)
