# Supplementary material for: Selective Modulation of Trk Receptors by Cyclo-Organopeptides
Source: ACS Chem Neurosci. 2025 Jul 9;16(15):2776–84. doi: 10.1021/acschemneuro.4c00833 (PMC12333011; doi:10.1021/acschemneuro.4c00833)
Supplement: Supplementary file 1 [file cn4c00833_si_001.pdf]

Supporting Information

Selective Modulation Of Trk Receptors By *Cyclo*-Organopeptides

Shaon Joy,[1] Tianxiong Mi,[1] Rui-Liang Lyu,[1] Thitima Pewklang,[1] Tye Thompson,[1] Arthur Sefiani,[3,4] Anyanee Kamkaew,[2] and Kevin Burgess*[1]

[1] *Department of Chemistry, Texas A & M University, Box 30012, College Station, TX 77842-3012, USA.*

[2] *School of Chemistry, Institute of Science, Suranaree University of Technology, Nakhon Ratchasima 30000, Thailand.*

[3] *Department of Neuroscience and Experimental Therapeutics, Texas A&M University, Health Science Center, Bryan, TX, 77807, United States.*

[4] *NeuroCreis, Inc., College Station, TX, 77840, United States.*

E-mail: burgess@tamu.edu
TITLE RUNNING HEAD: small molecule peptidomimetic

A. Synthesis and Characterization

Series 1

Compound Sequences and Structures

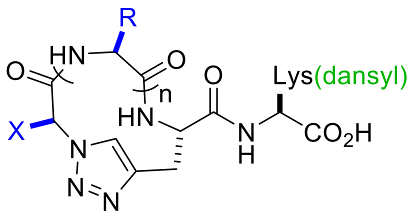

cyclic, general structure

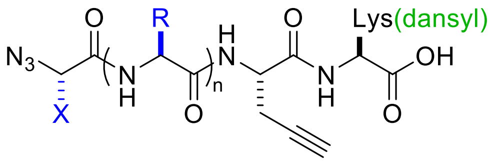

linear peptide (**lin**)

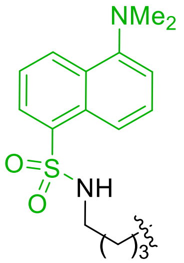

Lys(dansyl)

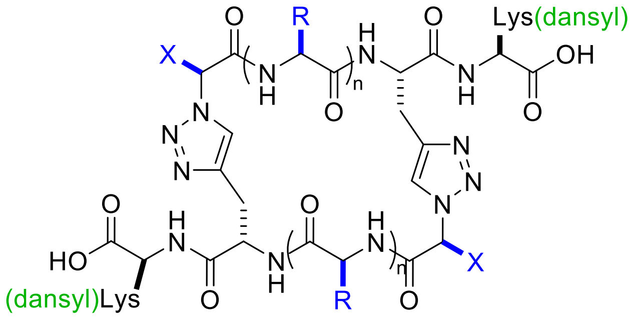

cyclic dimer (**dim**)

extra information:

dim: cyclic dimer
ext: extended sequence
lin: linear peptide
mouse: sequence from mouse NT
mut: mutated residue

name	X	R_n
**1a(i)**	T	DIKGK
**1a(ii)ext**	V	NINNSVF
**1a(iii)**	M	DGKQA
**1a(iii)mut**	M	DEKQA
**1a(iii)mouse**	T	DEKQA
**1b(i)**	V	DMSGG
**1b(ii)**	V	SKGQ
**1b(ii)lin**	V	SKGQ
**1b(iii)**	M	DSKKR
**1c(i)**	I	DIRGH
**1c(i)lin**	I	DIRGH
**1c(ii)dim**	K	TQNSP
**1c(ii)ext**	I	KTQNSPV
**1c(ii)mutlin**	K	TQNSG
**1c(ii)mutdim**	K	TQNSG
**1c(ii)mouse**	K	TGNSP
**1c(ii)mousedim**	K	TGNSP
**1c(iii)**	S	ENNKLV

General Procedure for the Synthesis of Series 1 Compounds

Fmoc-Lys(dansyl)-OH:

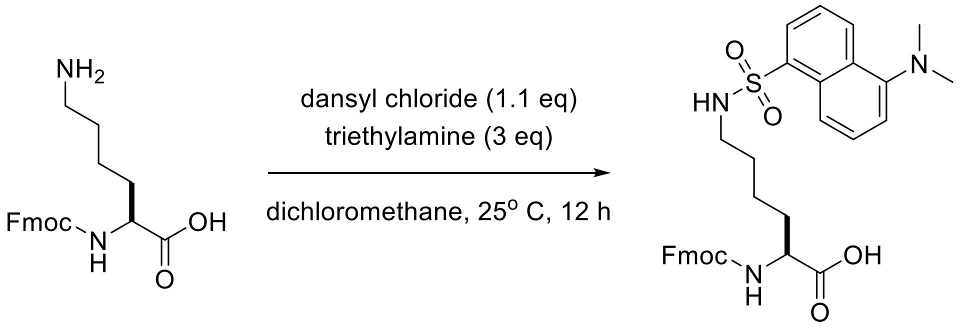

Synthesis of dansylated lysine was adapted from previously published protocols.[1] Fmoc-Lys-OH (5 mmol) was dissolved in 50 mL of dry dichloromethane under N_2. Triethylamine (3 eq, 15 mmol) followed by dansyl chloride (1.1 eq, 5.5 mmol) were added and the mixture stirred under nitrogen for 12 hours. The solution was neutralized with glacial acetic acid (3 eq, 15 mmol) followed by purification via flash column chromatography starting with 100% hexanes, gradually increasing to 1:1 hexanes:ethyl acetate.

α-Azido Acids:

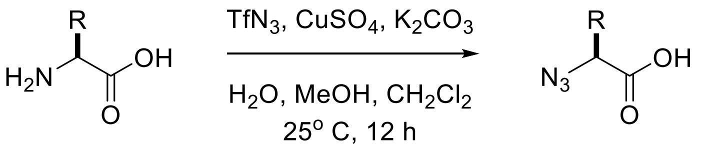

α-Azido acids were prepared as previously reported in the literature.[2] Briefly, preparation was achieved by dissolving the amino acid, $CuSO_4$, and K_2CO_3 in a 1:2 H_2O:MeOH mixture. Triflic azide in dichloromethane was added, and the mixture stirred overnight. The azido acids were purified via a buffered extraction to remove the sulfonamide byproduct.

Peptide Synthesis and Cyclization:

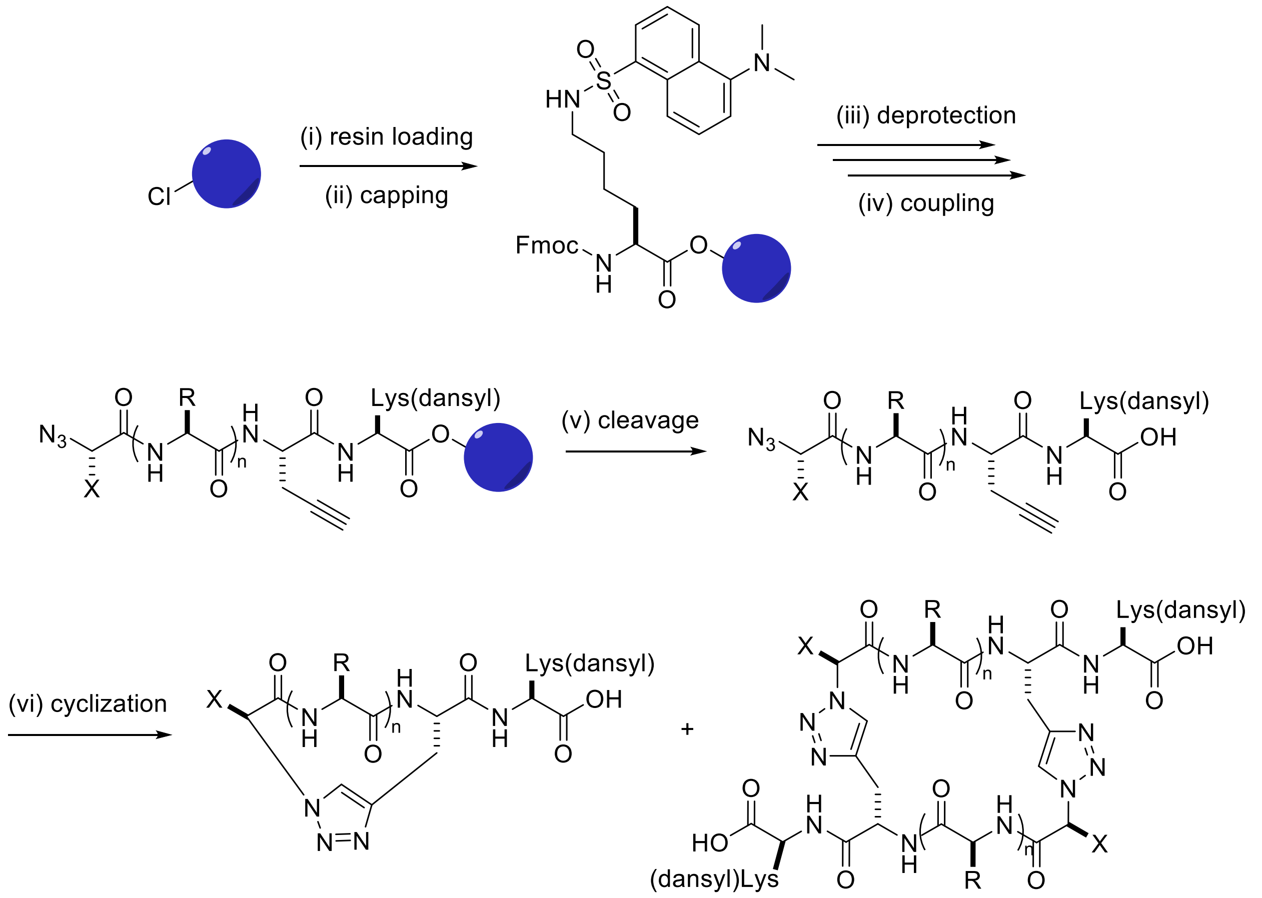

Series 1 compounds were prepared via standard Fmoc synthesis procedure on a Liberty Blue peptide synthesizer on 2-chlorotrityl resin, followed by cleavage and cyclization in solution.[3] (i) 2-Chlorotrityl resin was pre-swelled in dichloromethane (DCM), then 2.5 mL of 0.2 M Fmoc-Lys(dansyl)-OH in dimethylformamide (DMF) was added to the resin followed by 2.5 mL of 0.5 M diisopropylethylamine (DIEA) in DCM. The mixture was microwaved at 50° C for 30 minutes, washed twice with DMF, then the loading cycle repeated one more time. (ii) Free Cl groups on the resin were capped using 2 cycles of 8 mL of 1:3:7 DIEA:methanol:DCM for 10 minutes at room temperature. (iii) and (iv) The Fmoc groups were deprotected with 20% piperidine in DMF at 60° C for 4 minutes, then washed 4 times with DMF. 2.5 mL of 0.2 M Fmoc-amino acid (or amino azide X as the final coupling) in DMF, 1 mL of 0.45 M hexafluorophosphate azabenzotriazole tetramethyl uranium (HATU) in DMF, and 0.5 mL of 0.5 M DIEA in DMF were added to the reaction vessel and coupled at 50° C for 8 minutes. Coupling cycles were repeated until the desired linear peptide was complete. (v) Protected peptides were cleaved from the resin using 5% trifluoroacetic acid (TFA) in DMF and the solvent removed *in vacuo*. (vi) Peptides were cyclized at a concentration of 1 mM in DMF using copper (II) sulfate- pentahydrate (0.2 eq), sodium ascorbate (0.5 eq), and DIEA (5 eq). Argon gas was bubbled through the solution for 15 minutes and the reaction allowed to run overnight. Cyclized products were deprotected for 2 hours with a solution of 95% TFA, 2.5% H_2O, 2.5% triisopropylsilane. Solvent was removed with a stream of nitrogen, then the peptide product precipitated from cold ether and purified via preparative HPLC. Dimer products were purified and collected as side-products of the cyclization reaction and tested in addition to the cyclic monomers. Purity and identity were determined by analytical HPLC, LCMS, and HRMS.

Characterization (HPLC and HRMS) for Series 1 Compounds

1a(i)

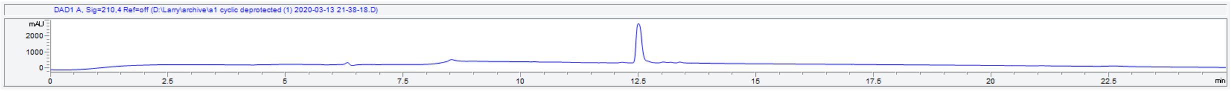

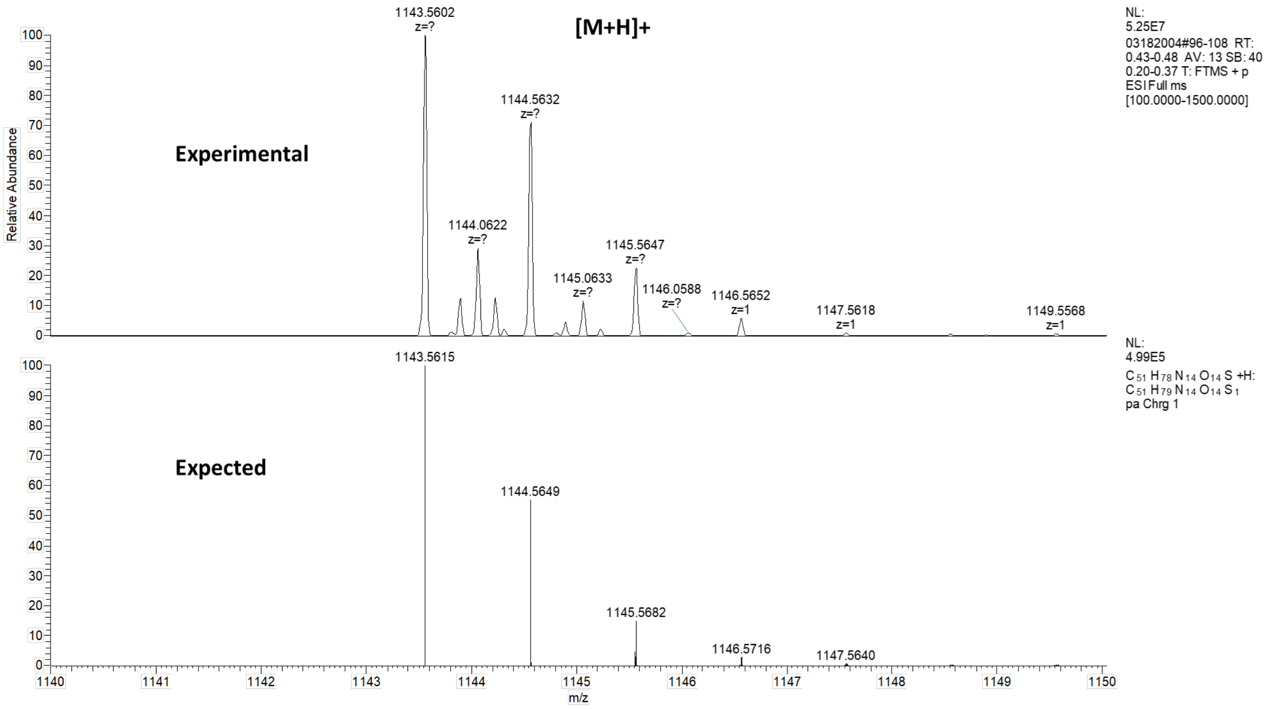

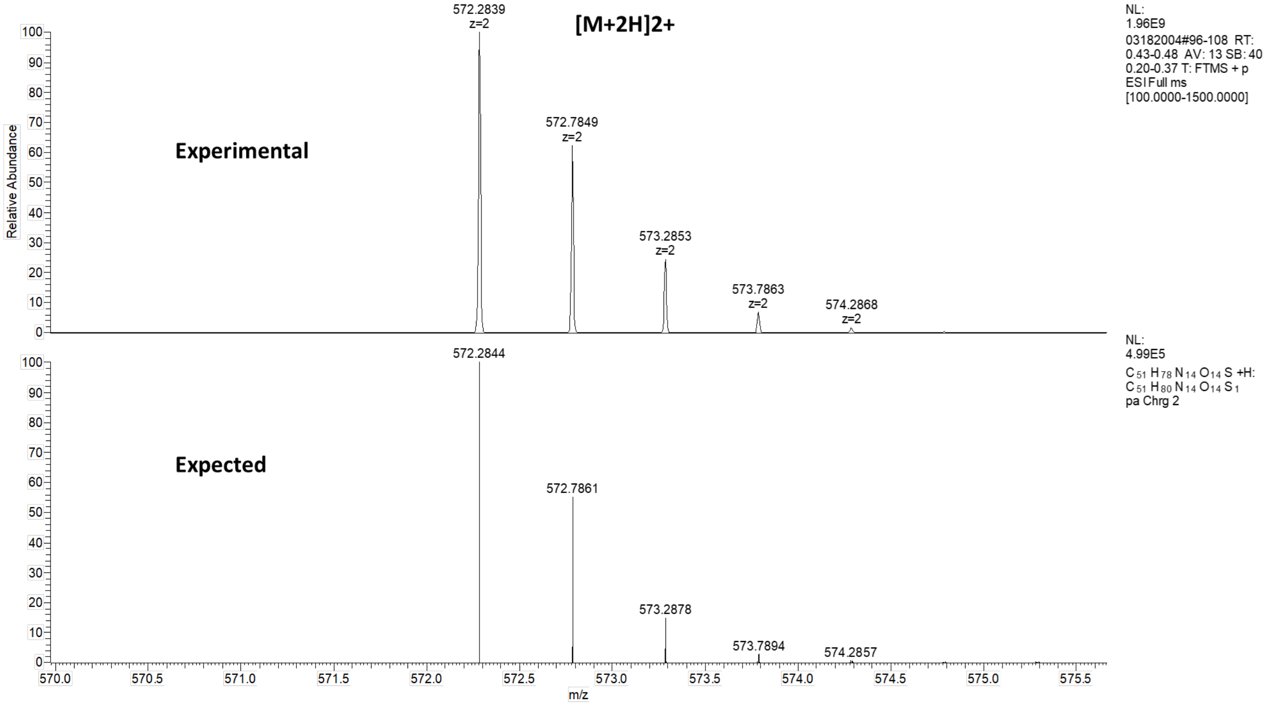

m/z calc: 1143.5615 $[M+H]^+$, 572.2844 $[M+2H]^{+2}$; m/z found: 1143.5602 $[M+H]^+$, 572.2839 $[M+2H]^{+2}$

1a(ii)ext

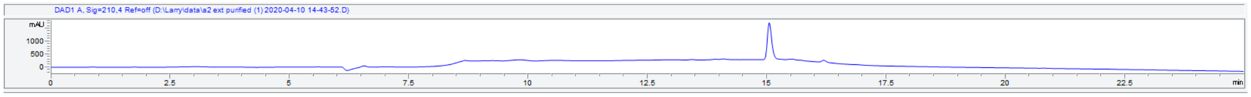

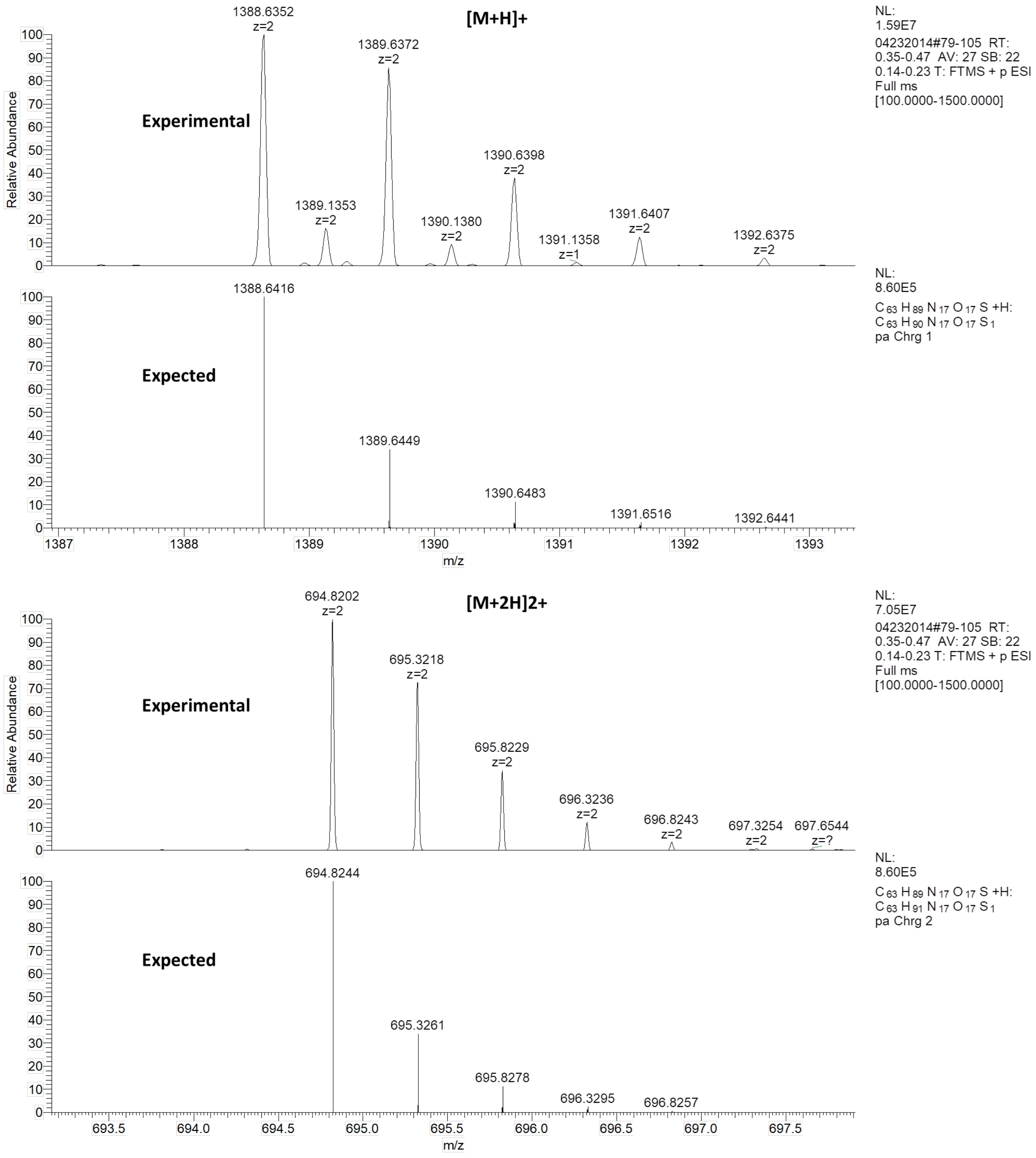

m/z calc: 1388.6416 $[M+H]^+$, 694.8244 $[M+2H]^{+2}$; m/z found: 1388.6352 $[M+H]^+$, 694.8202 $[M+2H]^{+2}$

1a(iii)

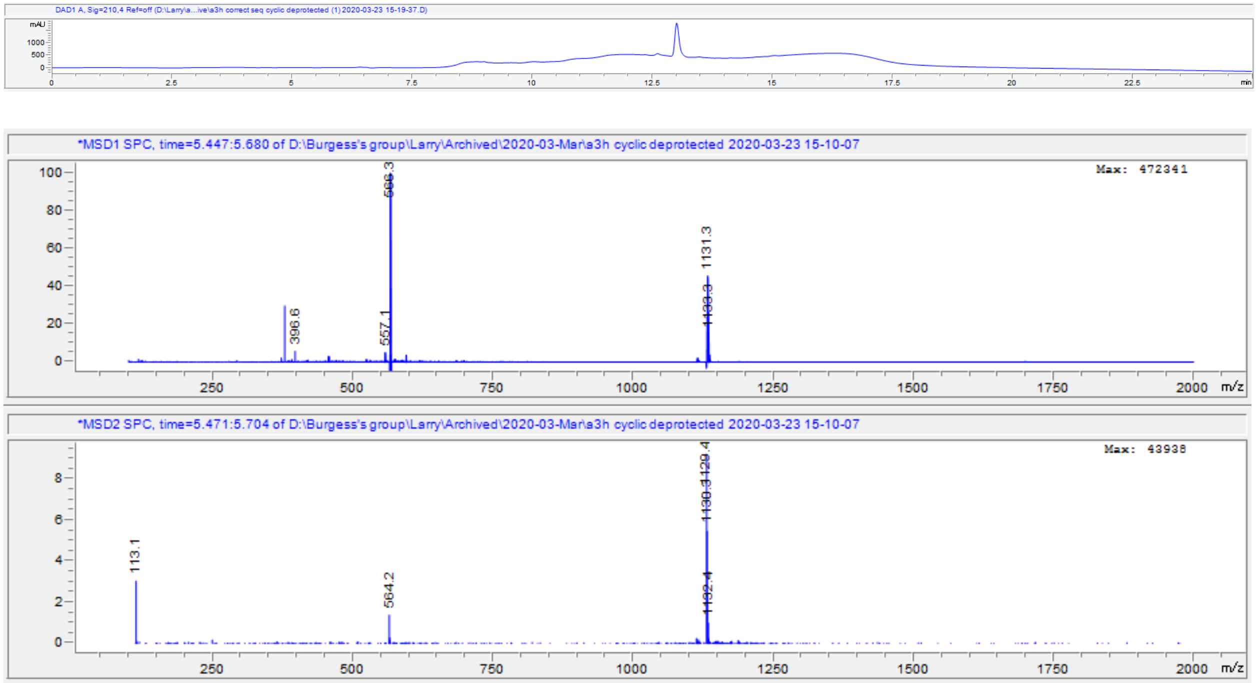

m/z calc: 1131.47 [M+H], 566.24 [M+2H]/2; m/z found: 1131.3 [M+H], 566.3 [M+2H]/2

1a(iii)mut

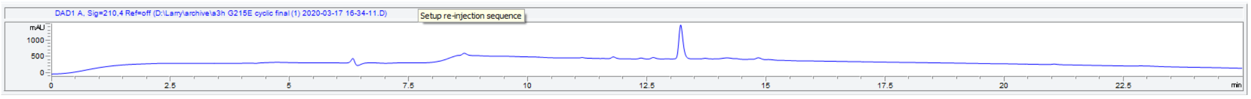

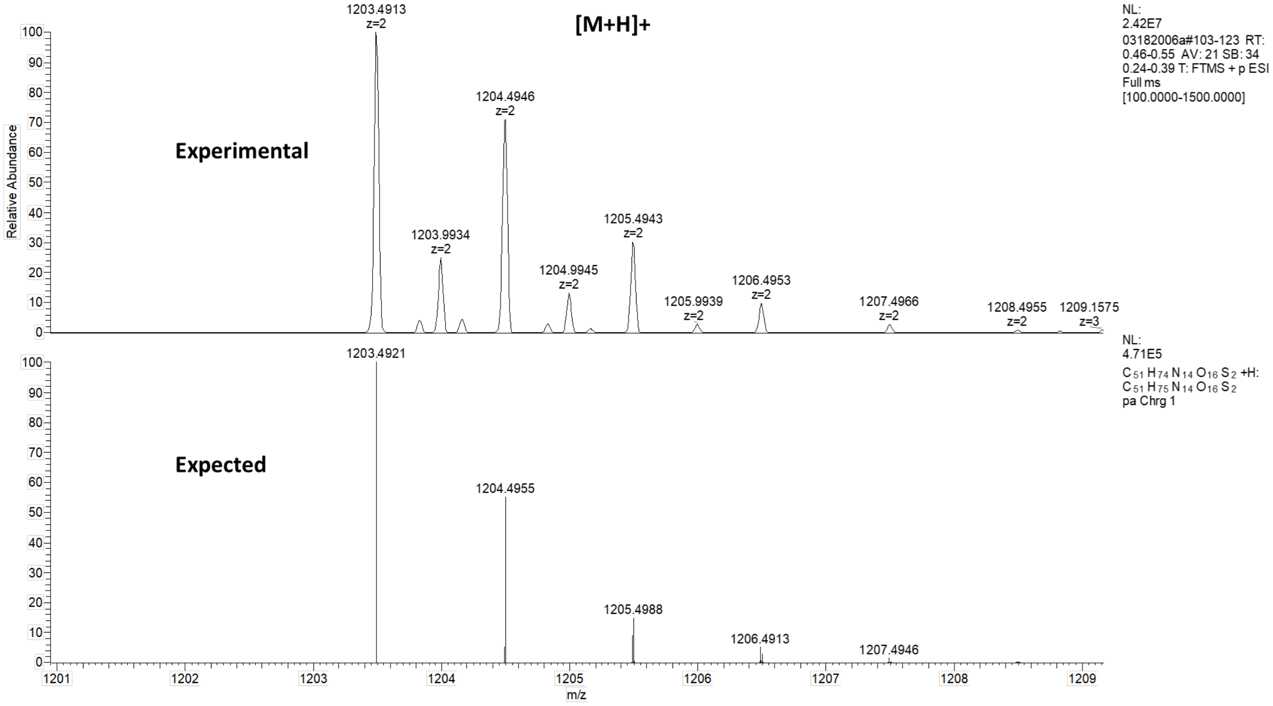

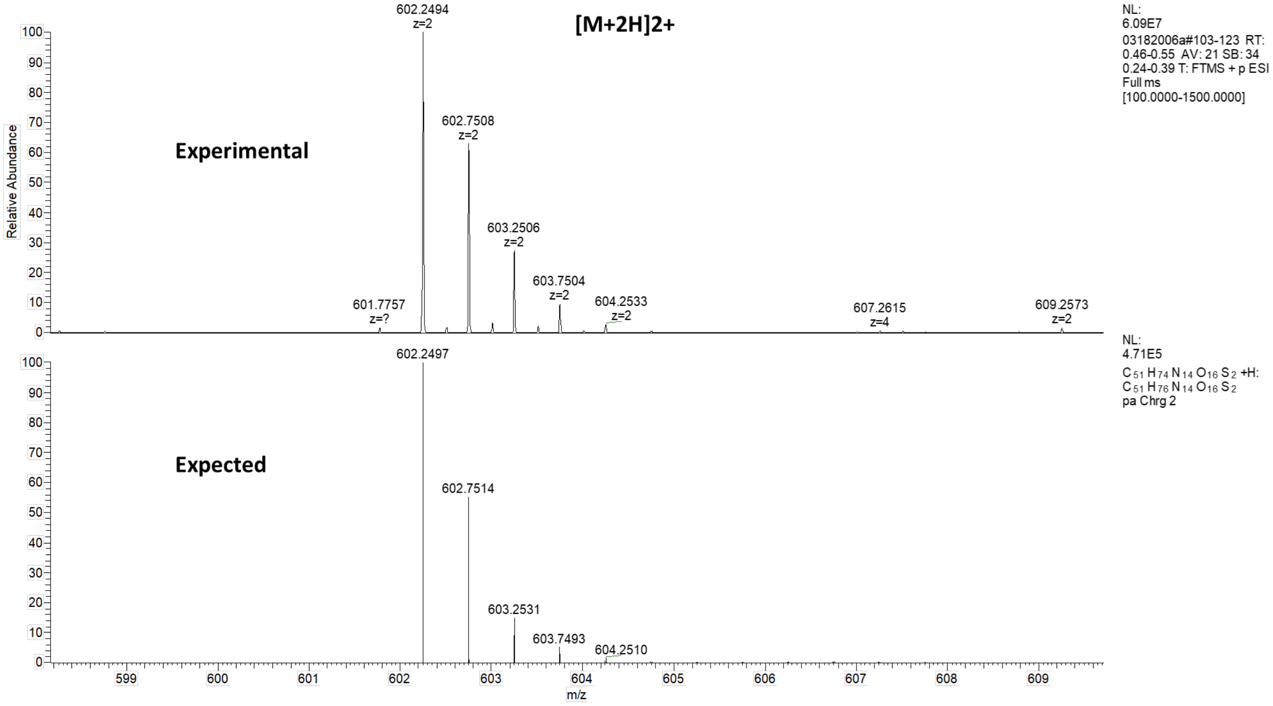

m/z calc: 1203.4921 $[M+H]^+$, 602.2497 $[M+2H]^{+2}$; m/z found: 1203.4913 $[M+H]^+$, 602.2494 $[M+2H]^{+2}$

1a(iii)mouse

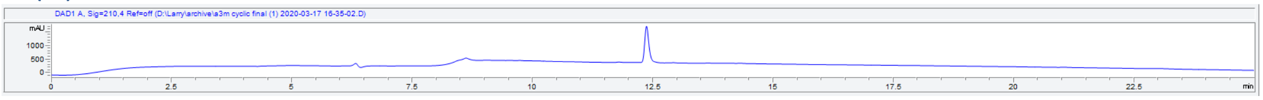

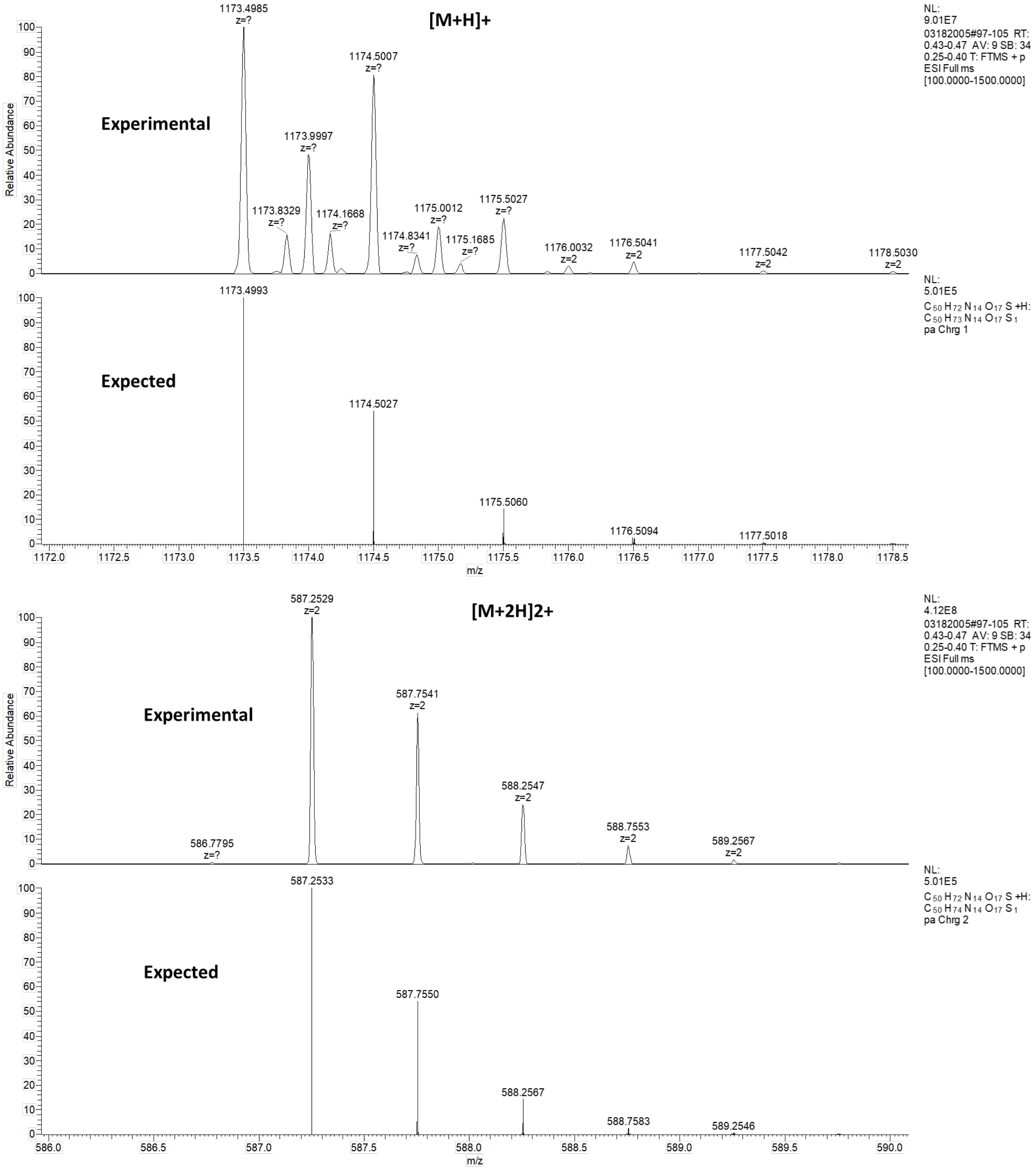

m/z calc: 1173.4993 $[M+H]^+$, 587.2533 $[M+2H]^{+2}$; m/z found: 1173.4985 $[M+H]^+$, 587.2529 $[M+2H]^{+2}$

1b(i)

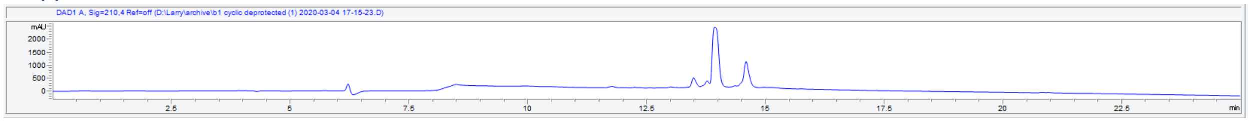

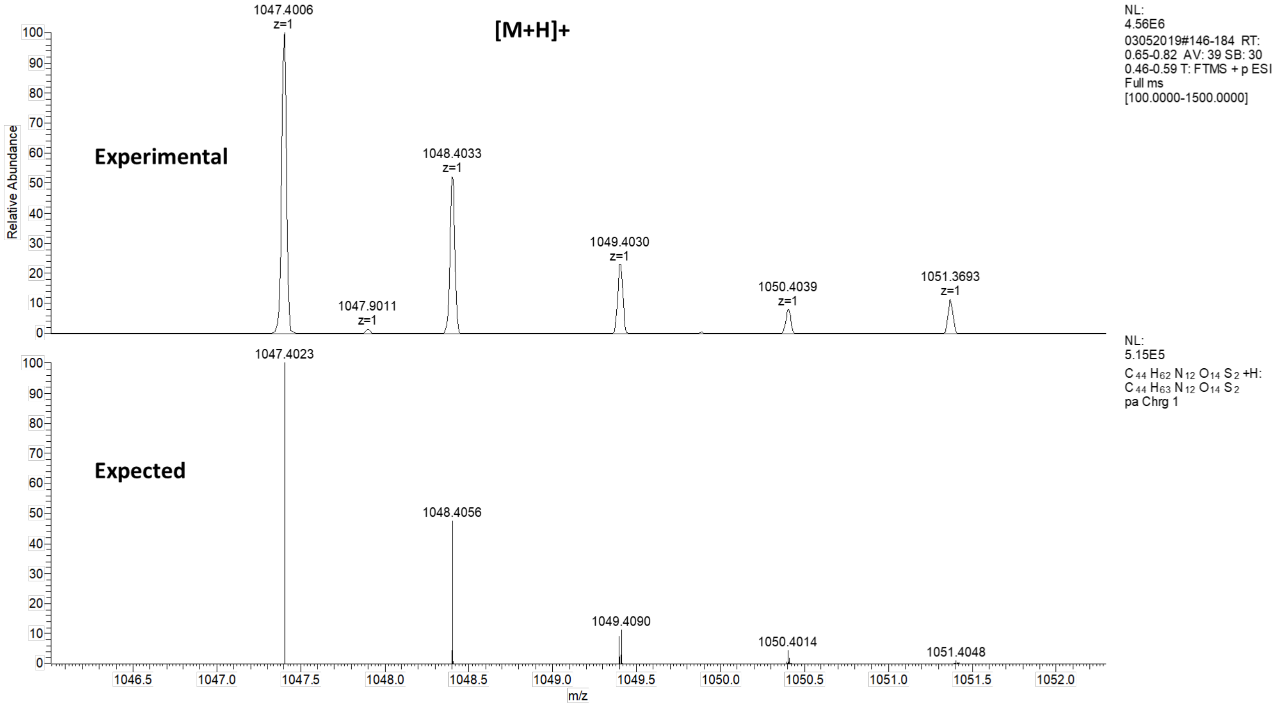

m/z calc: 1047.4023 $[M+H]^+$; m/z found: 1047.4006 $[M+H]^+$

1b(ii)

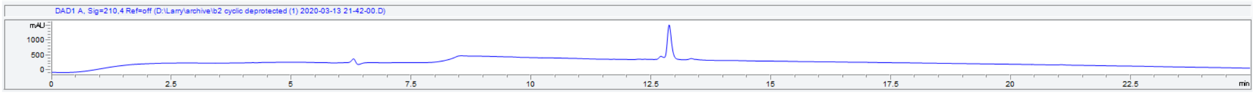

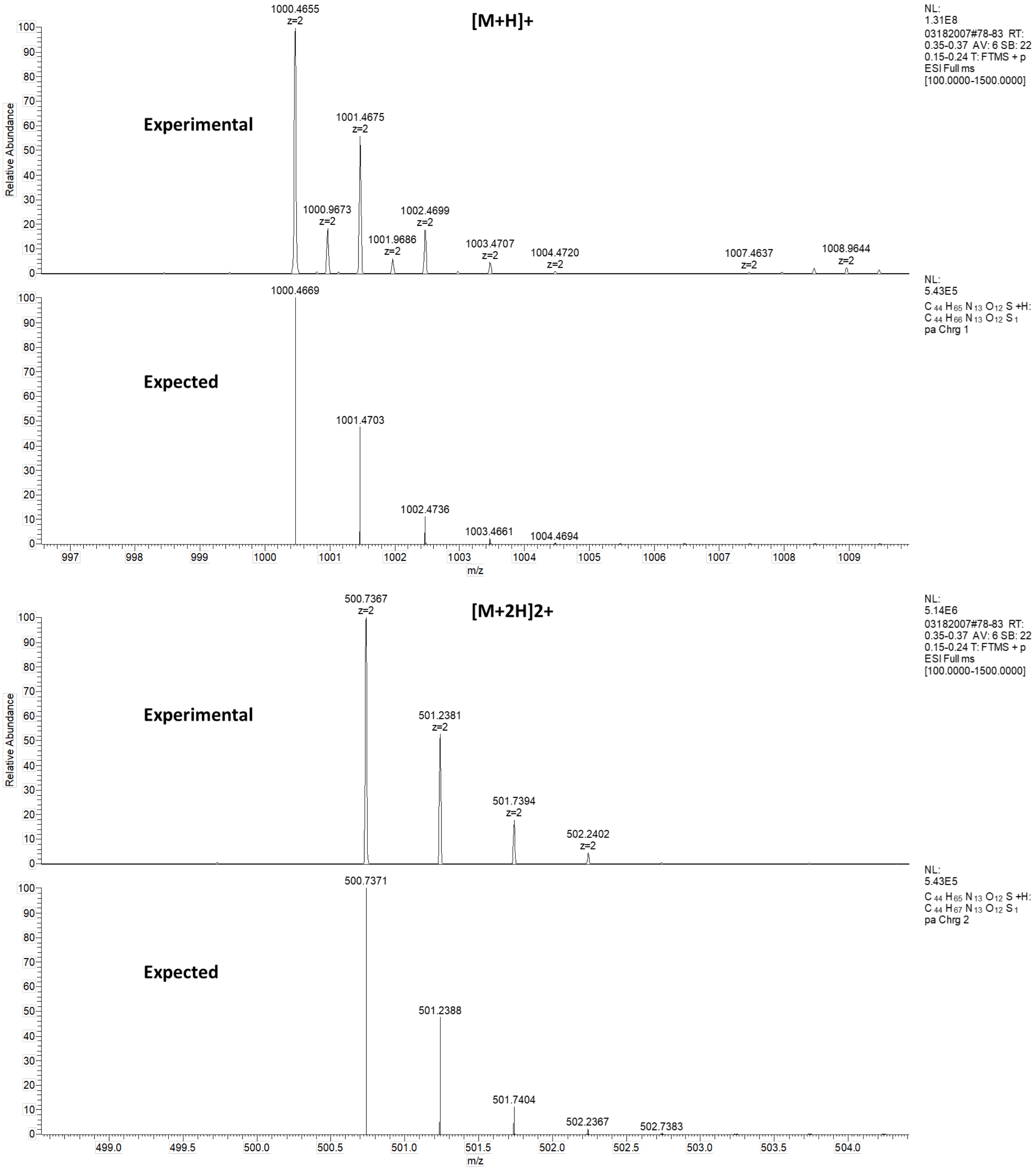

m/z calc: 1000.4669 $[M+H]^+$, 500.7371 $[M+2H]^{+2}$; m/z found: 1000.4655 $[M+H]^+$, 500.7367 $[M+2H]^{+2}$

1b(ii)lin

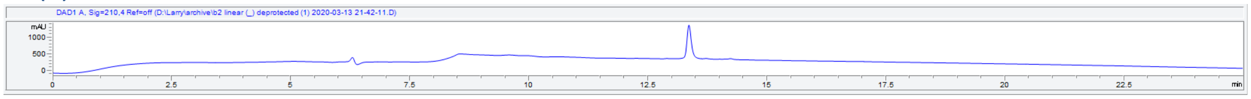

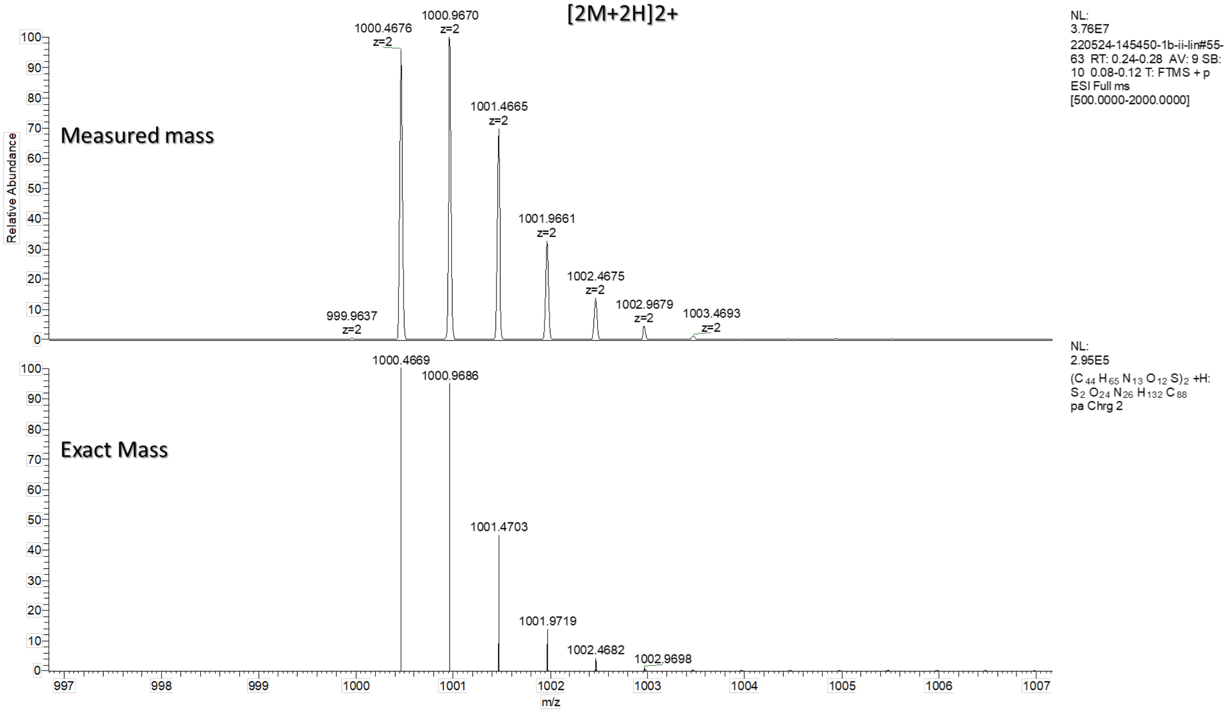

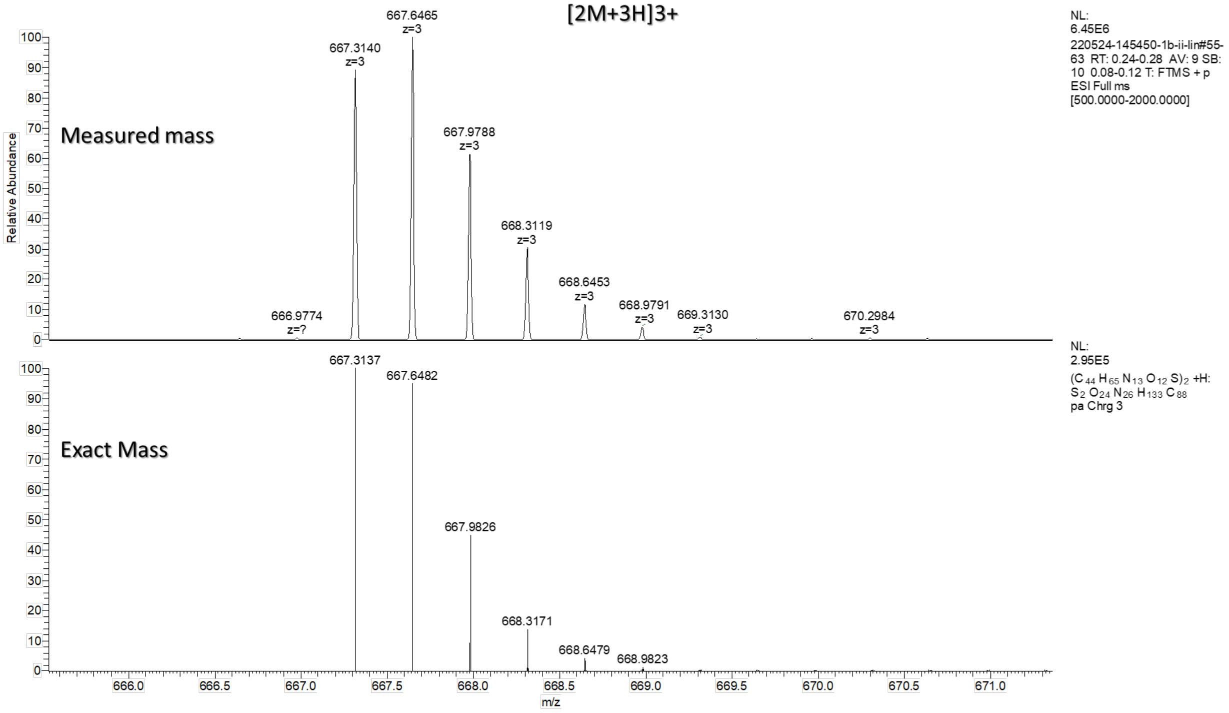

m/z calc: 1000.4669 $[2M+2H]^{+2}$, 667.3137 $[2M+3H]^{+3}$; m/z found: 1000.4676 $[2M+2H]^{+2}$, 667.3140 $[2M+3H]^{+3}$

1b(iii)

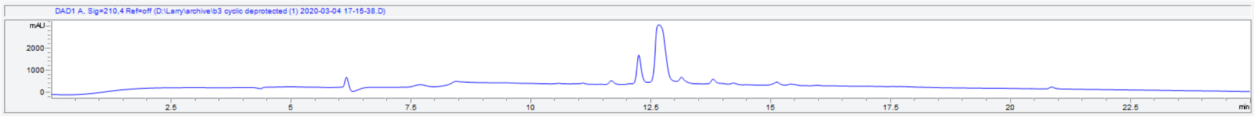

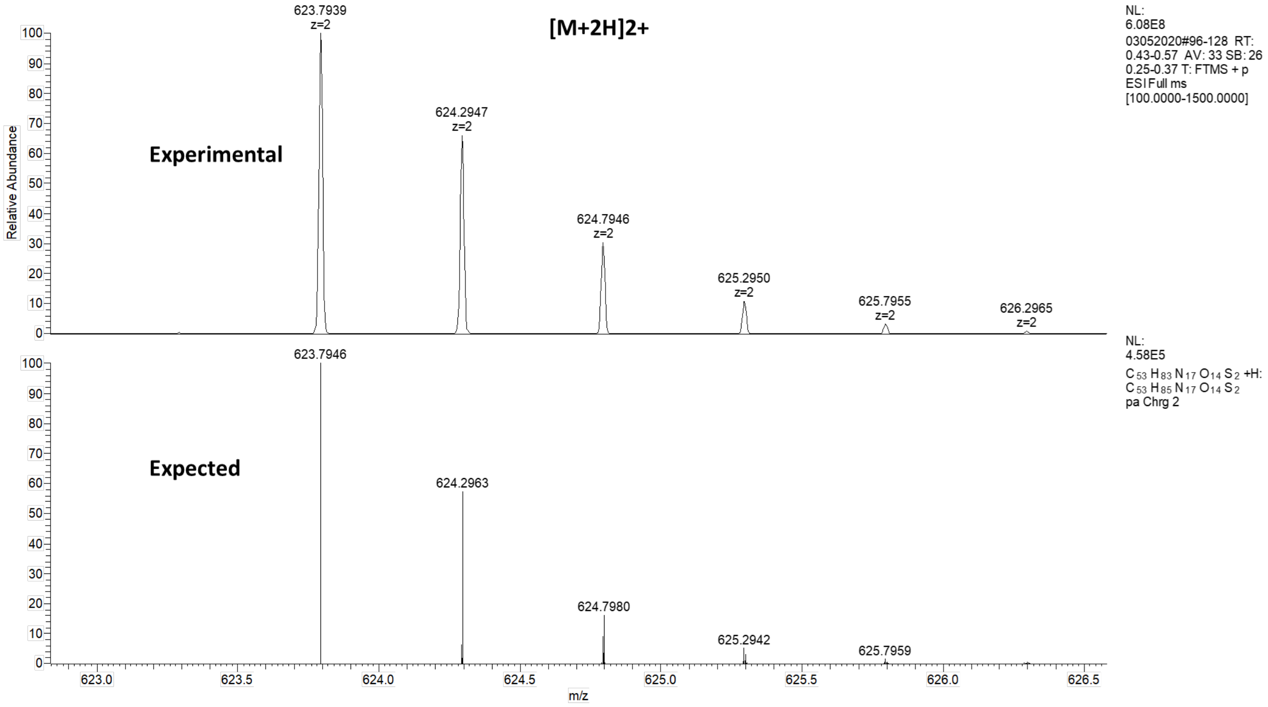

m/z calc: 623.7946 $[M+2H]^{+2}$; m/z found: 623.7939 $[M+2H]^{+2}$

1c(i)

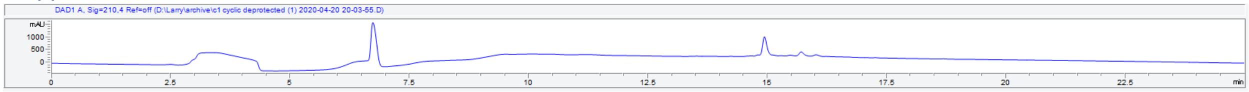

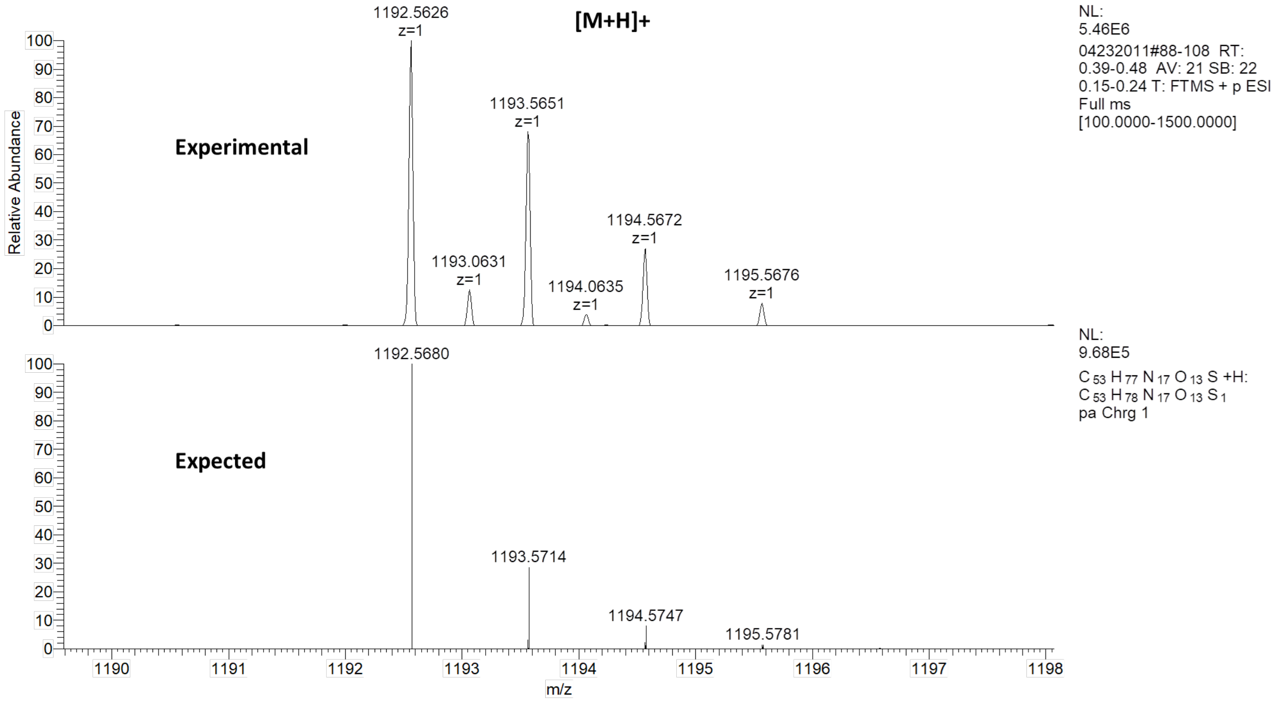

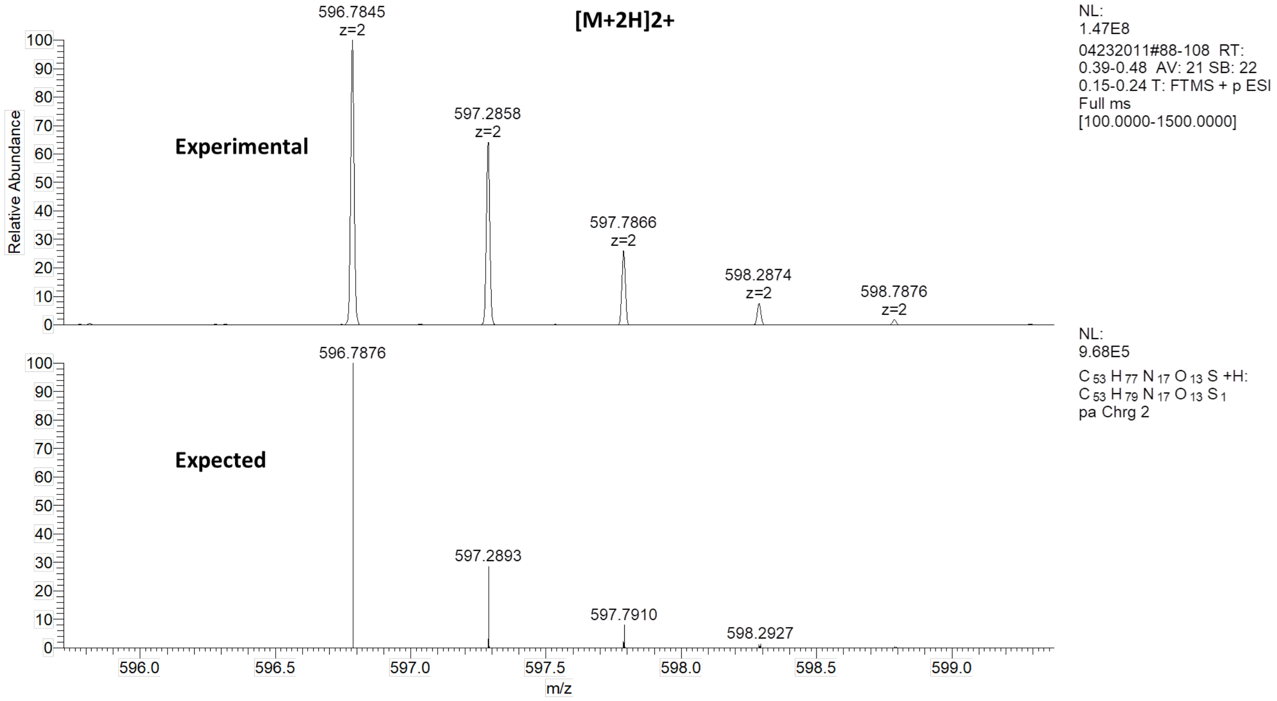

m/z calc: 1192.5680 [M+H]$^{+}$, 596.7876 [M+2H]$^{+2}$; m/z found: 1192.5626 [M+H]$^{+}$, 596.7845 [M+2H]$^{+2}$

1c(i)lin

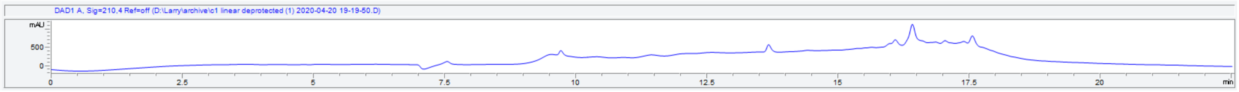

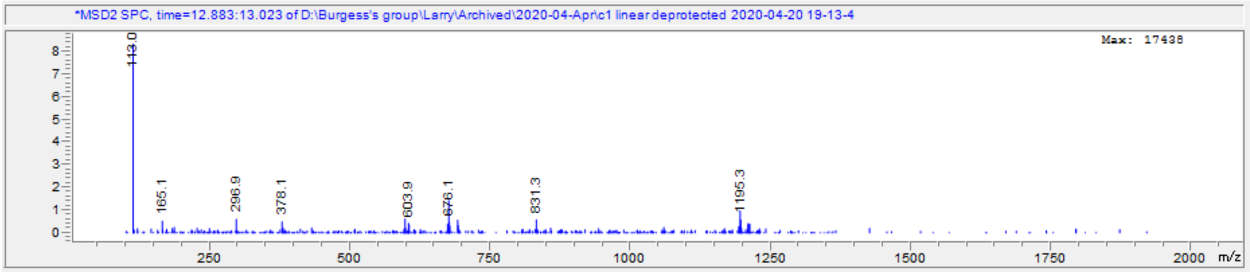

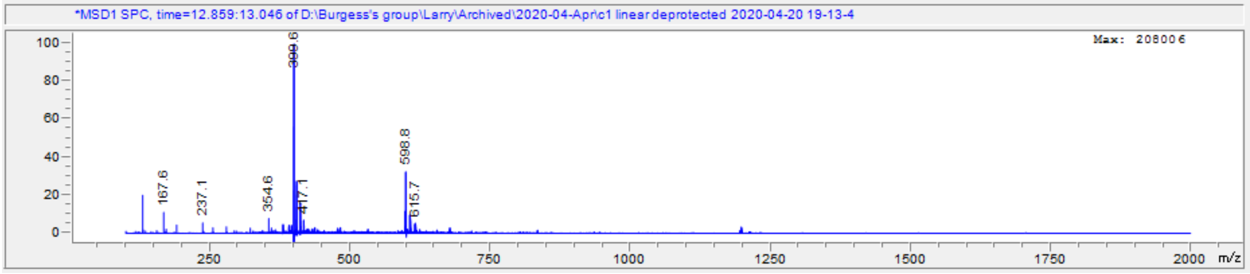

m/z calc: 596.79 $[M+2H]^{+2}$; m/z found: 598.8 $[M+2H]^{+2}$

1c(ii)dim

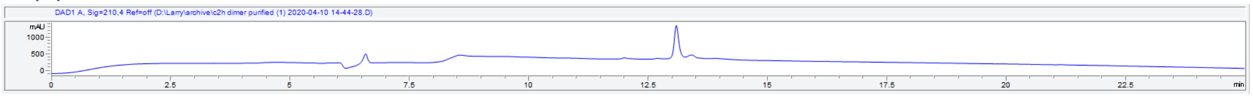

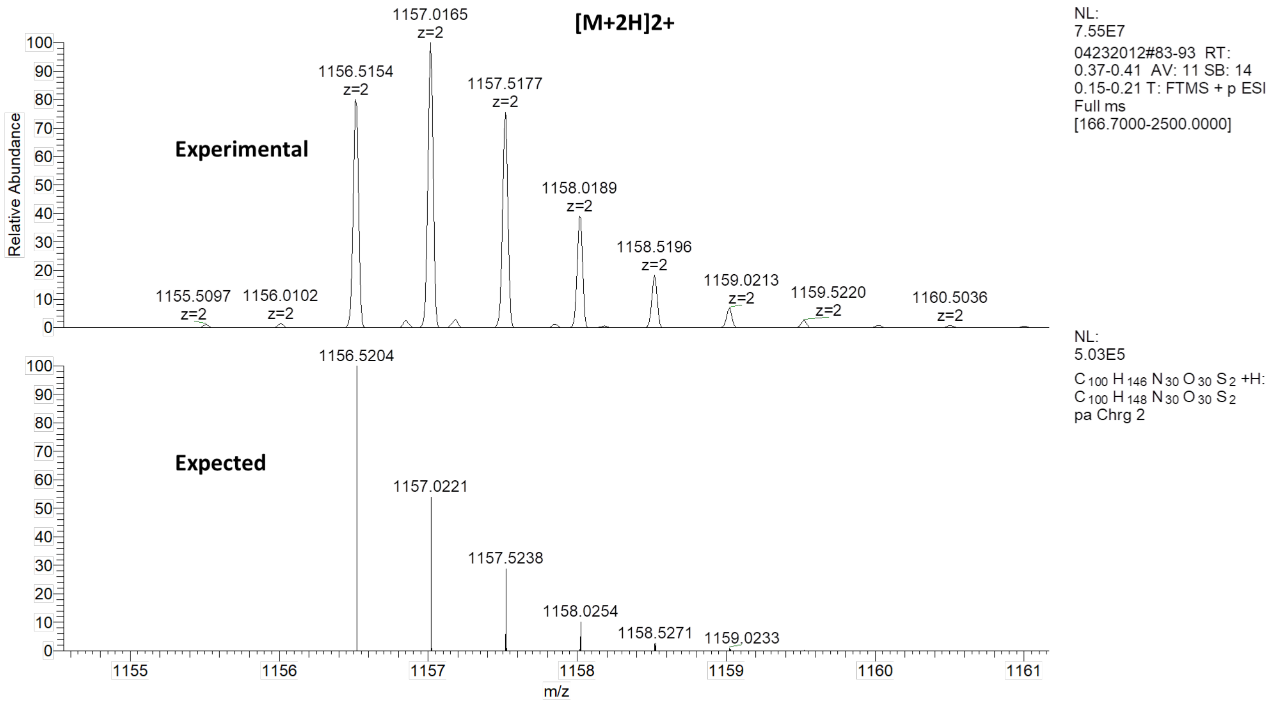

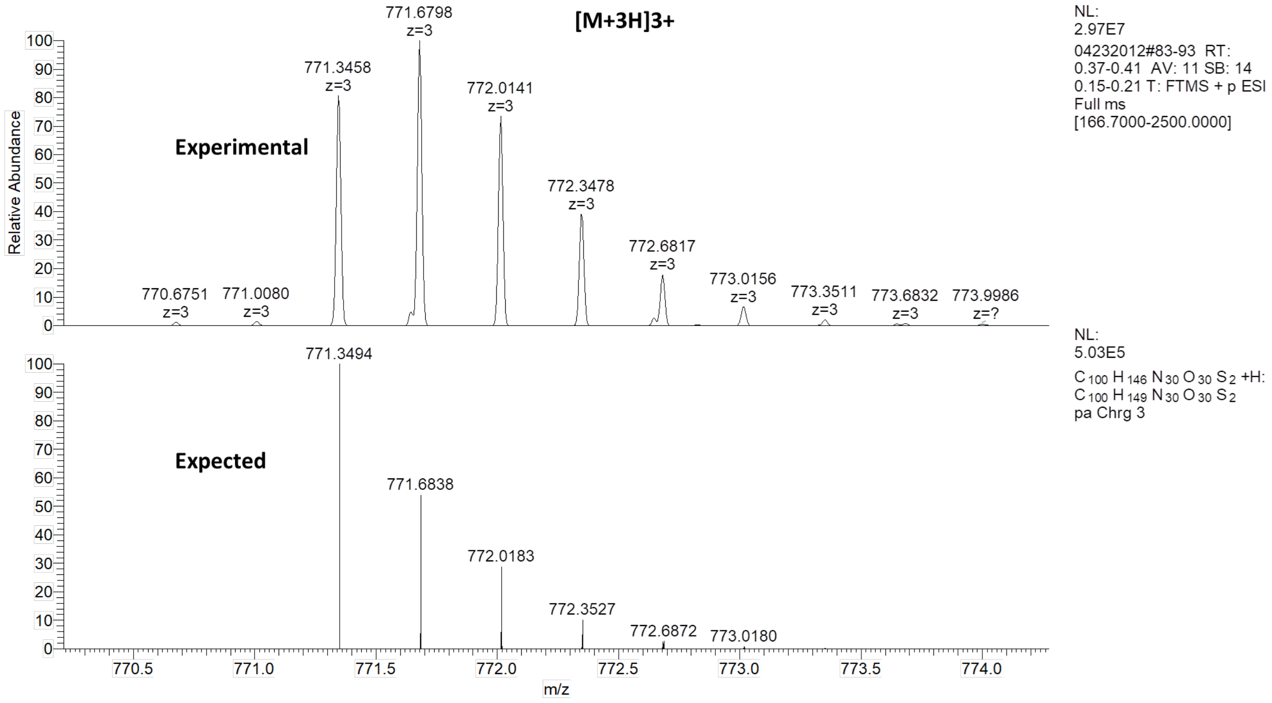

m/z calc: 1156.5204 $[M+2H]^{+2}$, 771.3494 $[M+3H]^{+3}$; m/z found: 1156.5154 $[M+2H]^{+2}$, 771.3458 $[M+3H]^{+3}$

1c(ii)ext

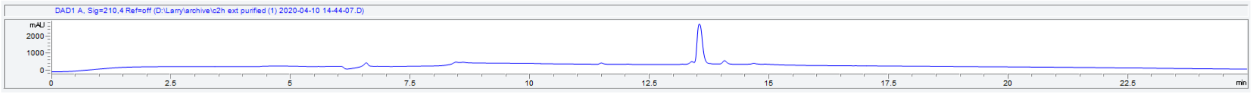

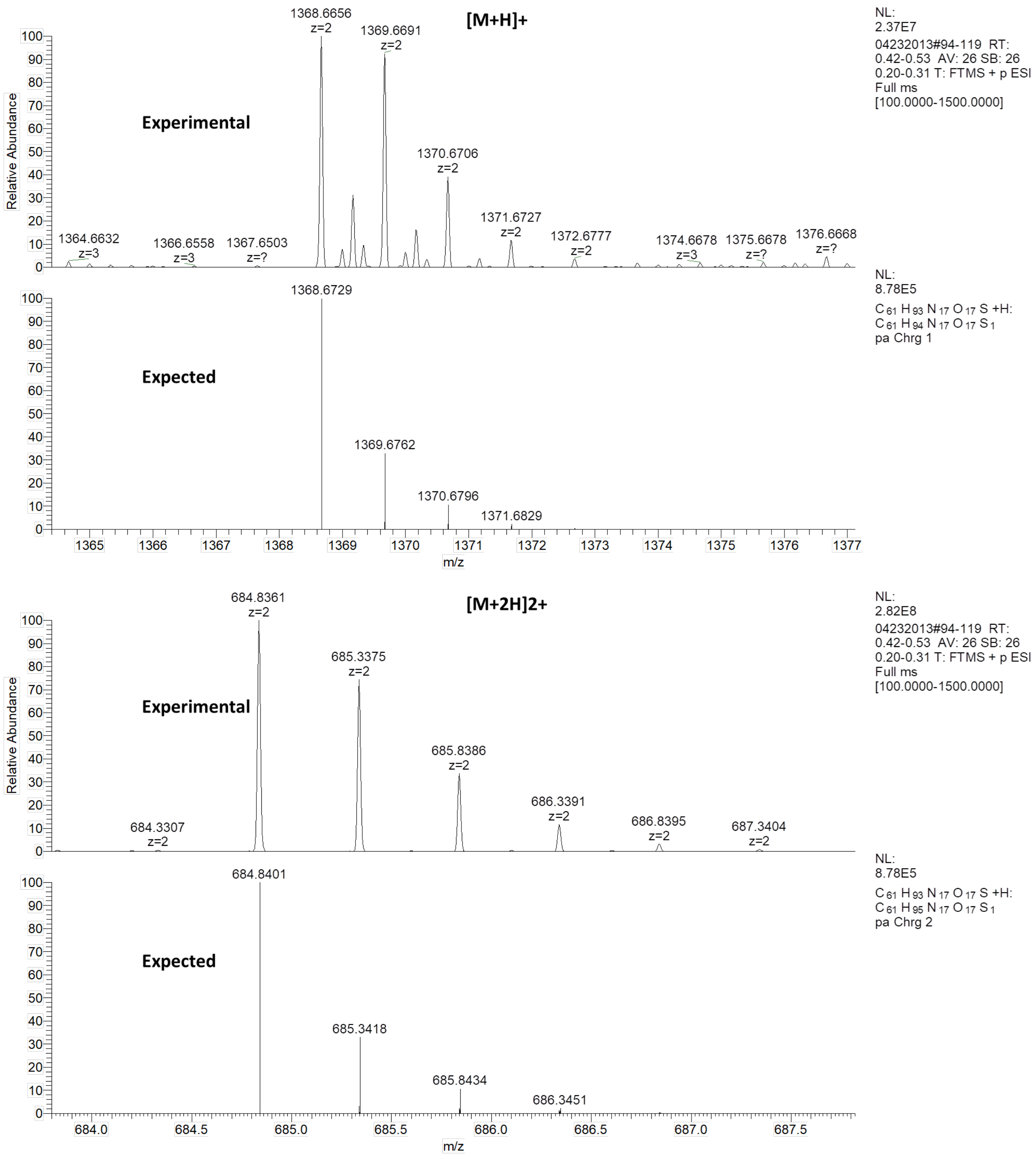

m/z calc: 1368.6729 [M+H]$^{+}$, 684.8401 [M+2H]$^{+2}$; m/z found: 1368.6656 [M+H]$^{+}$, 684.8361 [M+2H]$^{+2}$

1c(ii)mutlin

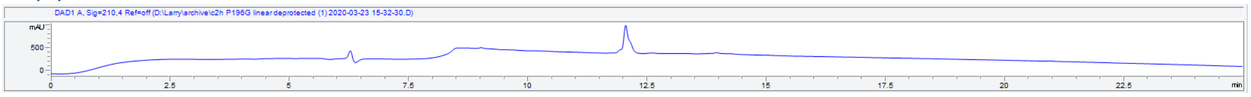

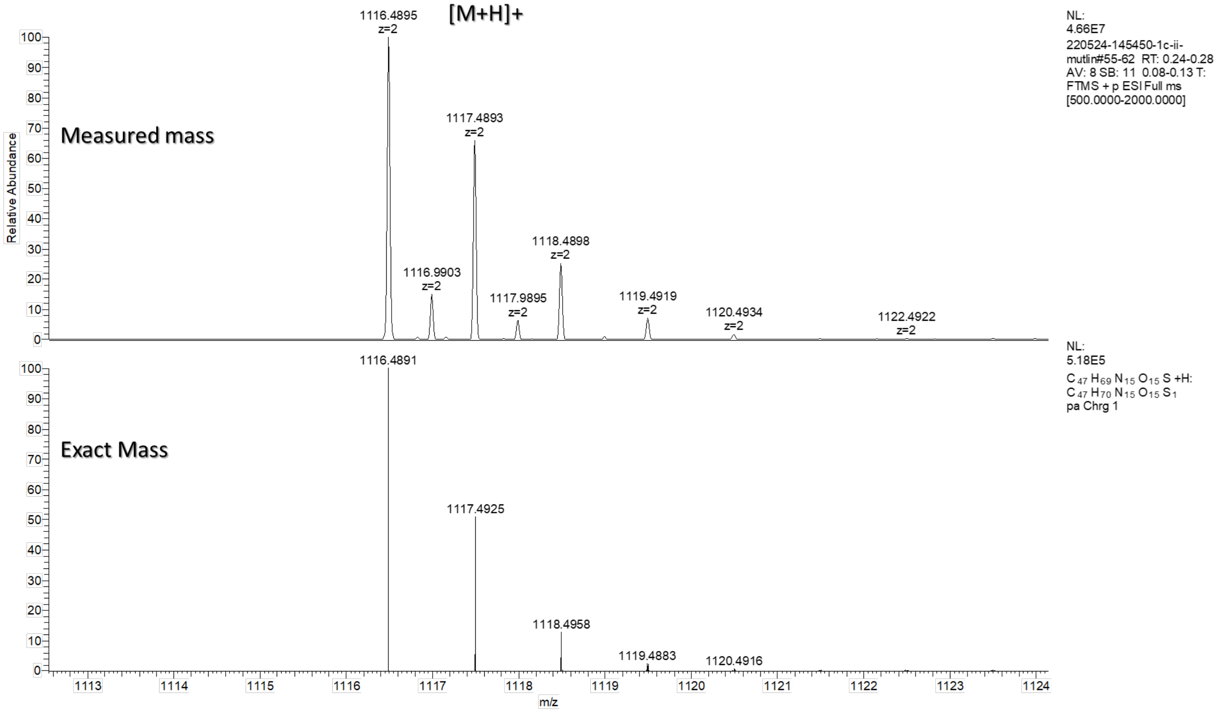

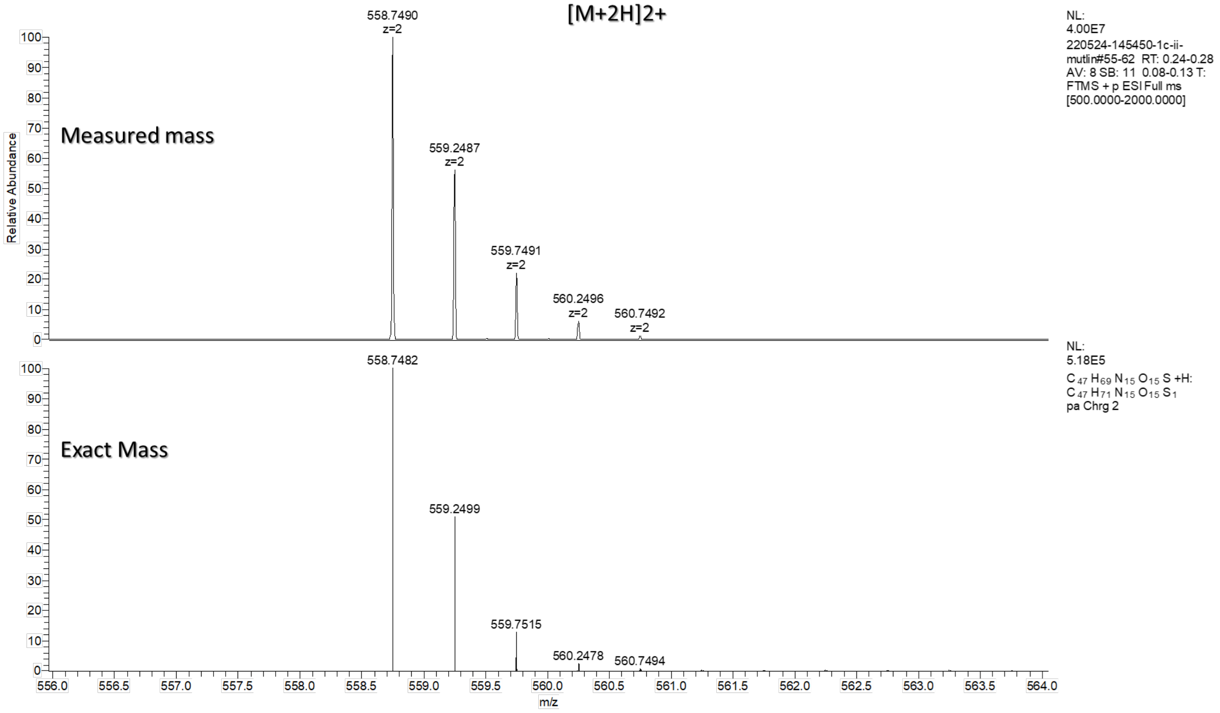

m/z calc: 1116.4891 [M+H]$^+$, 558.7482 [M+2H]$^{+2}$; m/z found: 1116.4895 [M+H]$^+$, 558.7490 [M+2H]$^{+2}$

1c(ii)mutdim

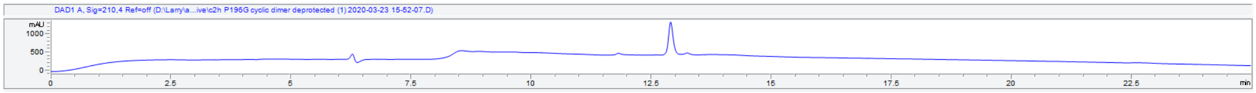

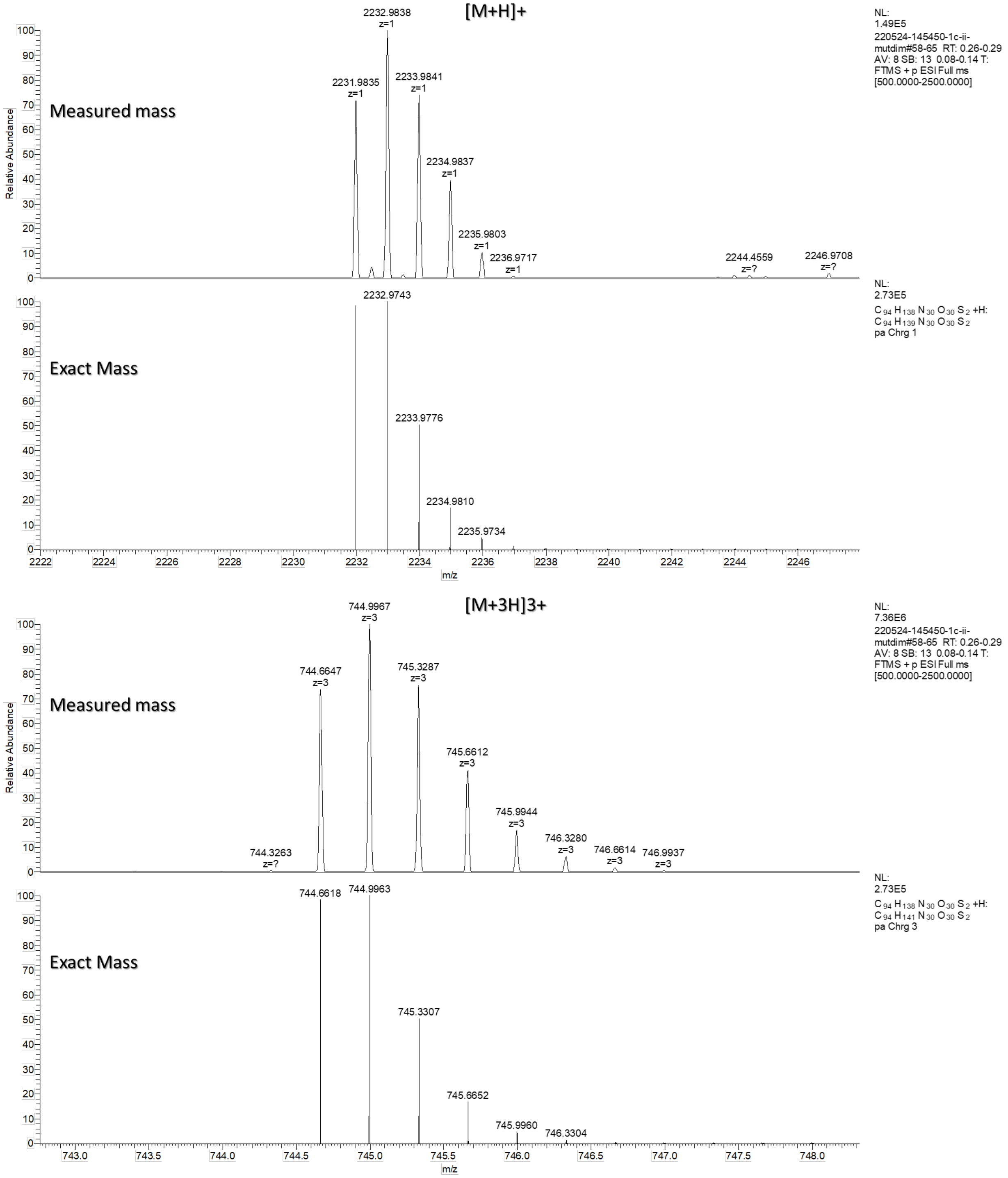

m/z calc: 2232.9743 [M+H]$^+$, 744.6618 [M+3H]$^{+3}$; m/z found: 2232.9838 [M+H]$^+$, 744.6647 [M+3H]$^{+3}$

1c(ii)mouse

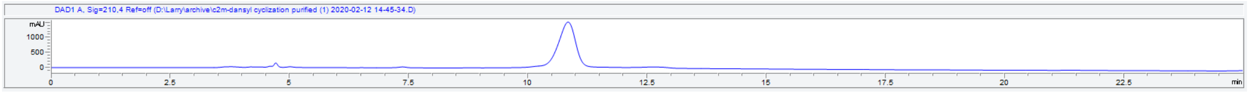

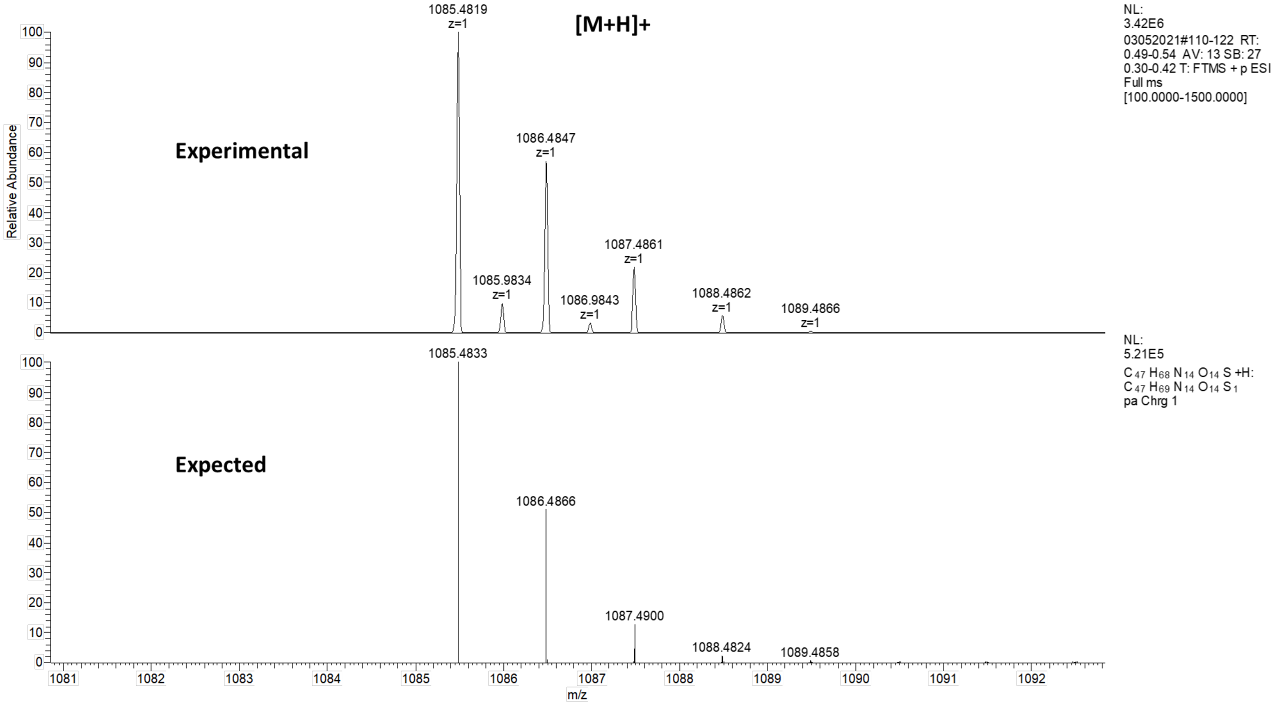

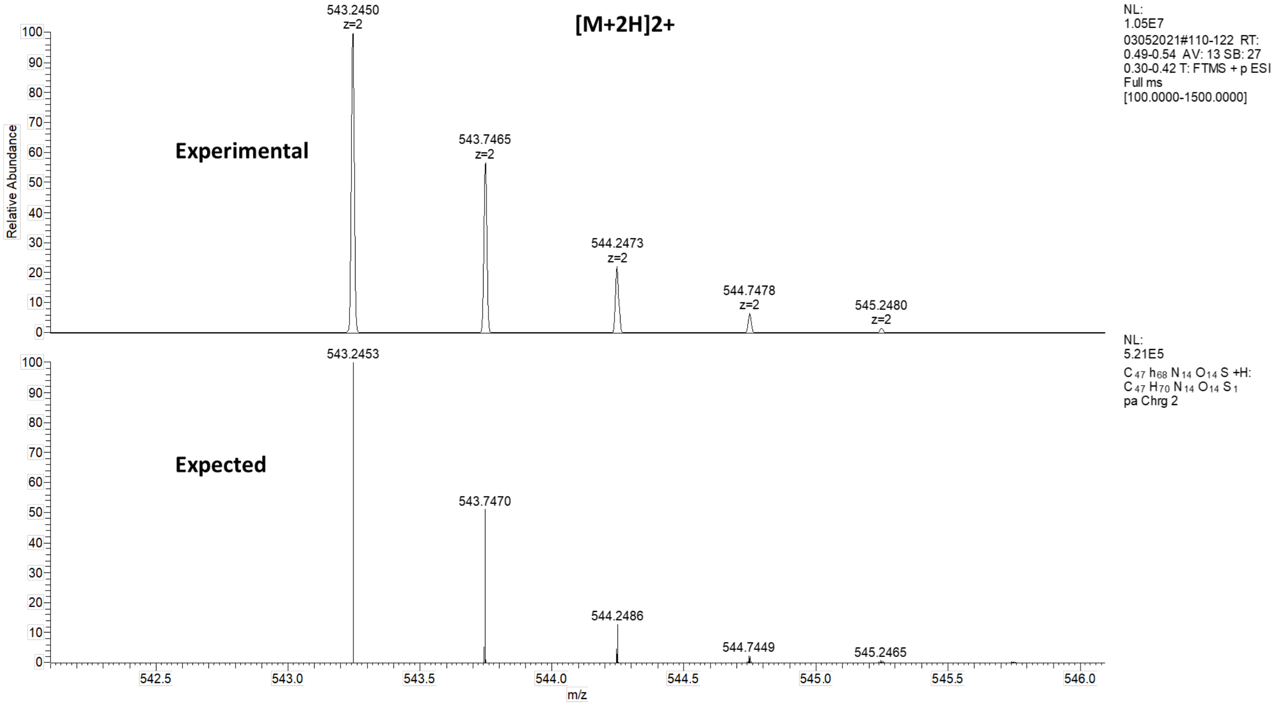

m/z calc: 1085.4833 $[M+H]^+$, 543.2453 $[M+2H]^{+2}$; m/z found: 1085.4819 $[M+H]^+$, 543.2450 $[M+2H]^{+2}$

1c(ii)mousedim

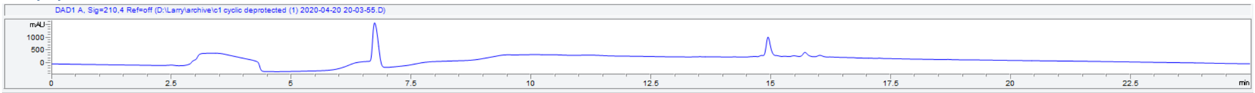

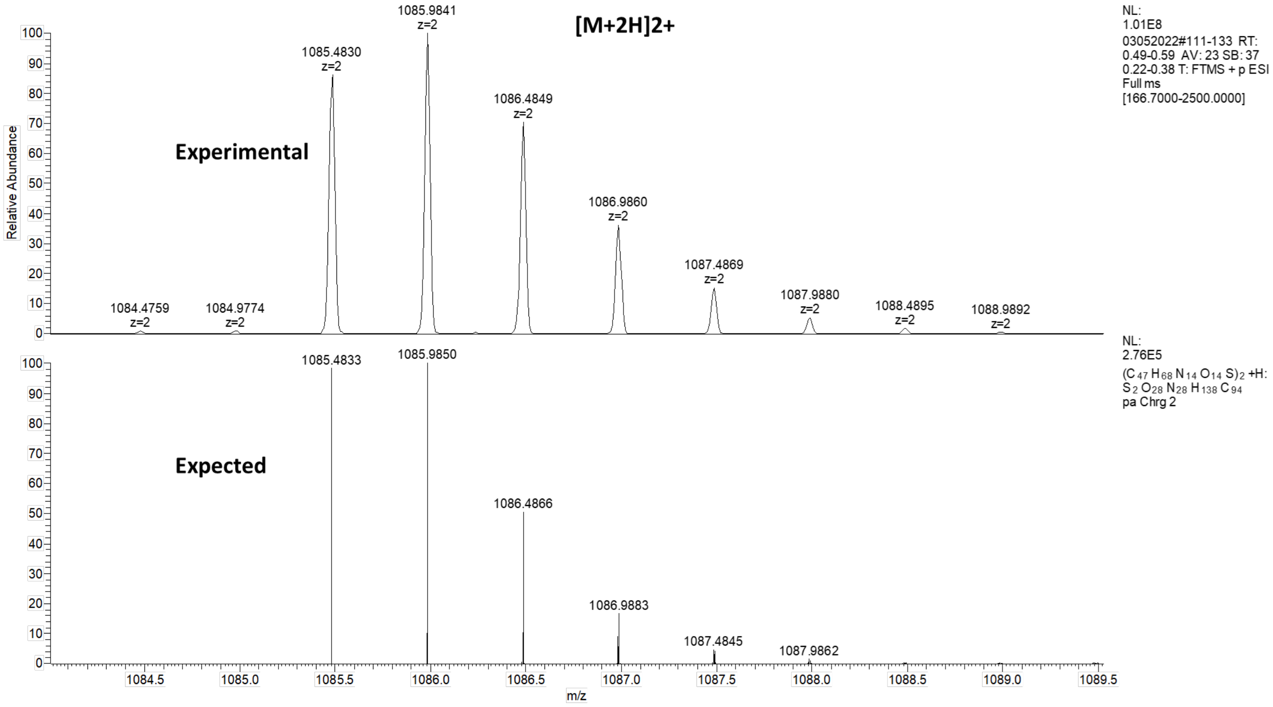

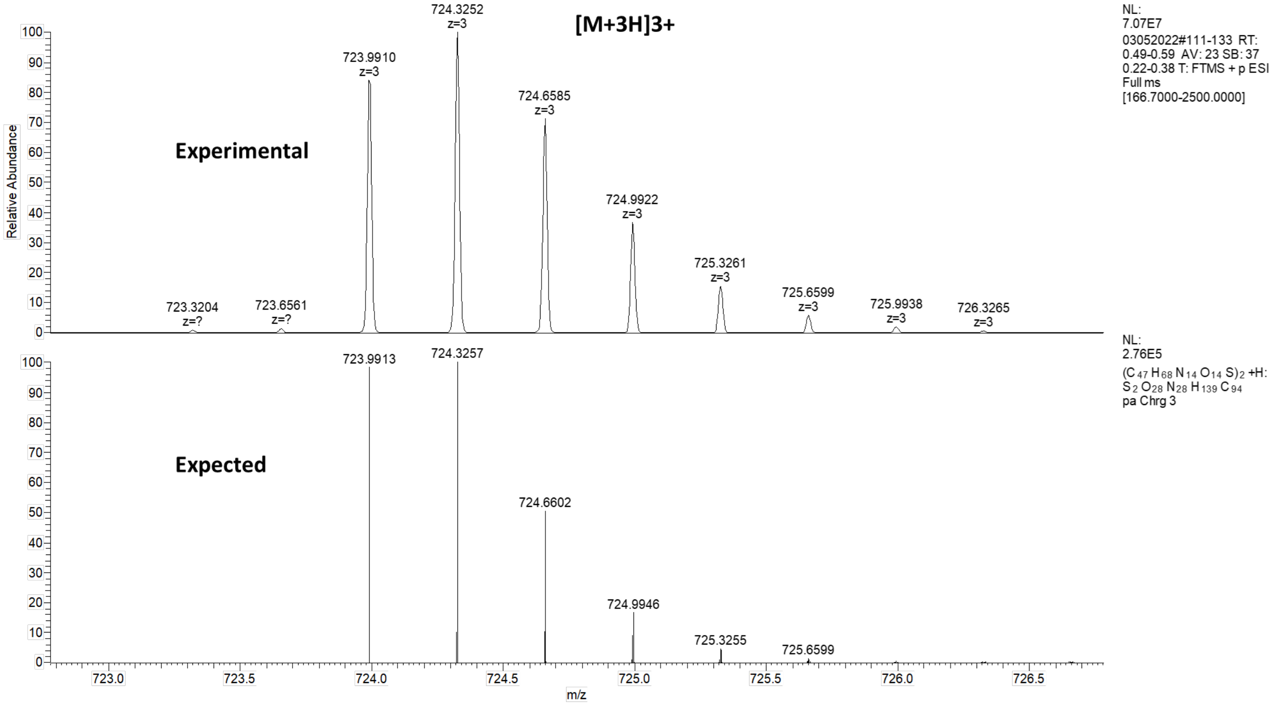

m/z calc: 1085.9850 $[M+2H]^{+2}$, 724.3257 $[M+3H]^{+3}$; m/z found: 1085.9841 $[M+2H]^{+2}$, 724.3252 $[M+3H]^{+3}$

1c(iii)

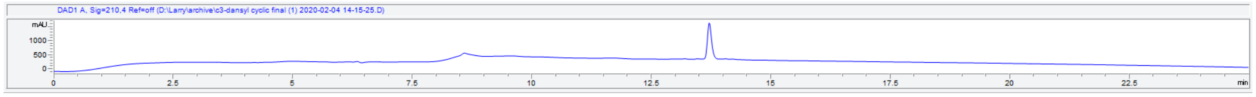

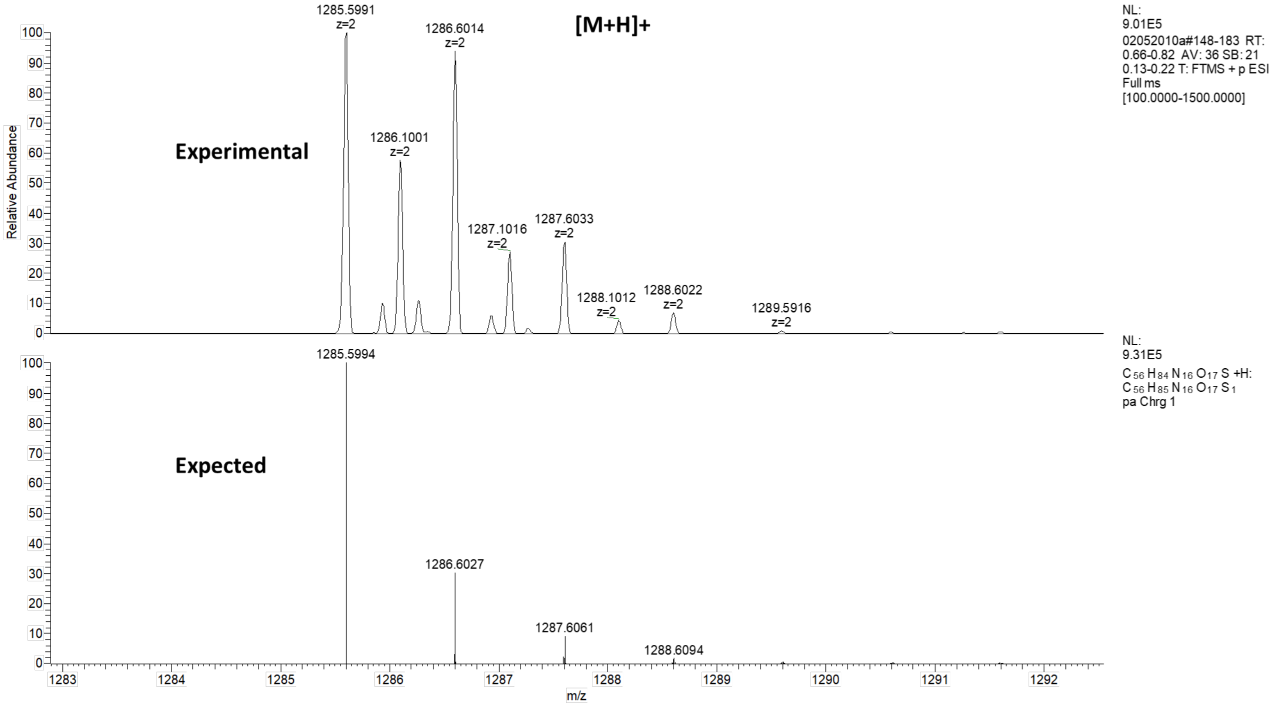

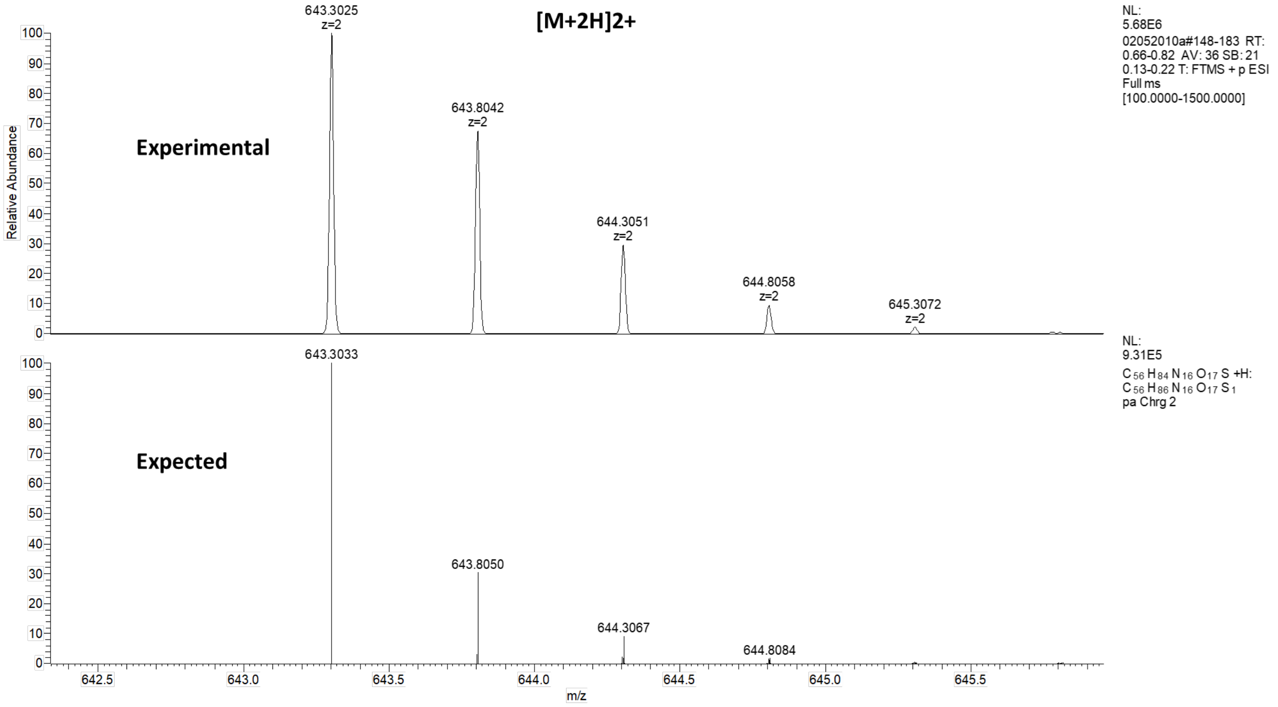

m/z calc: 1285.5994 $[M+H]^+$, 643.3033 $[M+2H]^{+2}$; m/z found: 1285.5991 $[M+H]^+$, 643.3025 $[M+2H]^{+2}$

Series 2

Compound Sequences and Structures

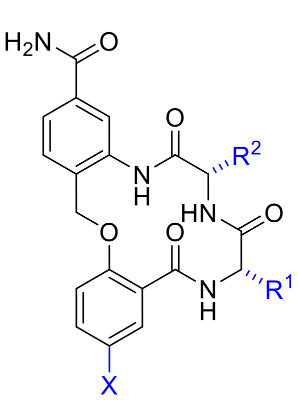

2

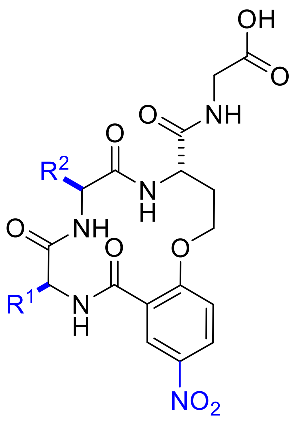

D3

name	R^1	R^2	X
**2a(i)IK**	Ile	Lys	NH_2
**2a(i)'KG**	Lys	Gly	$NHSO_2Me$
**2a(iii)GK**	Gly	Lys	NH_2
**2c(i)RG**	Arg	Gly	NH_2
**2c(i)'IR**	Ile	Arg	$NHSO_2Me$
**2c(i)'RG**	Arg	Gly	$NHSO_2Me$
**D3**	Glu	Lys	NO_2

Synthesis and Characterization

All compounds were prepared as previously reported in the literature.[4]

Series 3 and 4

Compound Sequences and Structures

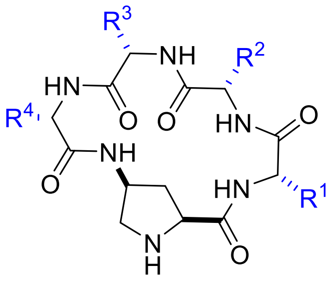

3

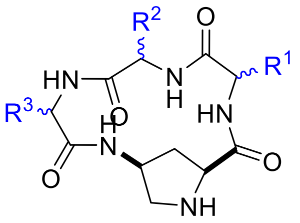

4

name	R^1	R^2	R^3	R^4
**3a(i)**	Asp	Ile	Lys	Gly
**3a(ii)**	Ile	Asn	Asn	Ser
**3a(iii)**	Asp	Gly	Lys	Gln
**3a(iii)mouse**	Asp	Glu	Lys	Gln
**3b(i)**	Asp	Met	Ser	Gly
**3b(ii)**	Val	Ser	Lys	Gly
**3b(iii)**	Asp	Ser	Lys	Lys
**3c(i)**	Asp	Ile	Arg	Gly
**3c(ii)**	Thr	Gln	Asn	Ser
**3c(ii)mouse**	Thr	Gly	Asn	Ser
**3c(iii)**	Glu	Asn	Asn	Ser

name	R^1	R^2	R^3
**4a(ii)INS**	Ile	Asn	Ser
**4a(ii)snv**	D-Ser	D-Asn	D-Val
**4a(ii)Vsn**	Val	D-Ser	D-Asn
**4b(iii)DSK**	Asp	Ser	Lys
**4b(iii)SKk**	Ser	Lys	D-Lys
**4b(iii)sKk**	D-Ser	Lys	D-Lys
**4b(iii)Kks**	Lys	D-Lys	D-Ser
**4c(iii)ENK**	Glu	Asn	Lys
**4c(iii)nKV**	D-Asn	Lys	Val
**4c(iii)vkN**	D-Val	Lys	Asn
**4c(iii)Nne**	Asn	D-Asn	D-Glu
**4c(iii)ENn**	Glu	Asn	D-Asn

General Procedure for the Synthesis of Series 3 and 4 Compounds

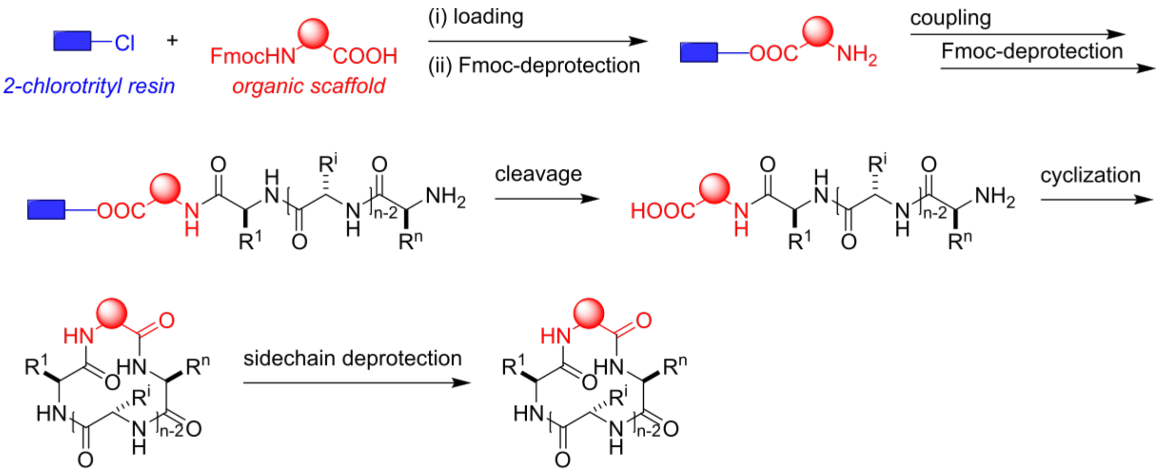

conditions:
loading: DIPEA, DMF, 50 °C, 30 min, microwave
Fmoc-deprotection: 20% Piperidine/DMF, 50 °C, 10 min, microwave
coupling: Oxyma, DIC, 50 °C, 15 min, microwave
cleavage: 20% HFIP/DCM, 25 °C, 2 h
cyclization: HATU, HOAT, 2,4,6-collidine, DMF, 25 °C, 8h
sidechain deprotection: 95% TFA, 2.5% H_2O, 2.5% TIPS, 25 °C, 2h

The organic scaffold (*N*-Boc-*cis*-4-*N*-Fmoc-amino-L-proline, 0.48 mmol) [5,6] was dissolved in DMF (4 mL), and DIPEA (1.2 mmol) was added. Half of the solution was added to a syringe with 2-chlorotrityl resin (0.2 mmol), and the mixture was microwaved at 50 °C for 30 min. The used solution was drained, and the left solution was added. The mixture was microwaved at 50°C for 30 minutes, and the solution inside the syringe was drained. Loaded resin was washed with DMF (2 mL) 3 times. 20% Piperidine/DMF (2 mL) was added to the syringe and microwaved at 50 °C for 10 min. The used solution was drained. The same procedure was repeated once to fully deprotect the Fmoc group. The resin was washed with DMF (2 mL) 3 times before the next step.

The following couplings and deprotection of regular Fmoc-protected amino acids were implemented on a peptide synthesizer (Liberty Blue, CEM). The reaction scale was set as 0.25 mmol. As for coupling, Oxyma (activator base, 1.0 M, 1 mL), DIC (activator, 0.5 M, 2 mL), Fmoc-amino acid (0.2 M, 5 mL), and resin were mixed and microwaved at 50 °C for 15 min. The used solution was drained, and the resin was washed with DMF (2 mL) for 3 min. As for deprotection, 20% Piperidine/DMF (5 mL) was added into the reaction vessel and microwaved at 50 °C for 10 min. The used solution was drained, and the resin was washed with DMF (2 mL) for 3 min.

After repeated cycles of coupling and deprotection, the resin-linked linear peptide was transferred back to a syringe. 20% HFIP/DCM (3 mL) was added and shaken for 3 h to cleave the peptide from the resin. The solution was stored in a round bottle flask (250 mL), and solvents (HFIP, DCM) were removed by constant N_2 flow. HATU (0.6 mmol), HOAT (0.6 mmol), 2,4,6-collidine (0.6 mmol), and DMF (60 mL) were added to the flask. The mixture was stirred for 8 h to cyclize the linear peptide. After the reaction was completed, DMF was removed by high-vacuum rotavapor. Water/acetonitrile (2~3 mL) was added to dissolve the remaining oils, and the sidechain-protected cyclic peptide was purified by prepHPLC. The purified cyclic peptide is dissolved in 95% TFA/ 2.5% H_2O/ 2.5% TIPS and stirred for 3 h to deprotect all remaining protecting groups. TFA was removed by constant N_2 flow, and the deprotected compound was dissolved by water/acetonitrile (2~3 mL). Crude peptide was further purified by prepHPLC to yield a pure product.

Retention times were from analytical HPLC runs using a Zorbax SB-C18 column (Agilent) with a 20 minute gradient between 5% solvent A (99.9% water, 0.1% TFA) and 95% solvent B (99.9% acetonitrile, 0.1% TFA), and 95% solvent A and 5% solvent B. Expected masses of peptides were calculated in ChemDraw. MS were obtained from ESI-MS. The ^{1}H, ^{13}C, and TOCSY-NMR spectra were recorded on Bruker Avance III at 400 MHz at room temperature in solvent of 90% H_2O + 10% D_2O. Chemical shifts of ^{1}H NMR spectra were reported in ppm as follows: chemical shift, multiplicity (s = singlet, d = doublet, t = triplet, m = multiplet, dd = doublet of doublet), coupling constants, and number of protons.

NMR Characterization of 4c(iii)nKV

4c(iii)nKV

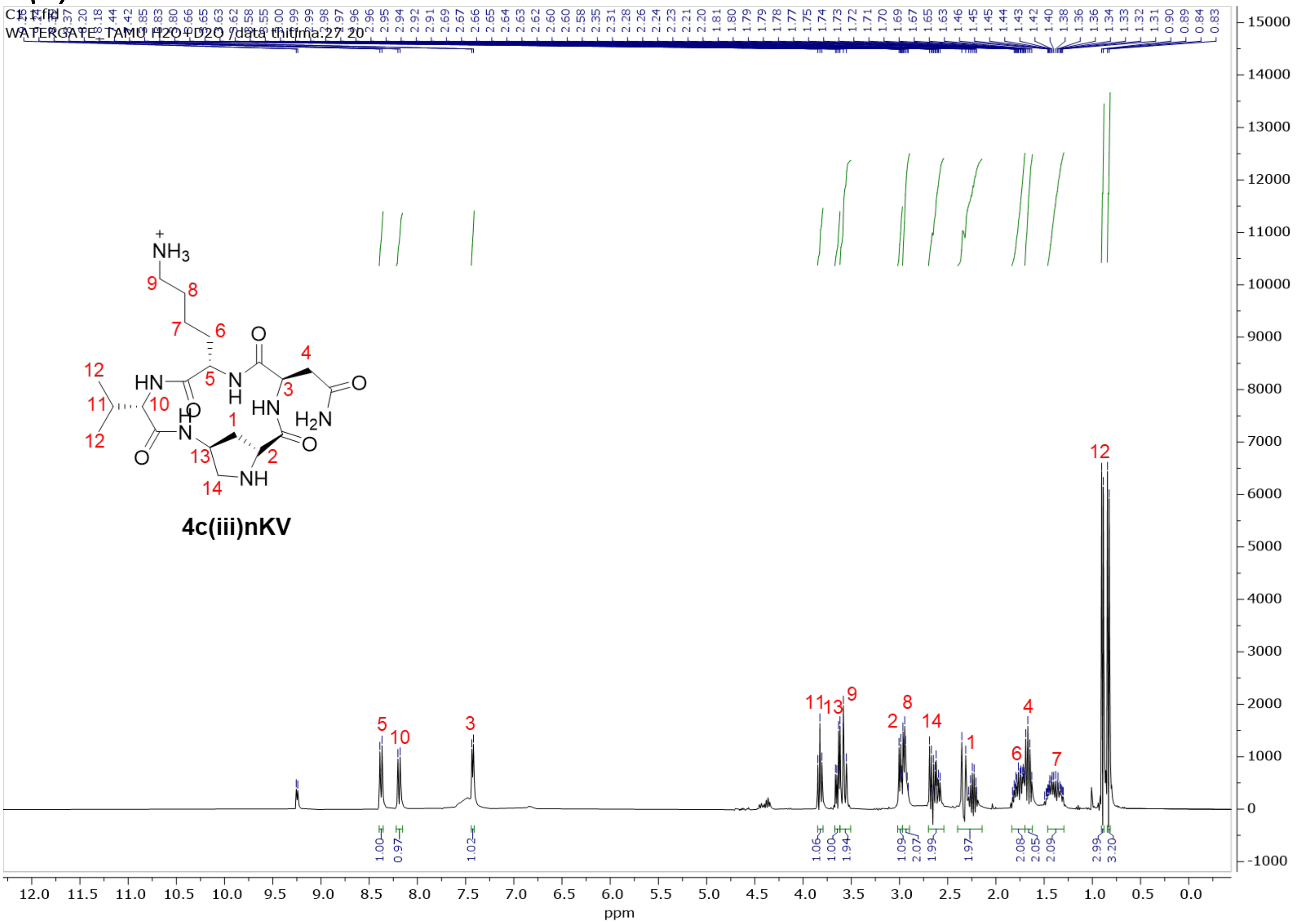

^{1}H-NMR of 4c(iii)nKV (400 MHz, 90%H_2O + 10% D_2O)

^{1}H NMR (400 MHz, H_2O+D_2O) δ 8.38 (d, J = 9.0 Hz, 1H), 8.19 (d, J = 9.0 Hz, 1H), 7.43 (d, J = 6.7 Hz, 1H), 3.83 (t, J = 8.5 Hz, 1H), 3.64 (dd, J = 13.0, 5.9 Hz, 1H), 3.57 (d, J = 12.8 Hz, 1H), 2.99 (dd, J = 6.8, 4.2 Hz, 1H), 2.95 (dt, J = 10.2, 4.7 Hz, 2H), 2.75 – 2.56 (m, 2H), 2.38 – 2.17 (m, 2H), 1.84 – 1.70 (m, 2H), 1.70 – 1.62 (m, 2H), 1.52 – 1.26 (m, 2H), 0.89 (d, J = 7.1 Hz, 3H), 0.83 (d, J = 7.3 Hz, 4H). High resolution ESI^{+} : m/z calcd for $[C_{20}H_{36}N_7O_5]^{+}$ 454.2772 found 454.2770.

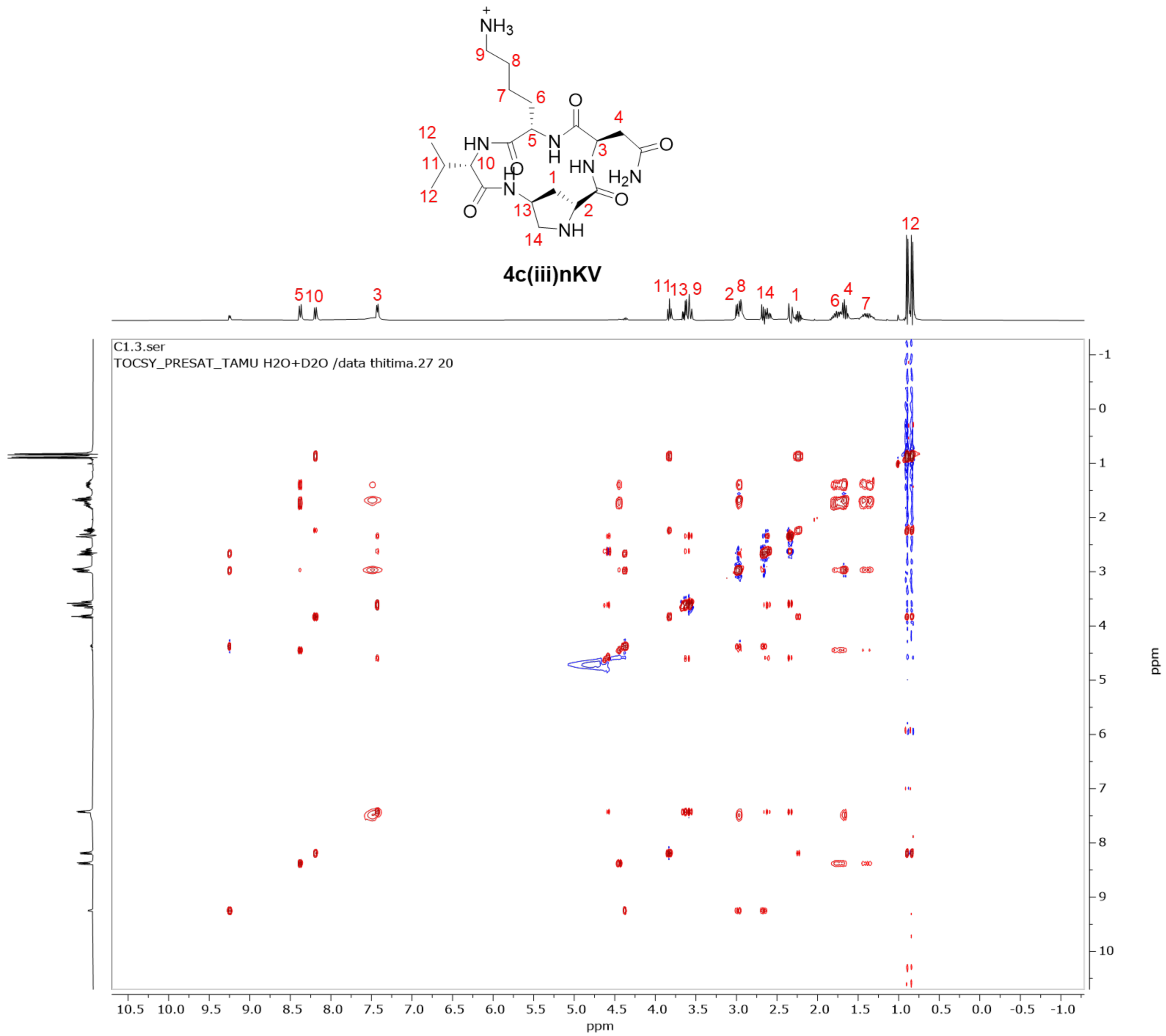

TOCSY of 4c(iii)nKV (90% H_2O + 10% D_2O)

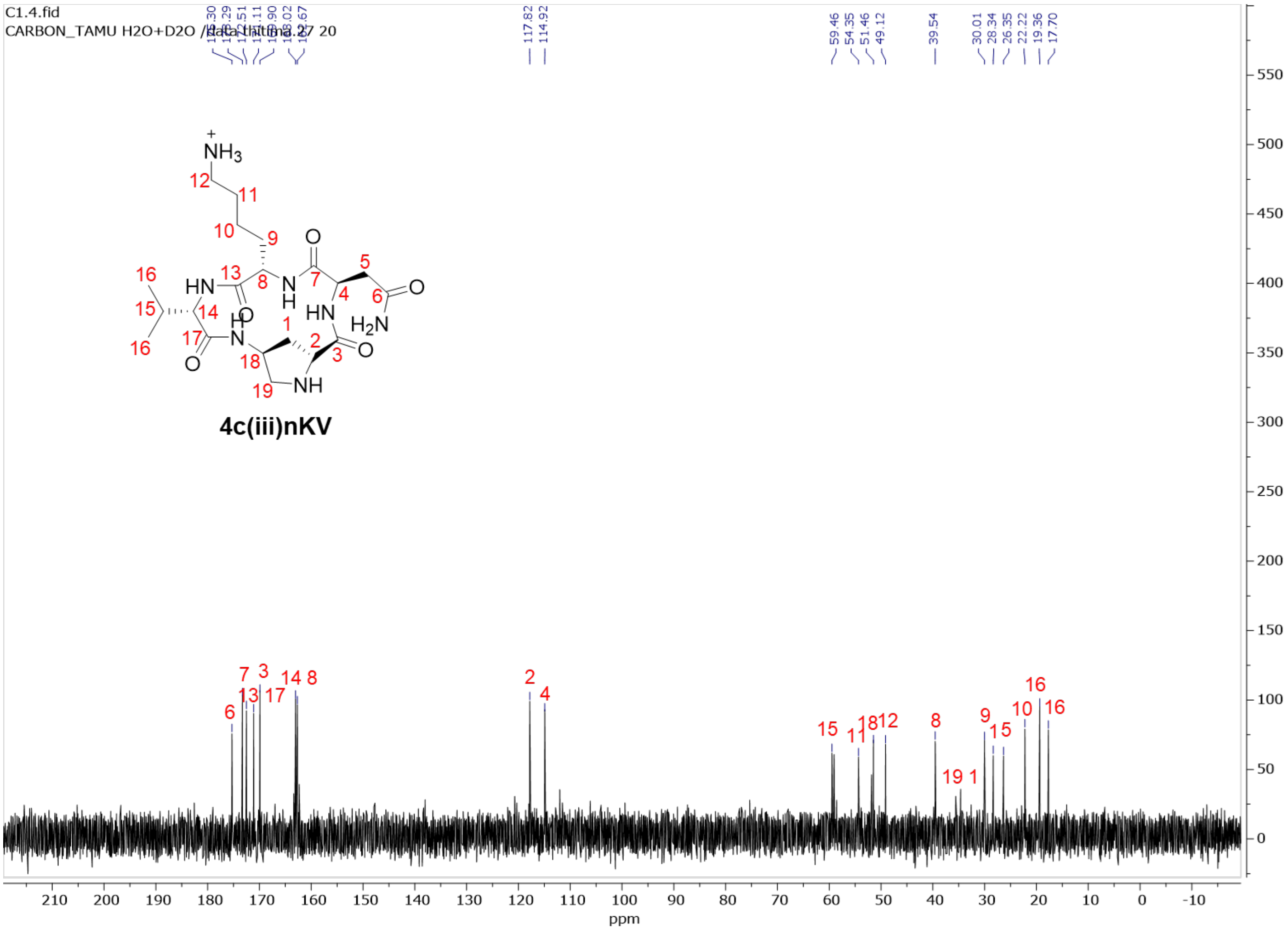

^{13}C-NMR of 4c(iii)nKV (100 MHz, 90%H_2O + 10% D_2O)

^{13}C NMR (101 MHz, 90%H_2O + 10%D_2O) δ 175.30, 173.29, 172.51, 171.11, 169.90, 163.02, 162.67, 117.82, 114.92, 59.46, 54.35, 51.46, 49.12, 39.54, 30.01, 28.34, 26.35, 22.22, 19.36, 17.70.

Characterization (HPLC and HRMS) for Series 3 and 4 Compounds

3a(i)

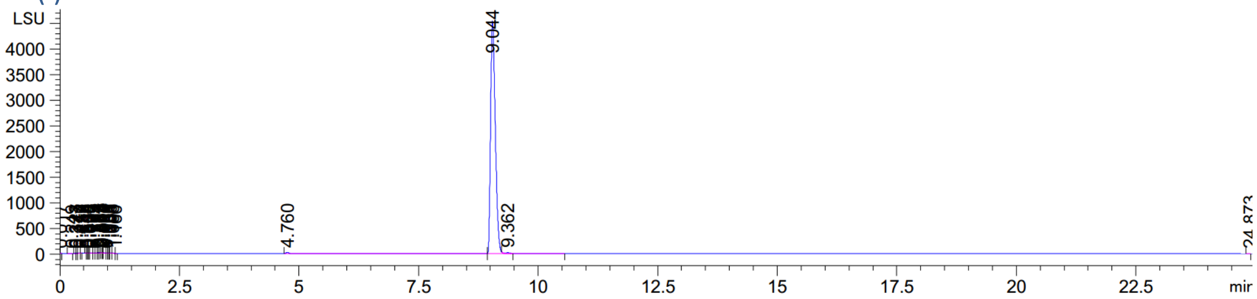

220428-112122-1 #55-66 RT: 0.24-0.29 AV: 12 SB: 13 0.09-0.14 NL: 6
T: FTMS + p ESI Full ms [200.0000-1000.0000]

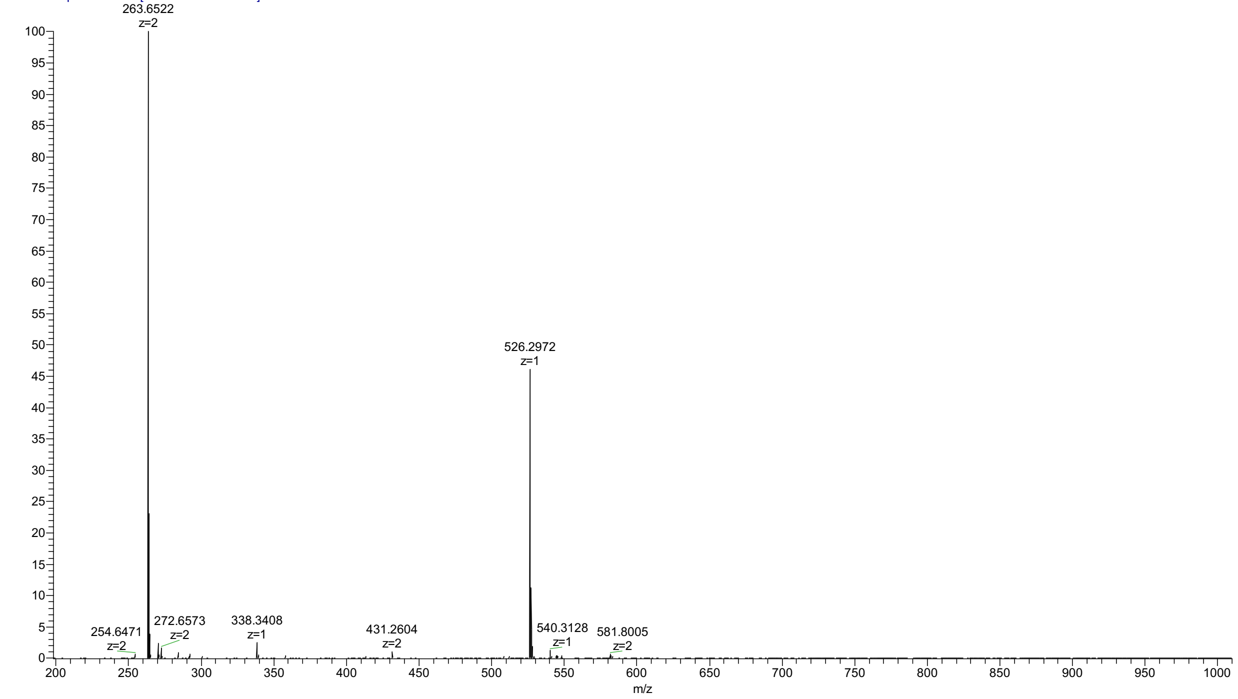

3a(ii)

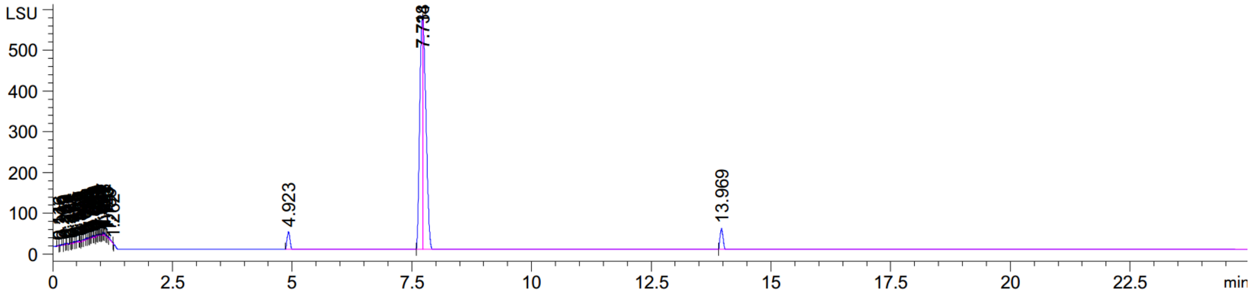
LSU
7.733
4.923
13.969
min

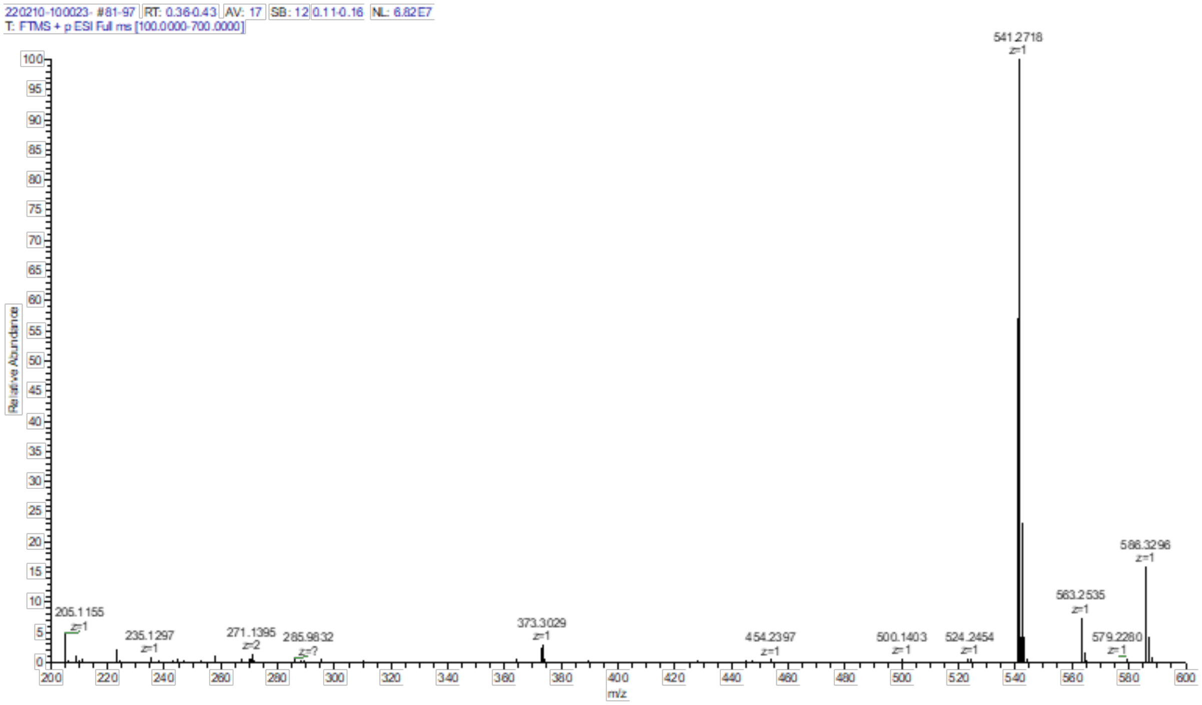
220210-100023- #81-97 RT: 0.36-0.43 AV: 17 SB: 12 0.11-0.16 NL: 6.82E7
T: FTMS + p ESI Full ms [100.0000-700.0000]
541.2718 z=1
586.3296 z=1
563.2535 z=1
579.2280 z=1
524.2454 z=1
500.1403 z=1
454.2397 z=1
373.3029 z=1
285.9832 z=?
271.1395 z=2
235.1297 z=1
205.1155 z=1
Relative Abundance
m/z

3a(iii)

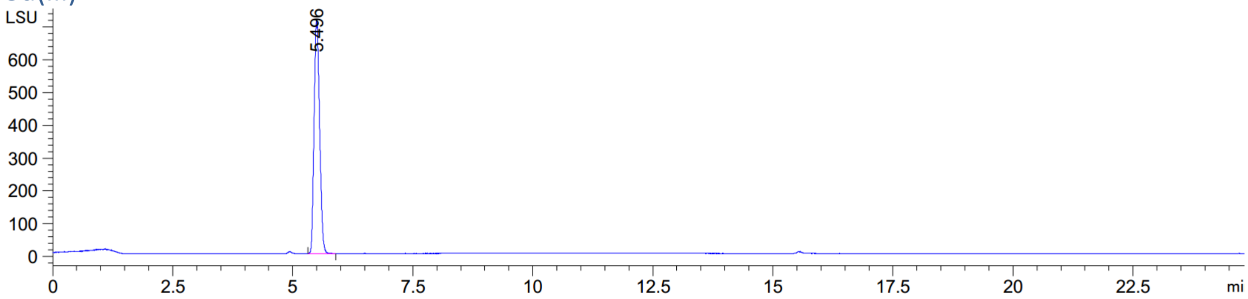
LSU
5.496
600
500
400
300
200
100
0
0
2.5
5
7.5
10
12.5
15
17.5
20
22.5
mi

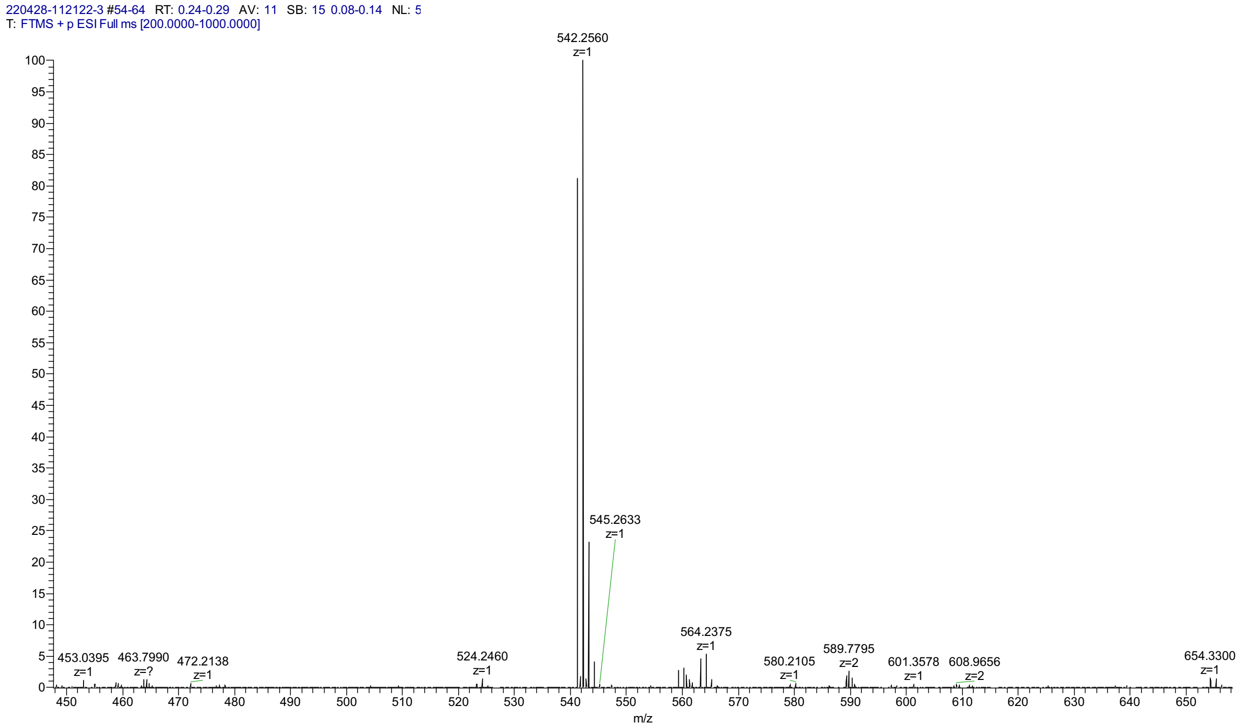
220428-112122-3 #54-64 RT: 0.24-0.29 AV: 11 SB: 15 0.08-0.14 NL: 5
T: FTMS + p ESI Full ms [200.0000-1000.0000]
542.2560
z=1
545.2633
z=1
564.2375
z=1
453.0395
z=1
463.7990
z=?
472.2138
z=1
524.2460
z=1
580.2105
z=1
589.7795
z=2
601.3578
z=1
608.9656
z=2
654.3300
z=1
m/z

3a(iii)mouse

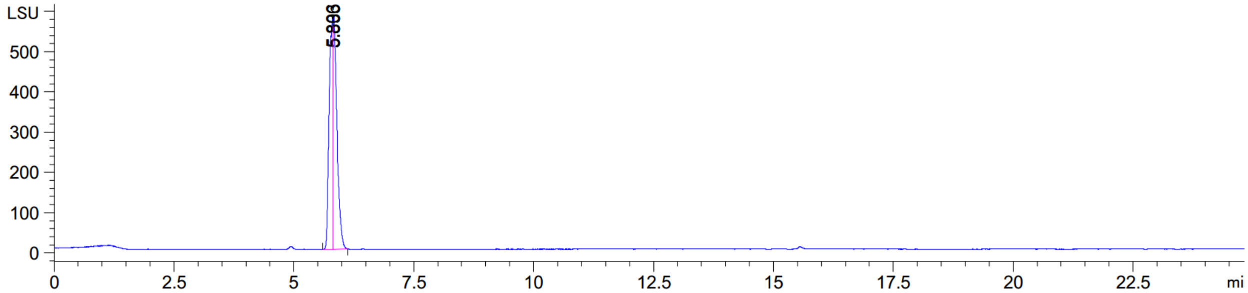

220428-112122-8 #57-66 RT: 0.25-0.29 AV: 10 SB: 12 0.08-0.13 NL: 5
T: FTMS + p ESI Full ms [200.0000-1000.0000]

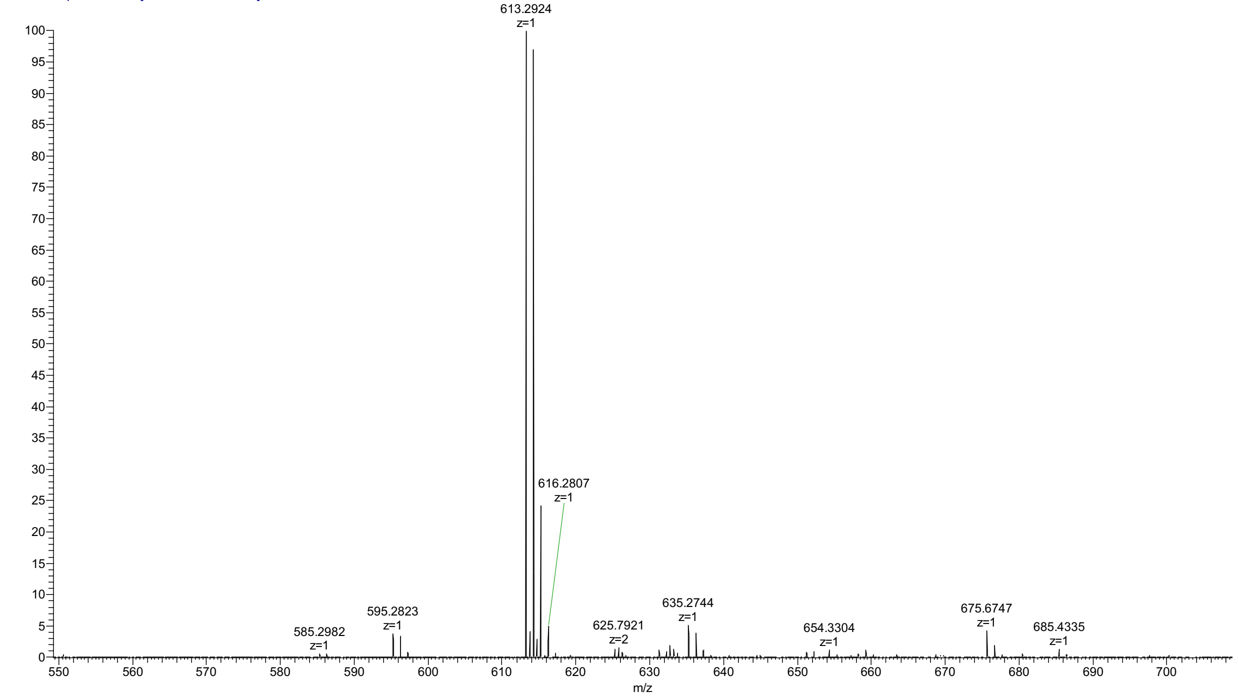

3b(i)

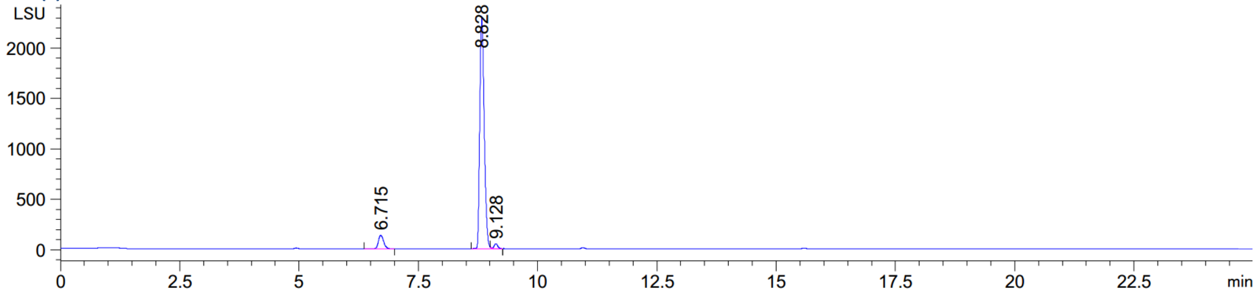
LSU
2000
1500
1000
500
0
8.828
6.715
9.128
0
2.5
5
7.5
10
12.5
15
17.5
20
22.5
min

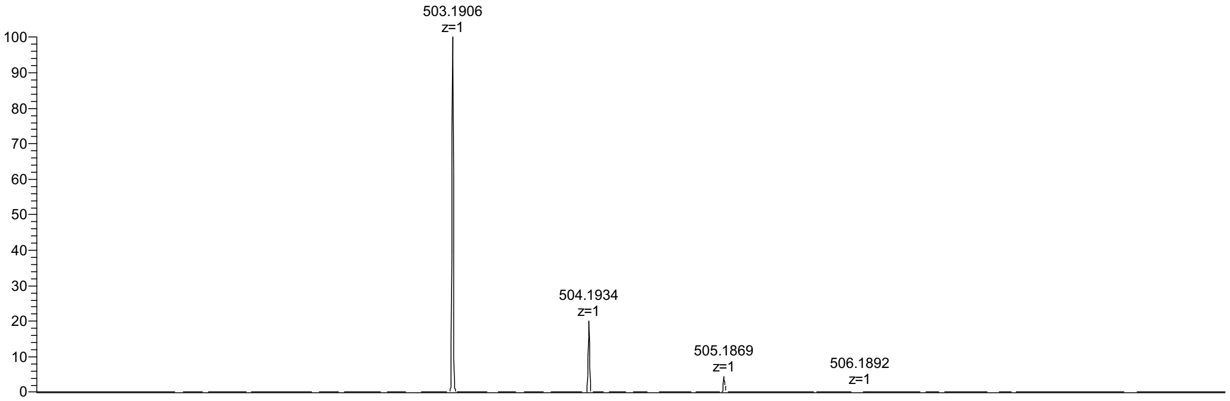
503.1906
z=1
504.1934
z=1
505.1869
z=1
506.1892
z=1
100
90
80
70
60
50
40
30
20
10
0

3b(ii)

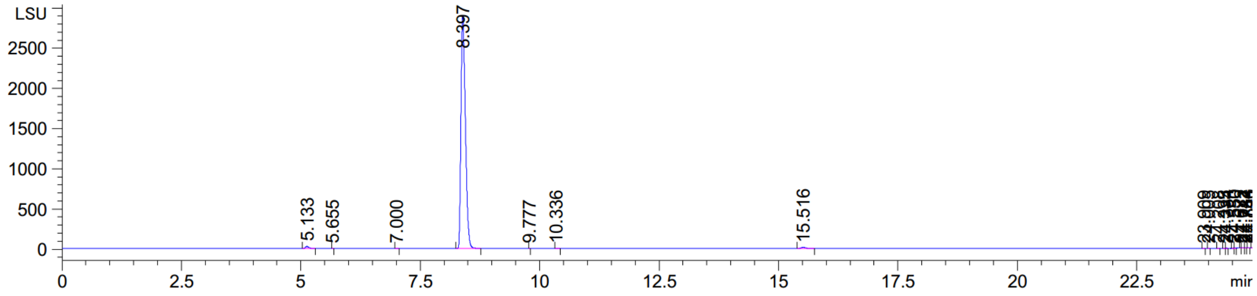
LSU
8.397
5.133
5.655
7.000
9.777
10.336
15.516
min

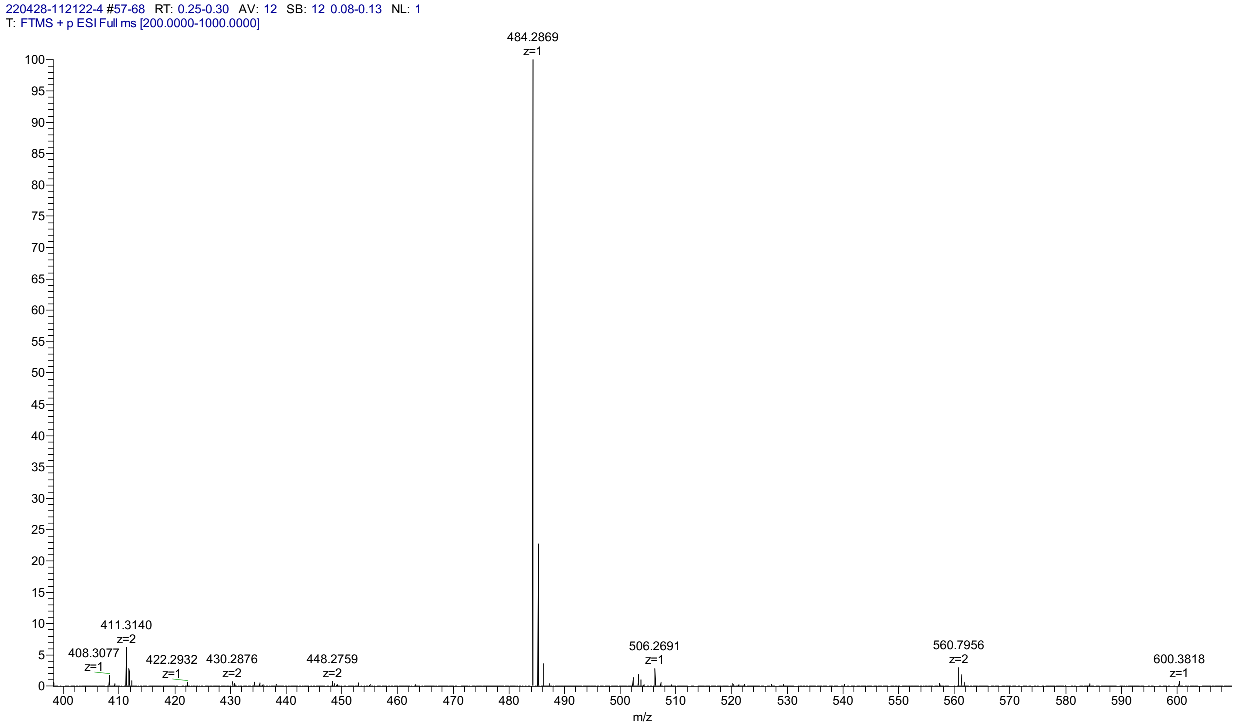
220428-112122-4 #57-68 RT: 0.25-0.30 AV: 12 SB: 12 0.08-0.13 NL: 1
T: FTMS + p ESI Full ms [200.0000-1000.0000]
484.2869
z=1
411.3140
z=2
408.3077
z=1
422.2932
z=1
430.2876
z=2
448.2759
z=2
506.2691
z=1
560.7956
z=2
600.3818
z=1
m/z

3b(iii)

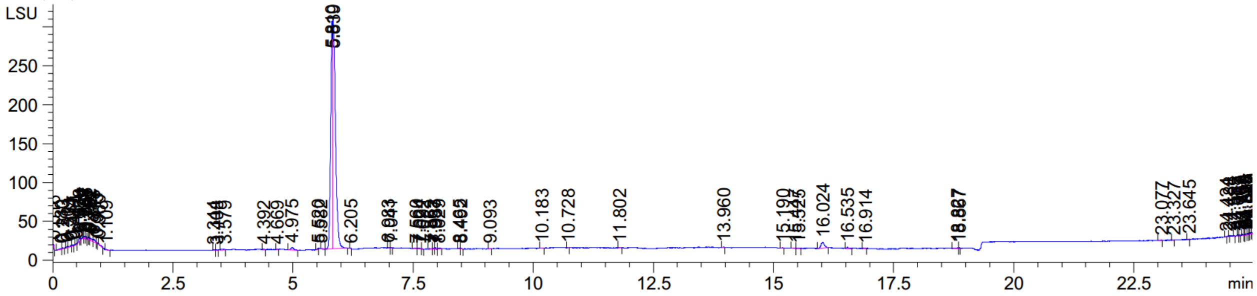
LSU
min
5.839
571.3184
z=1
571.8201
z=?
572.3216
z=1
573.3239
z=1
574.3265
z=1
567.3967
z=1

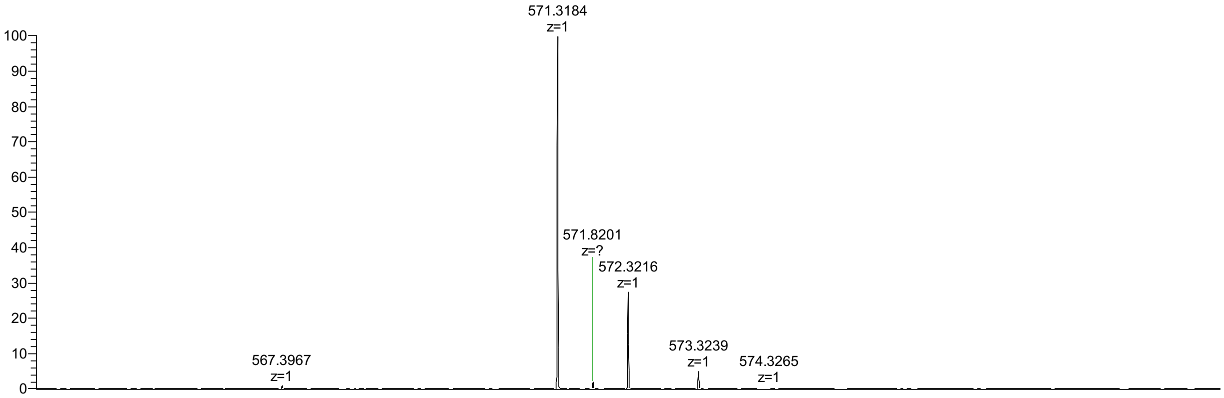
571.3184
z=1
571.8201
z=?
572.3216
z=1
573.3239
z=1
574.3265
z=1
567.3967
z=1

3c(i)

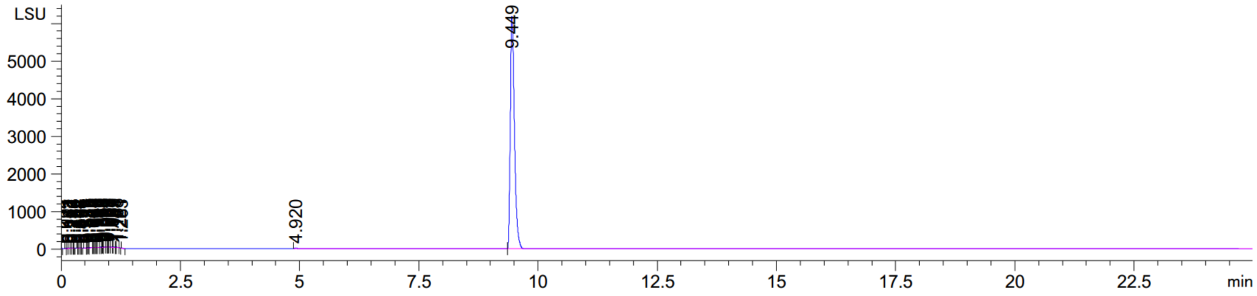
LSU
9.449
4.920
min

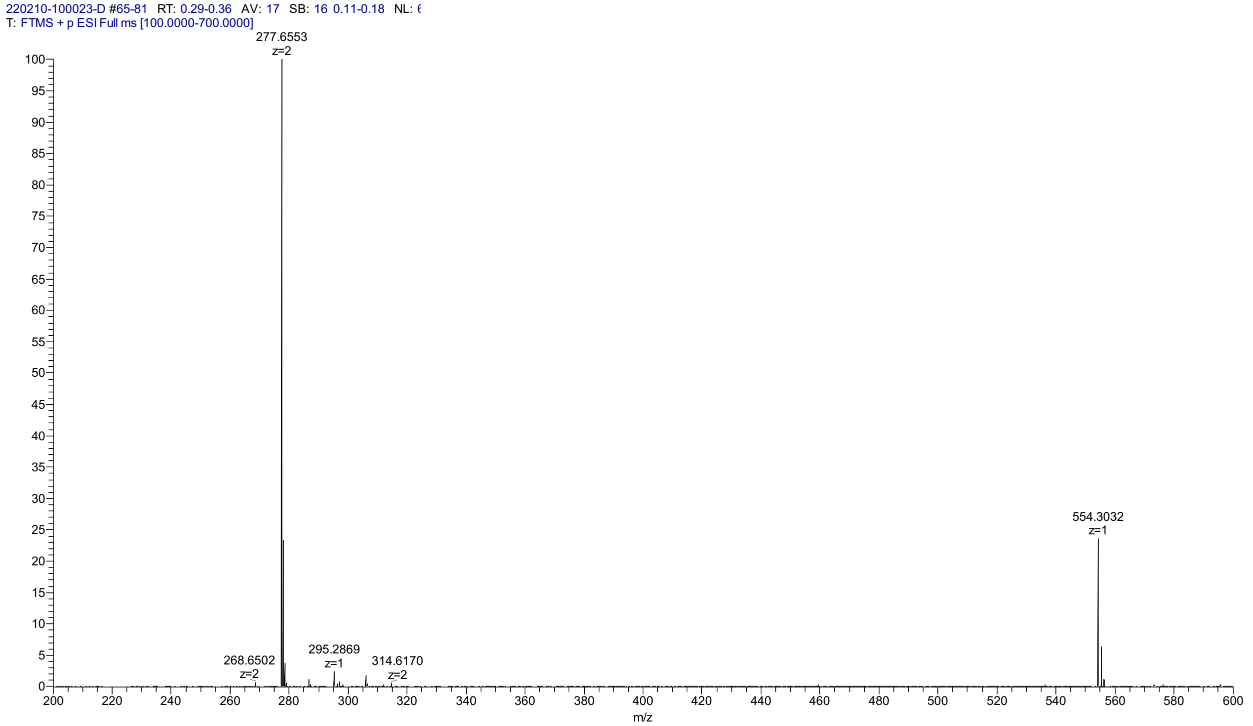
220210-100023-D #65-81 RT: 0.29-0.36 AV: 17 SB: 16 0.11-0.18 NL:
T: FTMS + p ESI Full ms [100.0000-700.0000]
277.6553
z=2
554.3032
z=1
268.6502
z=2
295.2869
z=1
314.6170
z=2
m/z

3c(ii)

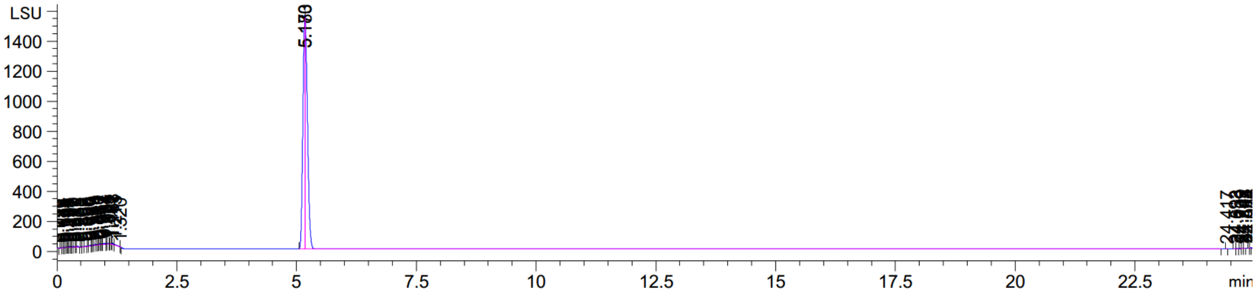

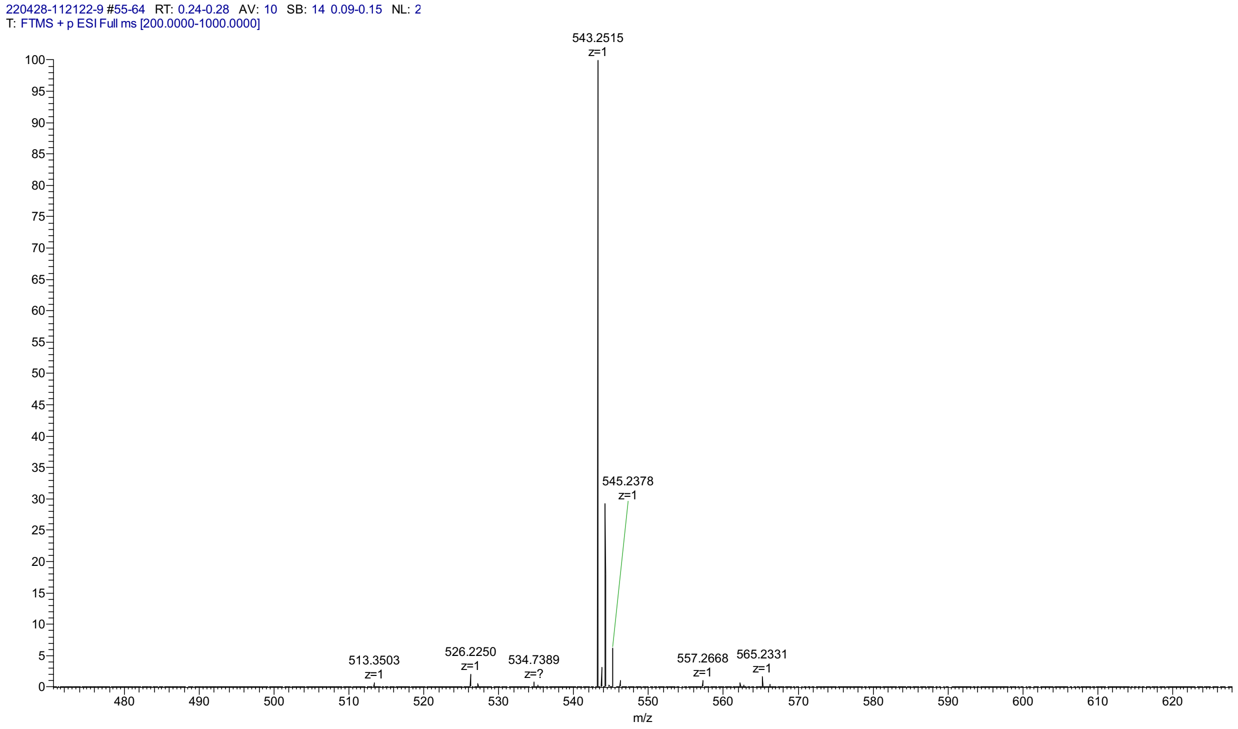
220428-112122-9 #55-64 RT: 0.24-0.28 AV: 10 SB: 14 0.09-0.15 NL: 2
T: FTMS + p ESI Full ms [200.0000-1000.0000]
543.2515
z=1
545.2378
z=1
513.3503
z=1
526.2250
z=1
534.7389
z=?
557.2668
z=1
565.2331
z=1
m/z

3c(ii)mouse

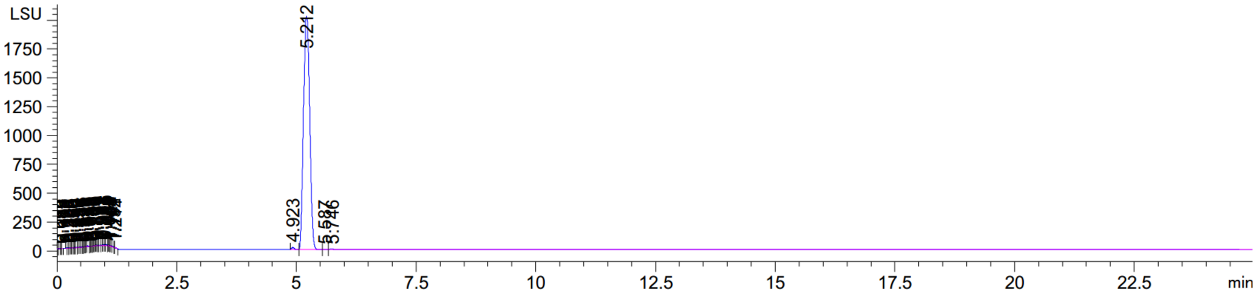
LSU
1750
1500
1250
1000
750
500
250
0
5.212
4.923
5.587
5.746
0
2.5
5
7.5
10
12.5
15
17.5
20
22.5
min

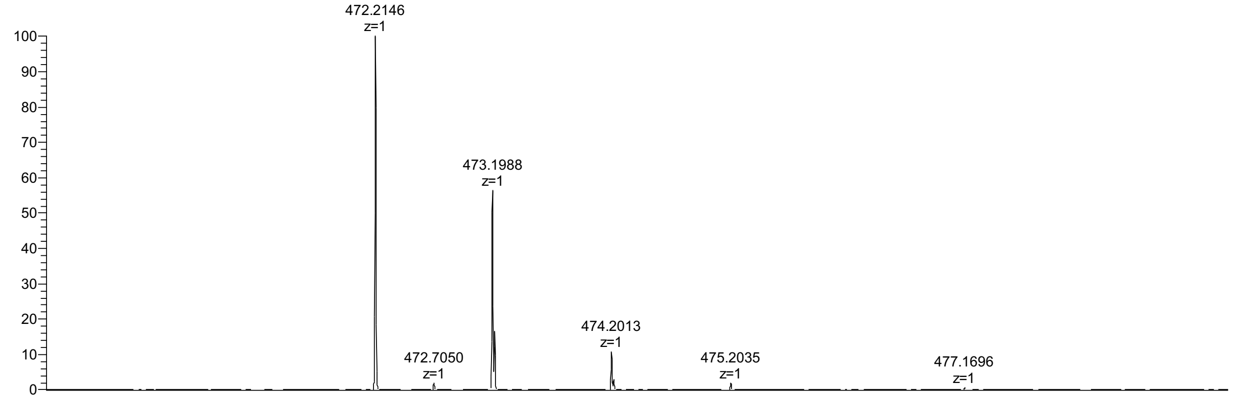
472.2146
z=1
472.7050
z=1
473.1988
z=1
474.2013
z=1
475.2035
z=1
477.1696
z=1
100
90
80
70
60
50
40
30
20
10
0

3c(iii)

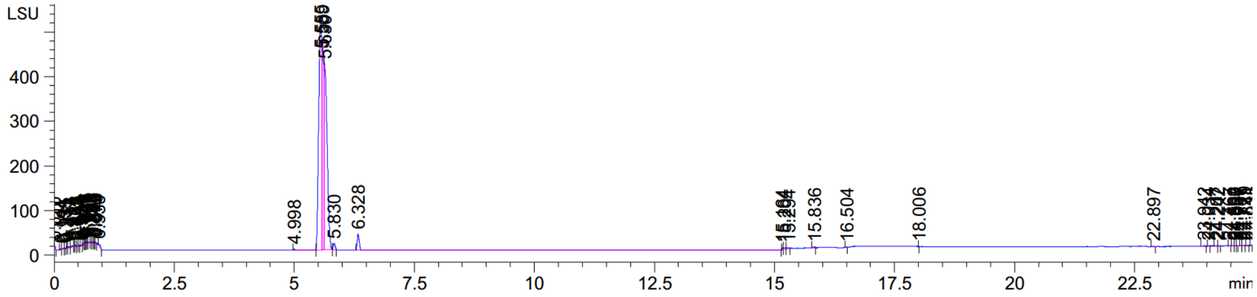
LSU
400
300
200
100
0
4.998
5.830
6.328
15.836
16.504
18.006
22.897
0
2.5
5
7.5
10
12.5
15
17.5
20
22.5
min

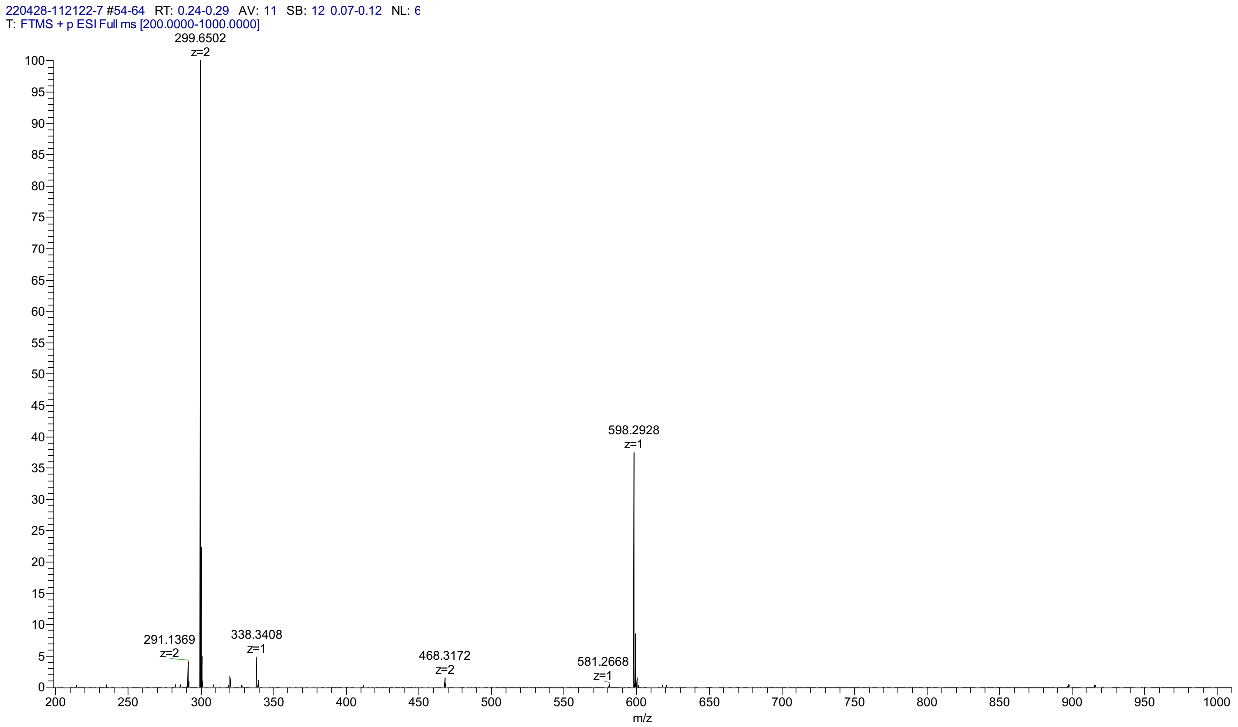
220428-112122-7 #54-64 RT: 0.24-0.29 AV: 11 SB: 12 0.07-0.12 NL: 6
T: FTMS + p ESI Full ms [200.0000-1000.0000]
299.6502
z=2
598.2928
z=1
291.1369
z=2
338.3408
z=1
468.3172
z=2
581.2668
z=1
m/z

4a(ii)INS

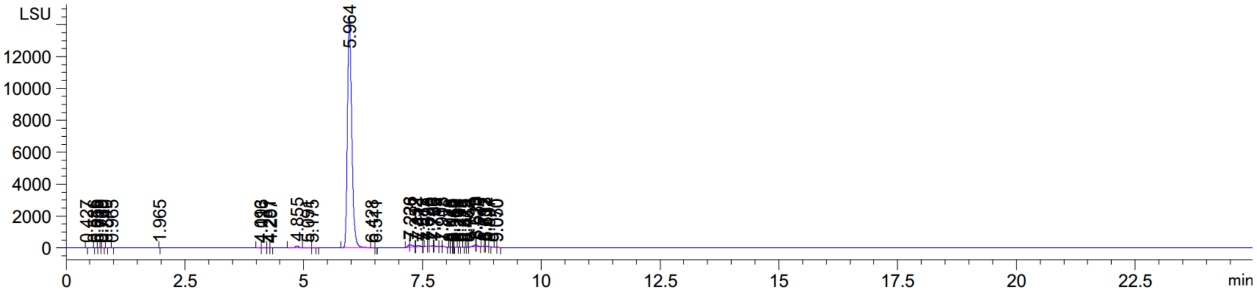

LSU
5.964
1.965
4.855
min

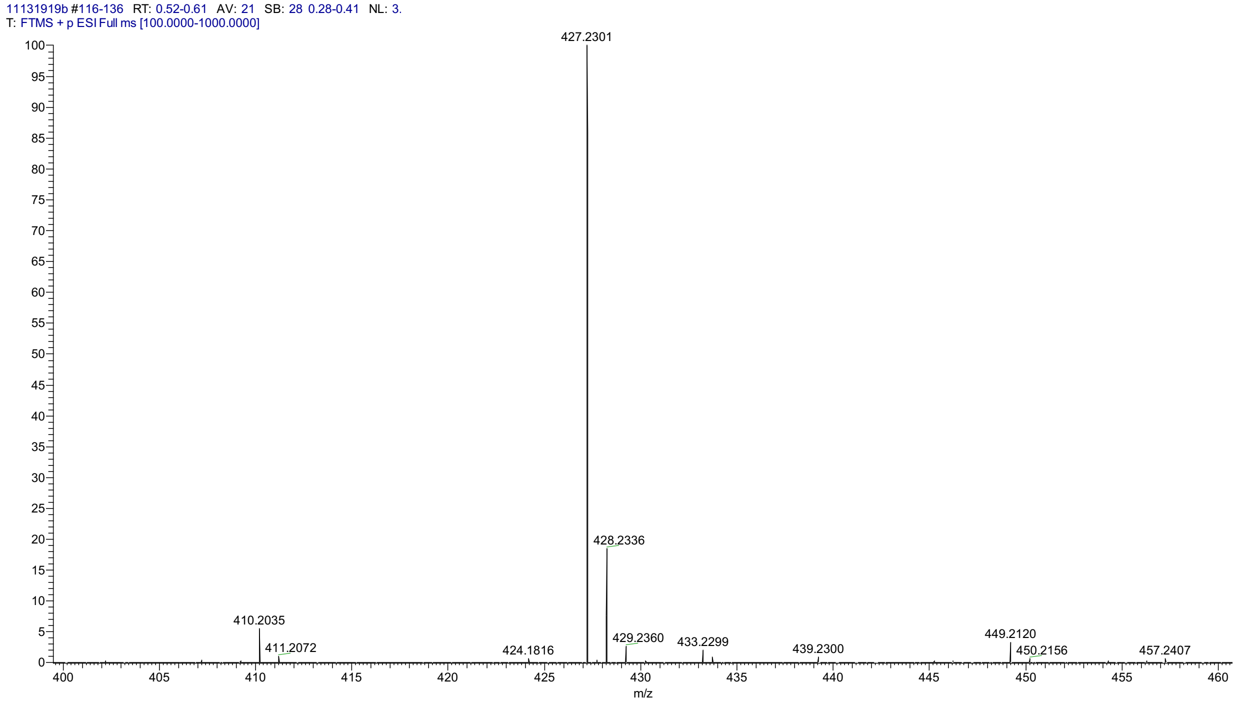

11131919b #116-136 RT: 0.52-0.61 AV: 21 SB: 28 0.28-0.41 NL: 3.
T: FTMS + p ESI Full ms [100.0000-1000.0000]
427.2301
428.2336
410.2035
411.2072
424.1816
429.2360
433.2299
439.2300
449.2120
450.2156
457.2407
m/z

4a(ii)snv

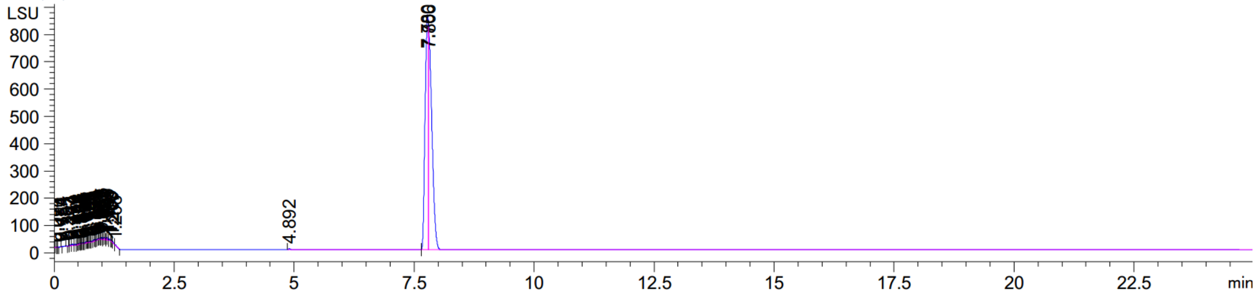
LSU
800
700
600
500
400
300
200
100
0
4.892
0
2.5
5
7.5
10
12.5
15
17.5
20
22.5
min

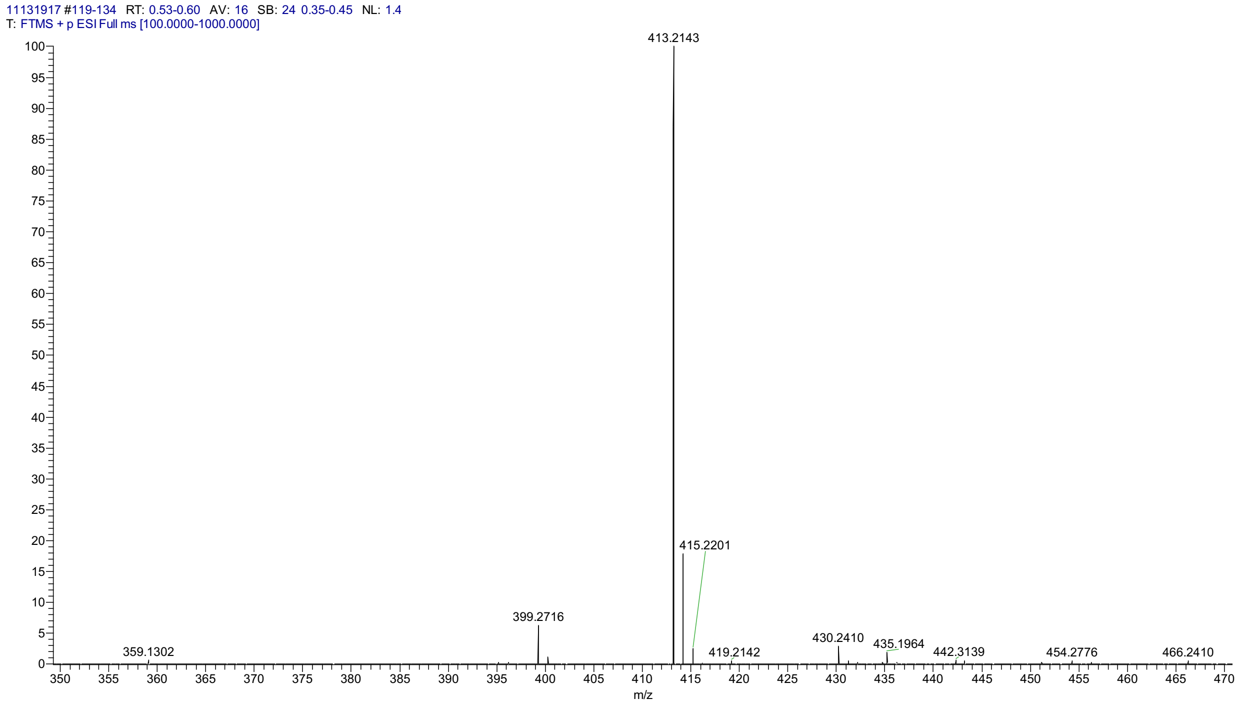
11131917 #119-134 RT: 0.53-0.60 AV: 16 SB: 24 0.35-0.45 NL: 1.4
T: FTMS + p ESI Full ms [100.0000-1000.0000]
413.2143
415.2201
399.2716
359.1302
419.2142
430.2410
435.1964
442.3139
454.2776
466.2410
m/z

4a(ii)Vsn

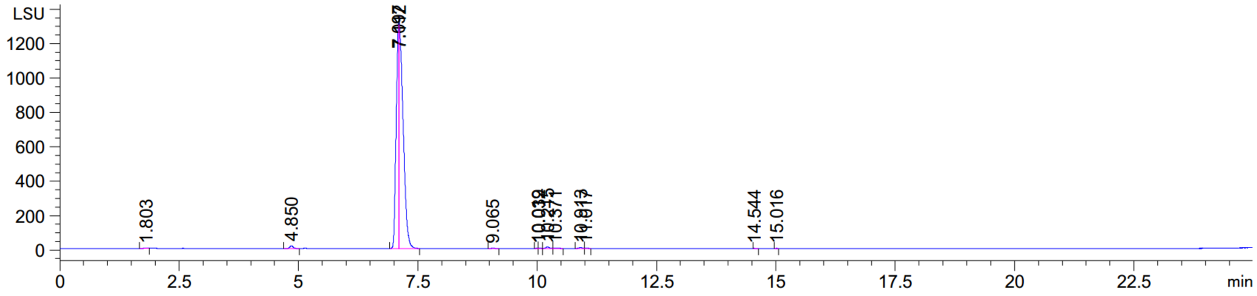

11131912a #103-139 RT: 0.46-0.62 AV: 37 SB: 24 0.22-0.32 NL: 2.
T: FTMS + p ESI Full ms [100.0000-1000.0000]

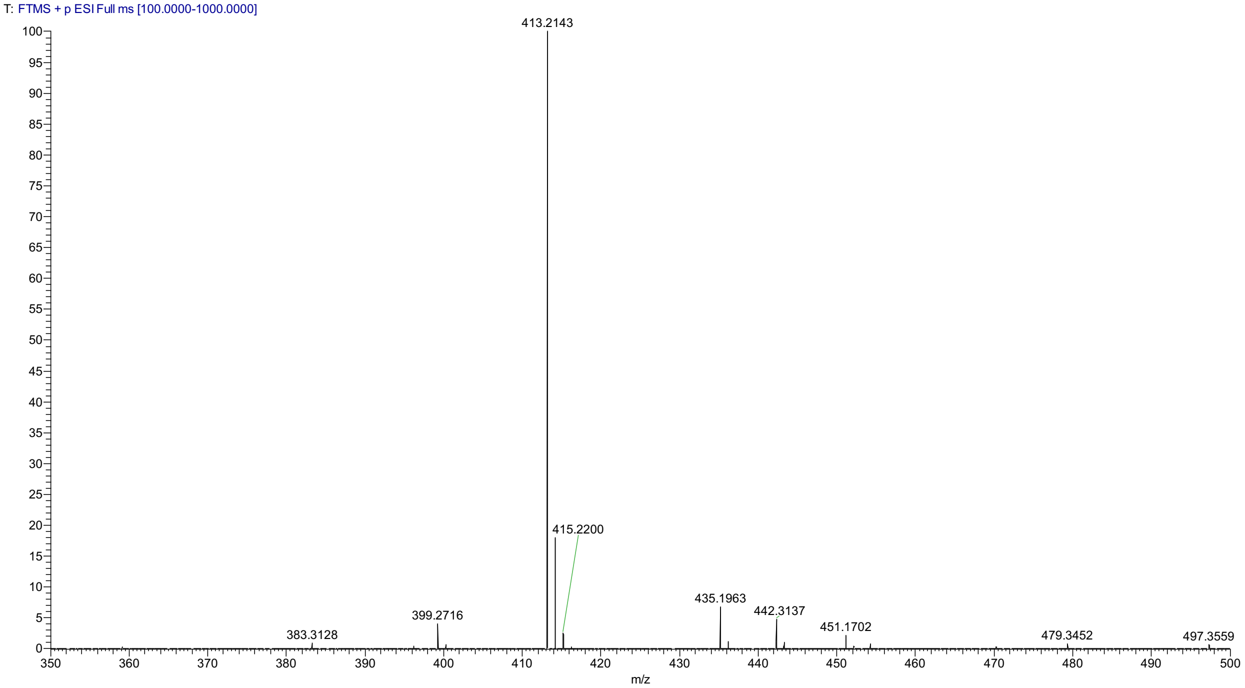

4b(iii)DSK

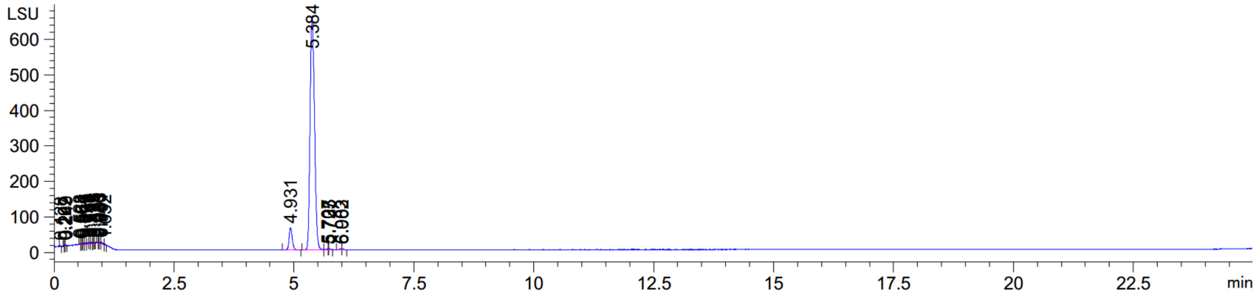
LSU
5.384
4.931
min

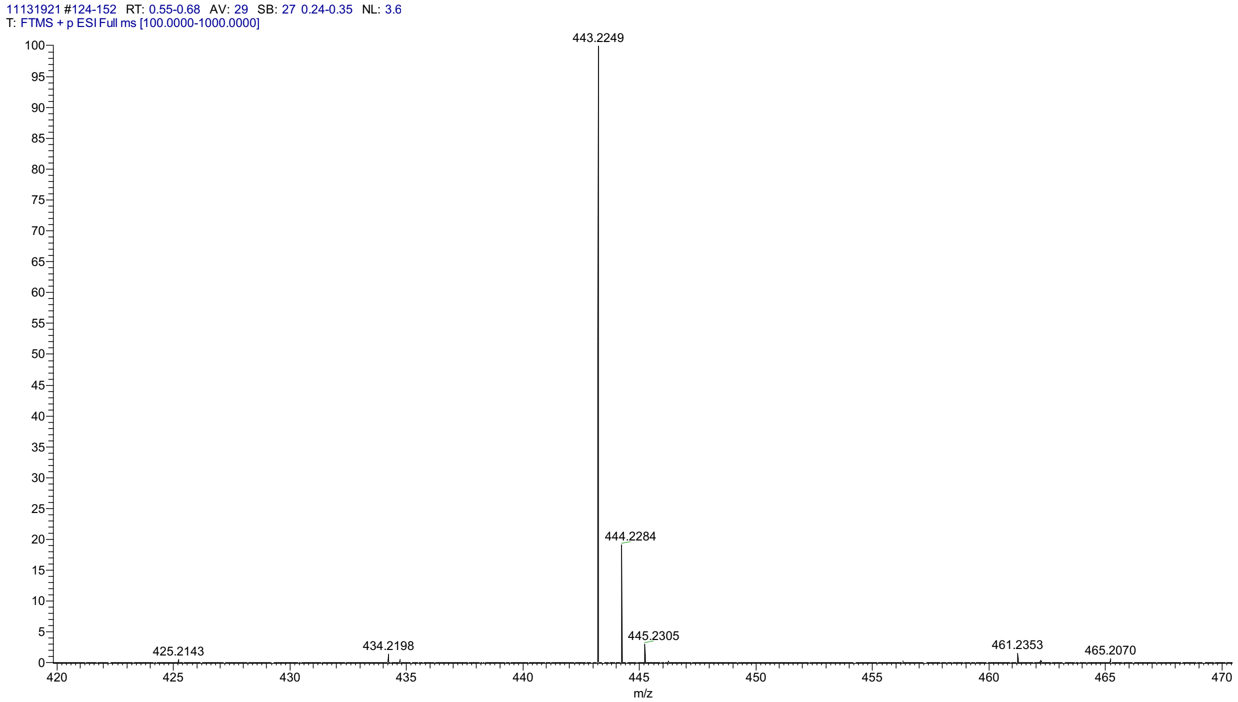
11131921 #124-152 RT: 0.55-0.68 AV: 29 SB: 27 0.24-0.35 NL: 3.6
T: FTMS + p ESI Full ms [100.0000-1000.0000]
443.2249
444.2284
445.2305
425.2143
434.2198
461.2353
465.2070
m/z

4b(iii)SKk

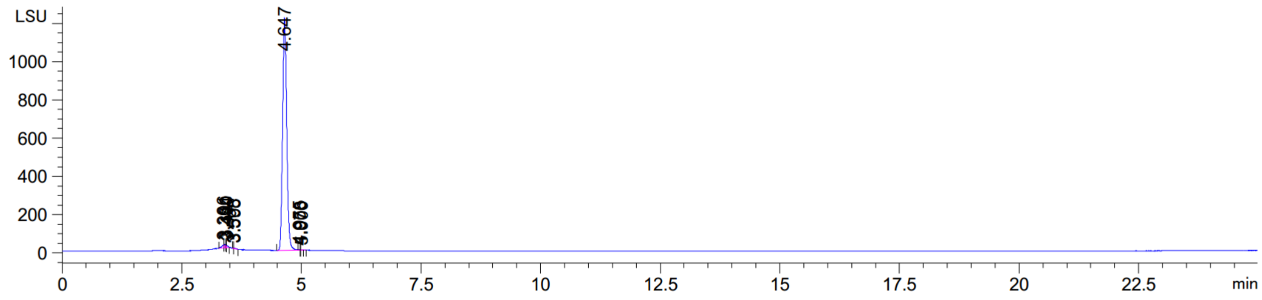
LSU
1000
800
600
400
200
0
4.647
0
2.5
5
7.5
10
12.5
15
17.5
20
22.5
min

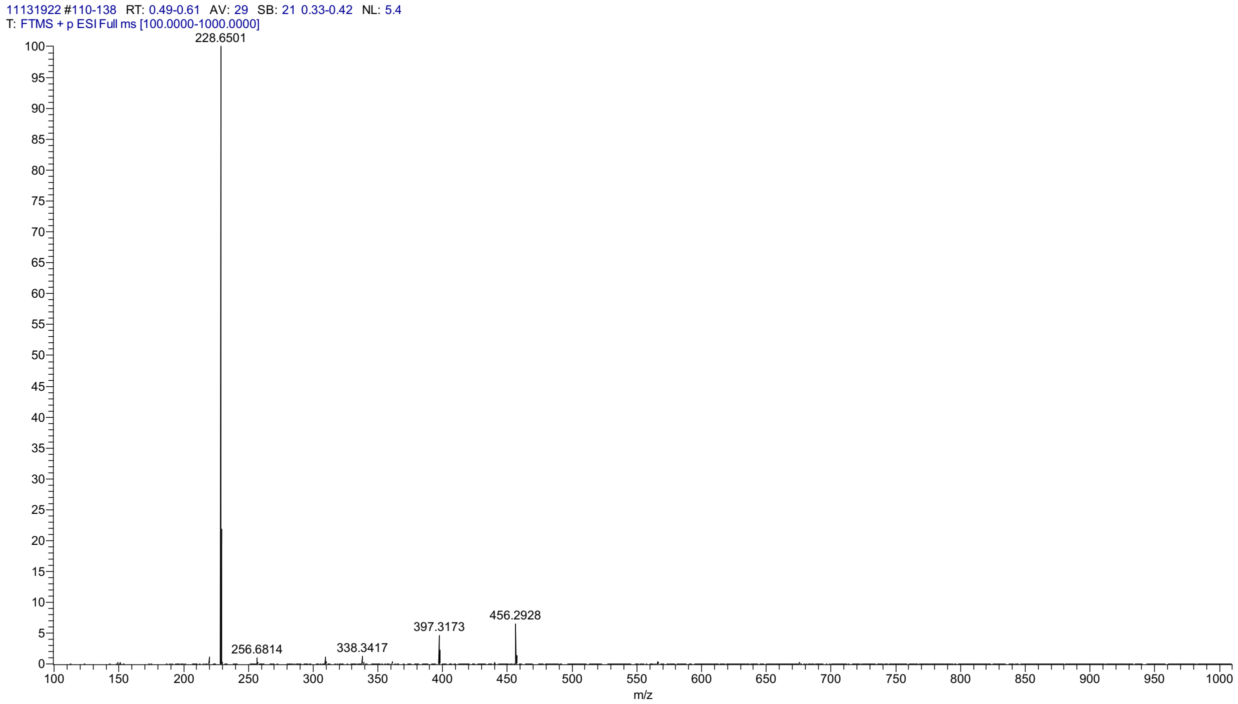
11131922 #110-138 RT: 0.49-0.61 AV: 29 SB: 21 0.33-0.42 NL: 5.4
T: FTMS + p ESI Full ms [100.0000-1000.0000]
228.6501
256.6814
338.3417
397.3173
456.2928
m/z

4b(iii)sKk

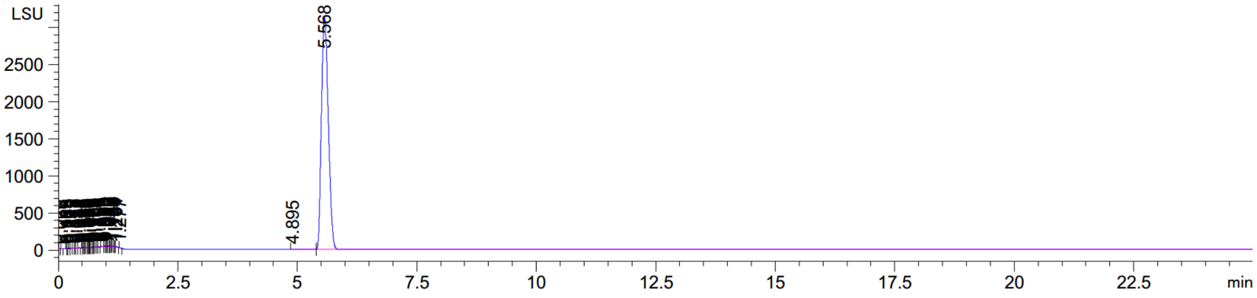
LSU
2500
2000
1500
1000
500
0
5.568
4.895
0
2.5
5
7.5
10
12.5
15
17.5
20
22.5
min

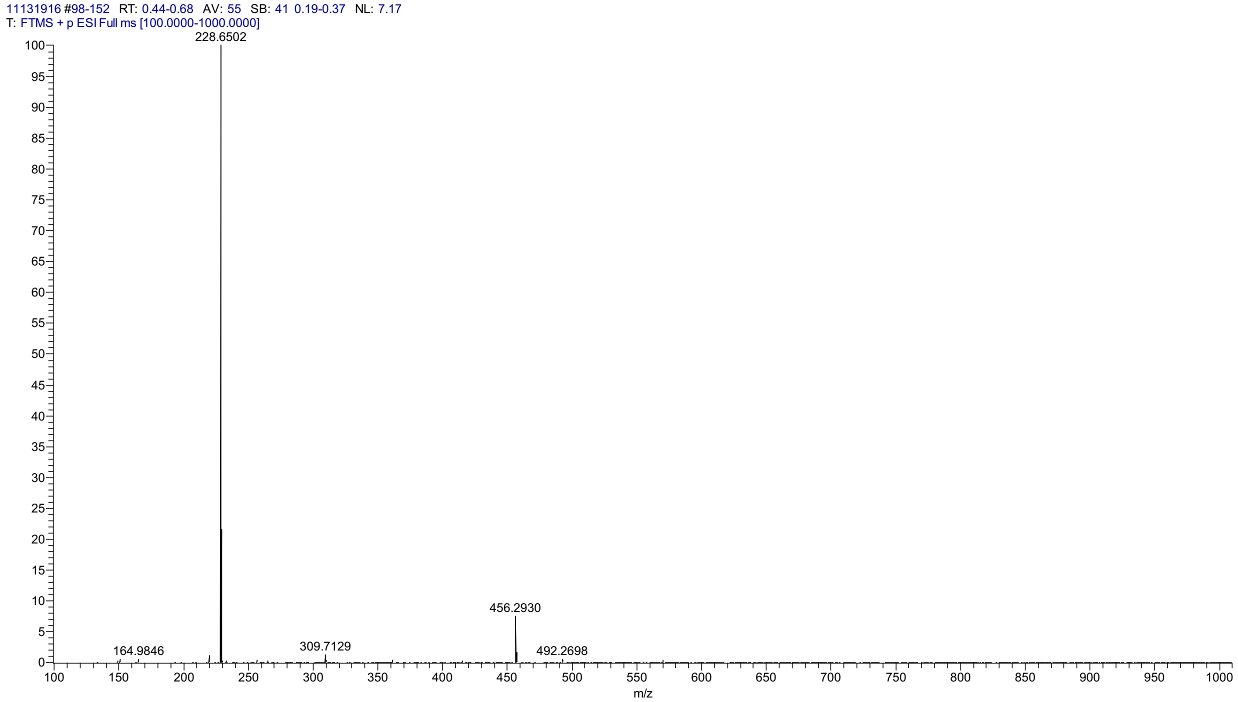
11131916 #98-152 RT: 0.44-0.68 AV: 55 SB: 41 0.19-0.37 NL: 7.17
T: FTMS + p ESI Full ms [100.0000-1000.0000]
228.6502
164.9846
309.7129
456.2930
492.2698
m/z

4b(iii)Kks

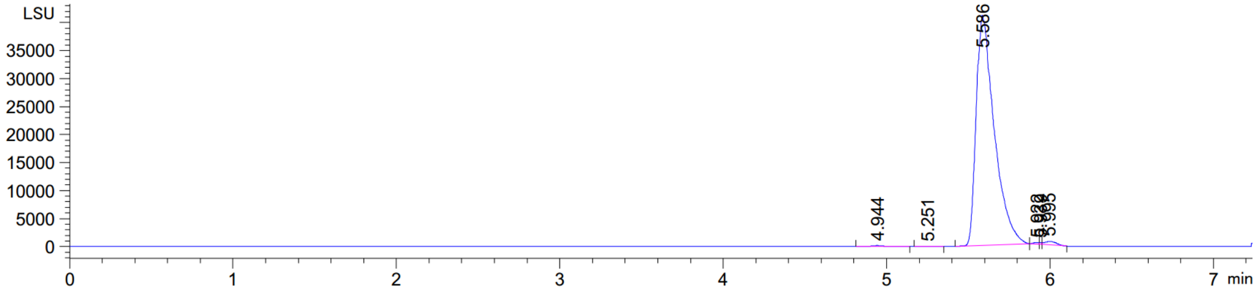
LSU
35000
30000
25000
20000
15000
10000
5000
0
4.944
5.251
5.586
5.995
0
1
2
3
4
5
6
7 min

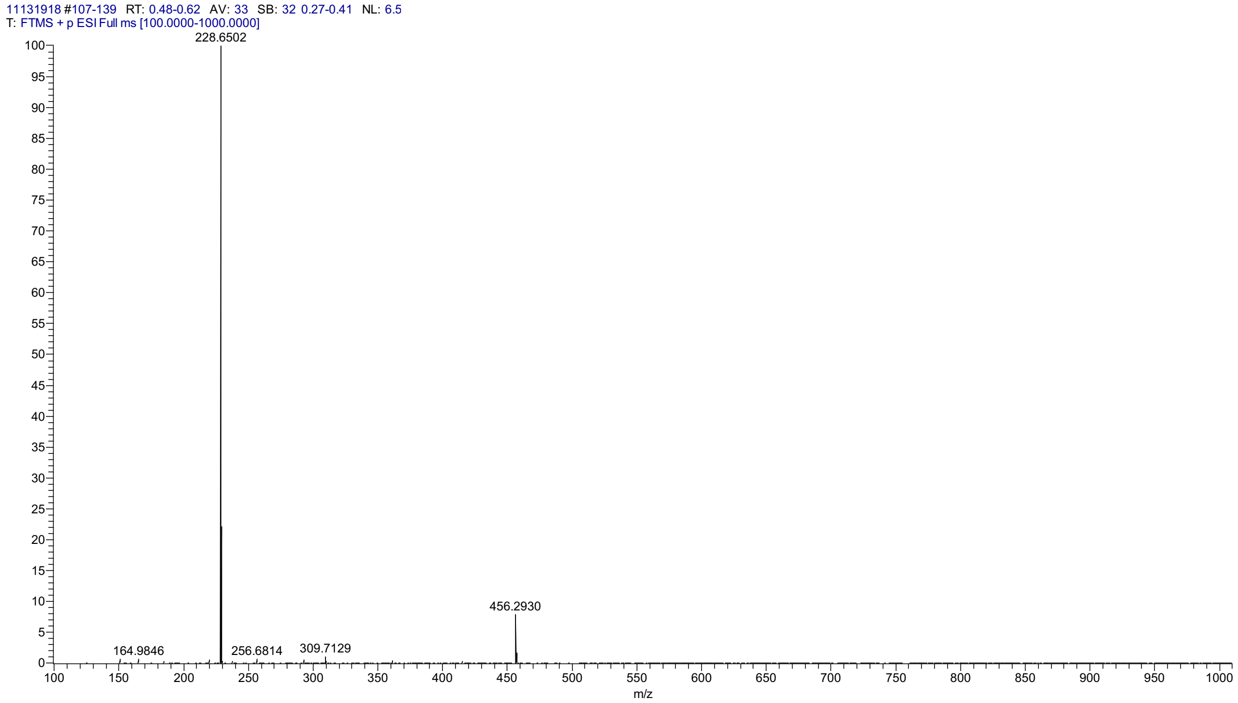
11131918 #107-139 RT: 0.48-0.62 AV: 33 SB: 32 0.27-0.41 NL: 6.5
T: FTMS + p ESI Full ms [100.0000-1000.0000]
228.6502
164.9846
256.6814
309.7129
456.2930
m/z

4c(iii)ENK

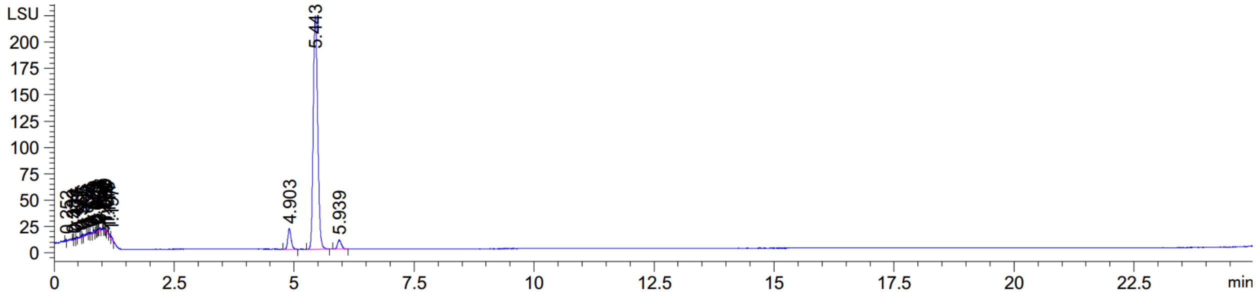

LSU
5.443
4.903
5.939
min

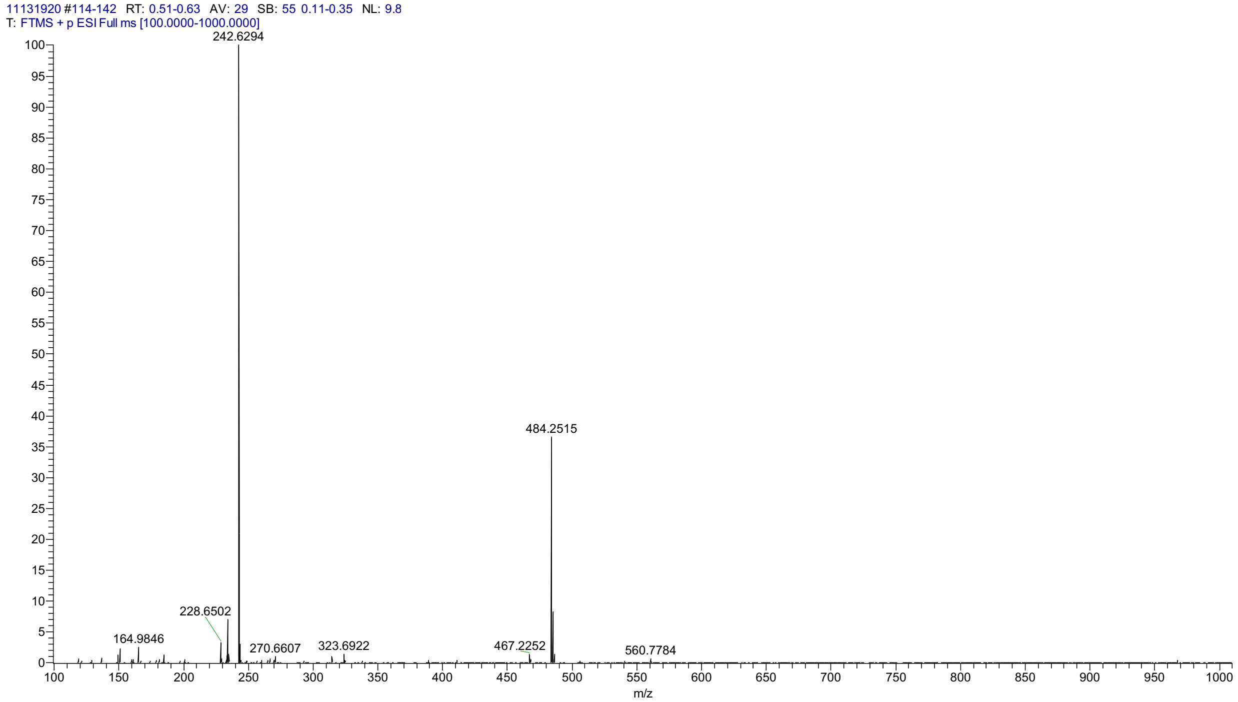

11131920 #114-142 RT: 0.51-0.63 AV: 29 SB: 55 0.11-0.35 NL: 9.8
T: FTMS + p ESI Full ms [100.0000-1000.0000]
242.6294
484.2515
228.6502
164.9846
270.6607
323.6922
467.2252
560.7784
m/z

4c(iii)nKV

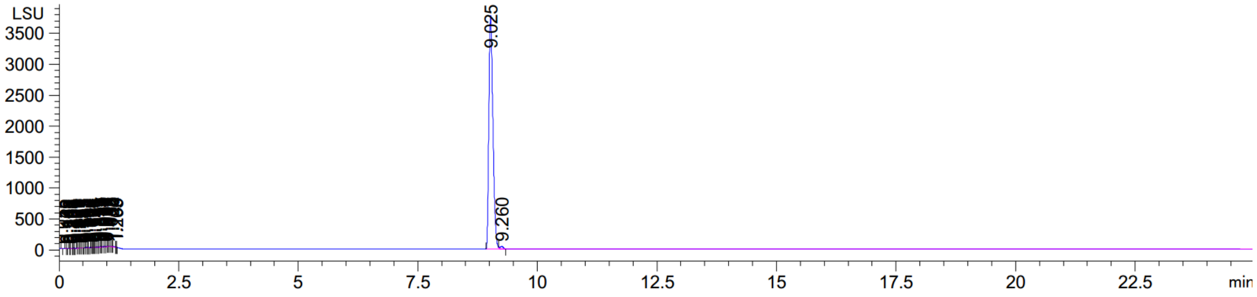

11131914 #113-173 RT: 0.50-0.77 AV: 61 SB: 65 0.15-0.44 NL: 3.1
T: FTMS + p ESI Full ms [100.0000-1000.0000]

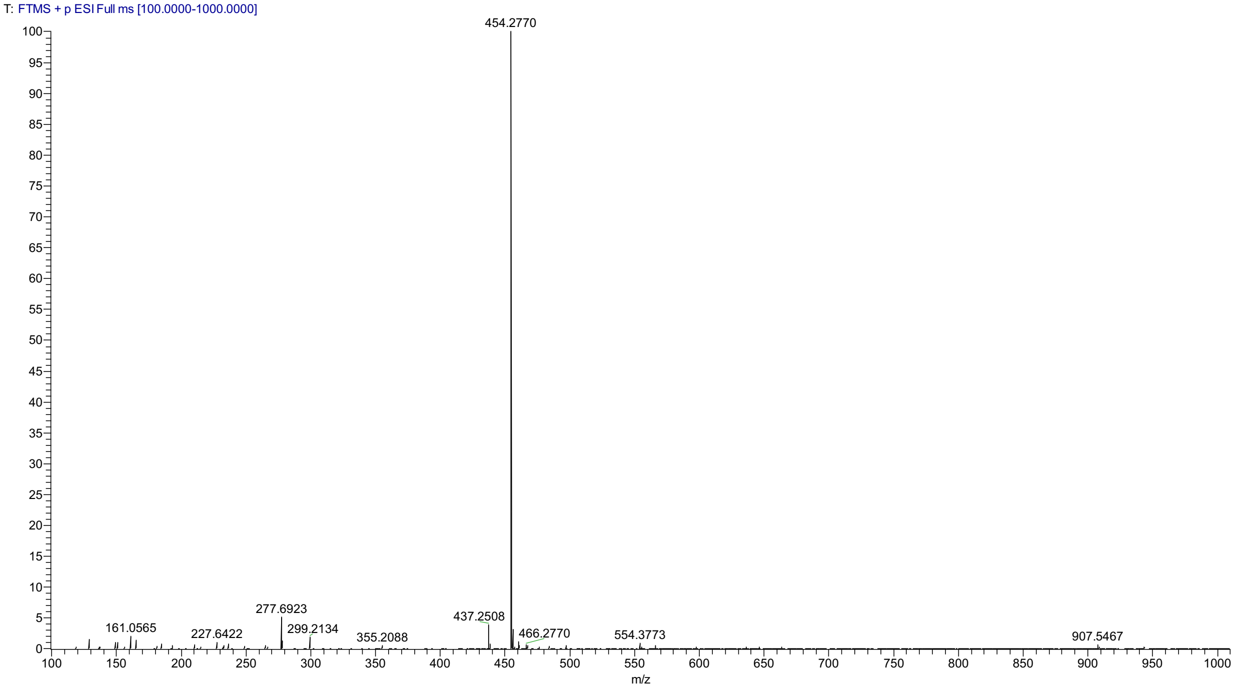

4c(iii)vkN

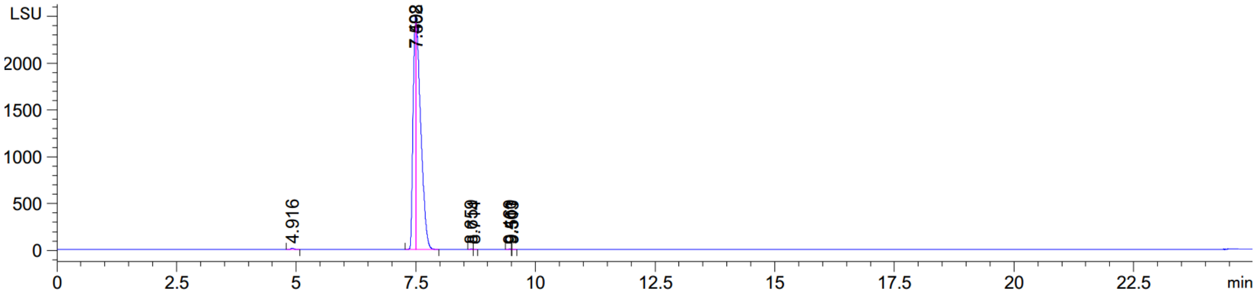

LSU
2000
1500
1000
500
0
4.916
7.508
0
2.5
5
7.5
10
12.5
15
17.5
20
22.5
min

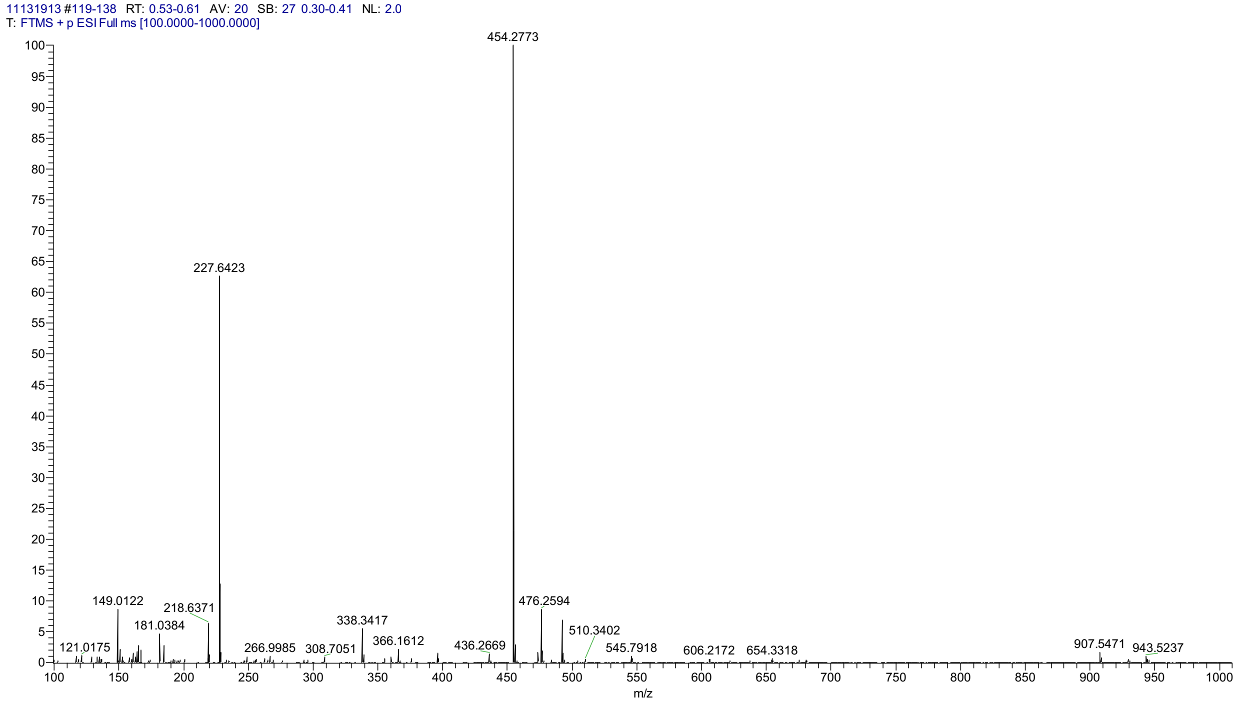

11131913 #119-138 RT: 0.53-0.61 AV: 20 SB: 27 0.30-0.41 NL: 2.0
T: FTMS + p ESI Full ms [100.0000-1000.0000]
454.2773
227.6423
149.0122
218.6371
181.0384
121.0175
266.9985
308.7051
338.3417
366.1612
436.2669
476.2594
510.3402
545.7918
606.2172
654.3318
907.5471
943.5237
m/z

4c(iii)Nne

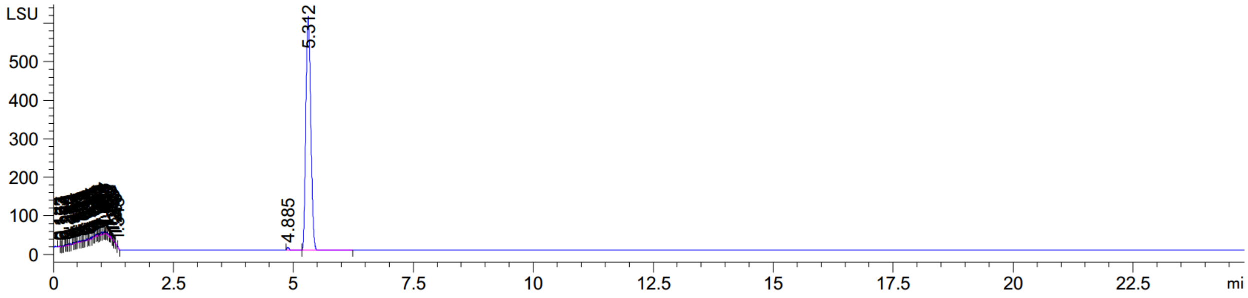
LSU
5.312
4.885
0
2.5
5
7.5
10
12.5
15
17.5
20
22.5
mi

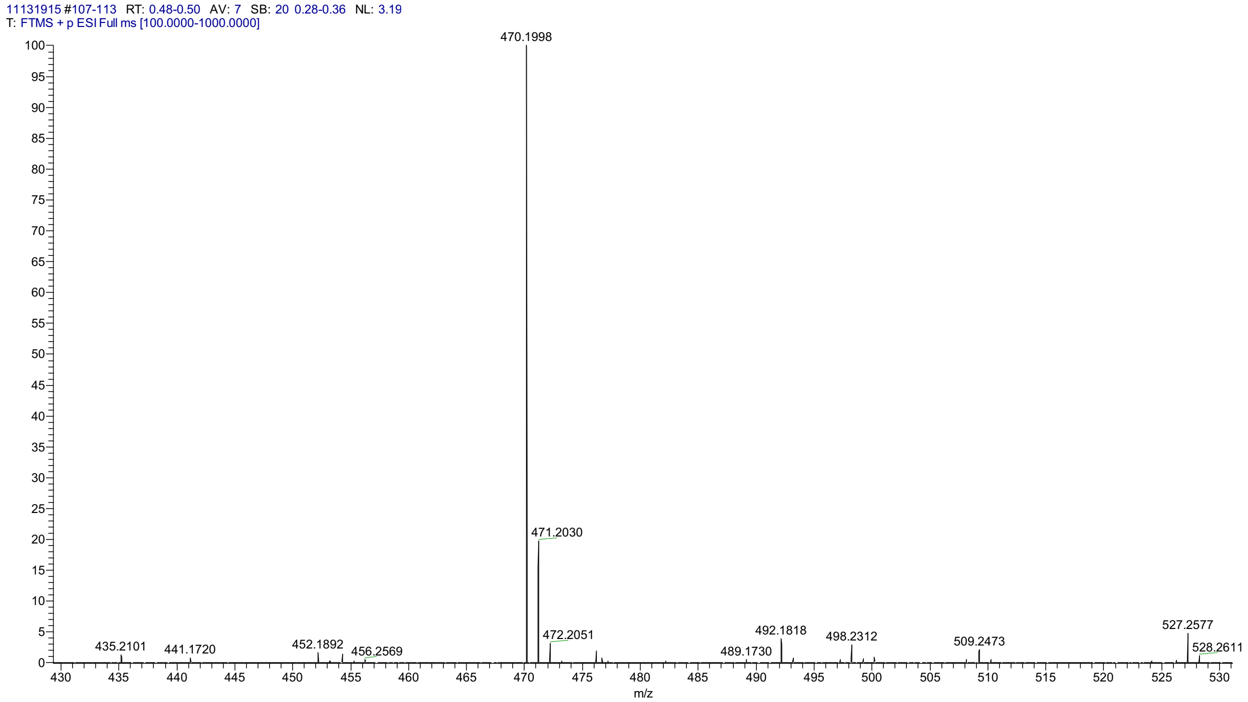
11131915 #107-113 RT: 0.48-0.50 AV: 7 SB: 20 0.28-0.36 NL: 3.19
T: FTMS + p ESI Full ms [100.0000-1000.0000]
470.1998
471.2030
472.2051
435.2101
441.1720
452.1892
456.2569
489.1730
492.1818
498.2312
509.2473
527.2577
528.2611
m/z

4c(iii)ENn

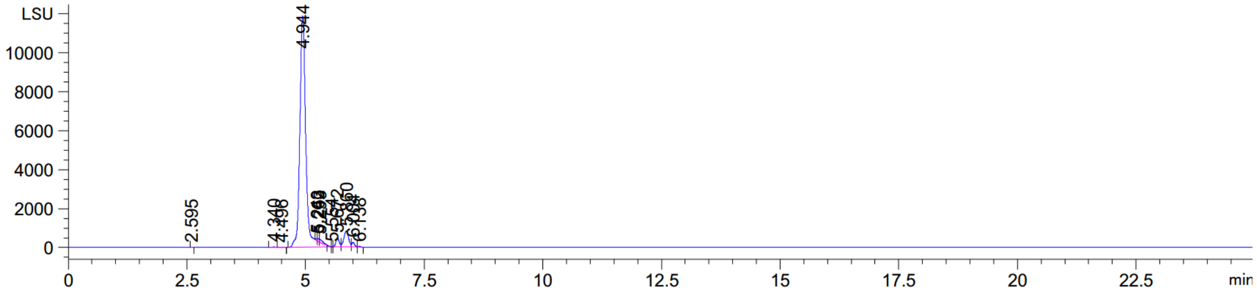
LSU
10000
8000
6000
4000
2000
0
2.595
4.944
0
2.5
5
7.5
10
12.5
15
17.5
20
22.5
min

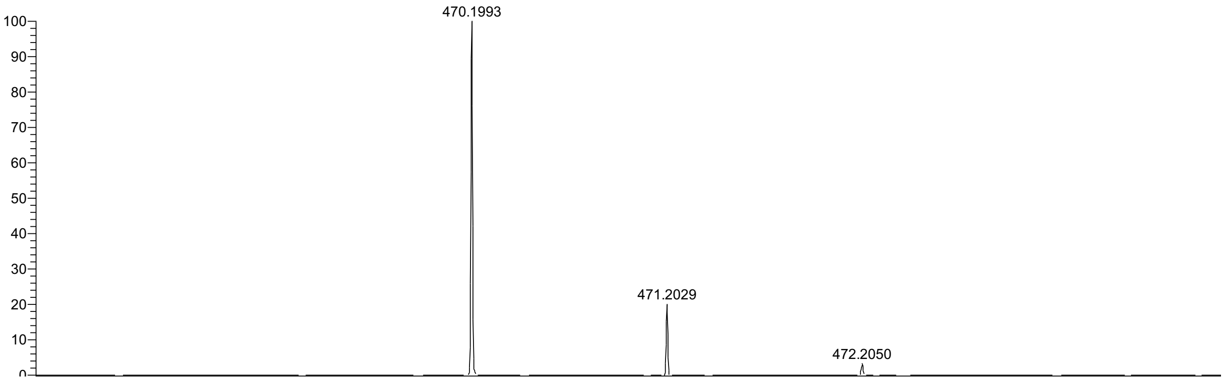
470.1993
471.2029
472.2050
100
90
80
70
60
50
40
30
20
10
0

B. Cell-Based Assays

Cell Lines and Culturing Protocol

HeLa-TrkA cells were cultured in Dulbecco's Modified Eagle's Medium (DMEM) with 10% fetal bovine serum (FBS), 1% penicillin/streptomycin (P/S), and 200 ug/mL Geneticin at 37° C under an atmosphere of 5% CO_2.
HeLa-pcDNA cells were cultured in DMEM with 10% FBS, 1% P/S, and 200 ug/mL Geneticin at 37° C under an atmosphere of 5% CO_2.
HEK293-TrkB cells were cultured in DMEM with 10% FBS, 1% P/S, and 200 ug/mL Geneticin at 37° C under an atmosphere of 5% CO_2.
HEK293 cells were cultured in DMEM with 10% FBS, 1% P/S at 37° C under an atmosphere of 5% CO_2.
NIH3T3-TrkC cells were cultured in DMEM/F12 media with 10% calf serum (CS), 1% P/S, and 200 ug/mL Geneticin at 37° C under an atmosphere of 5% CO_2.
NIH3T3 cells were cultures in DMEM/F12 media with 10% CS and 1% P/S at 37° C under an atmosphere of 5% CO_2.
PC12 cells were cultured in DMEM with 10% horse serum (HS), 5% FBS, 1% P/S at 37° C under an atmosphere of 5% CO_2.

Cytotoxicity Assays

Procedure and Cytotoxicity

Cells were plated in 96-well plates at a density of 2000 cells/well and let adhere for 24 hours. Compounds were added to a maximum concentration of 100 μM to determine if they have any cytotoxic effects. Compounds were incubated with cells for 48-72 hours, after which cell viability was determined via an alamarBlue assay and normalized to cells grown in complete media to 100% survival. Gambogic amide is used as a cytotoxic control. No significant cytotoxic effects were seen by any tested compounds in HeLa-TrkA (Fig S1a), HEK293-TrkB (Fig S1b), or NIH3T3-TrkC (Fig S1c) up to 100 μM.

a

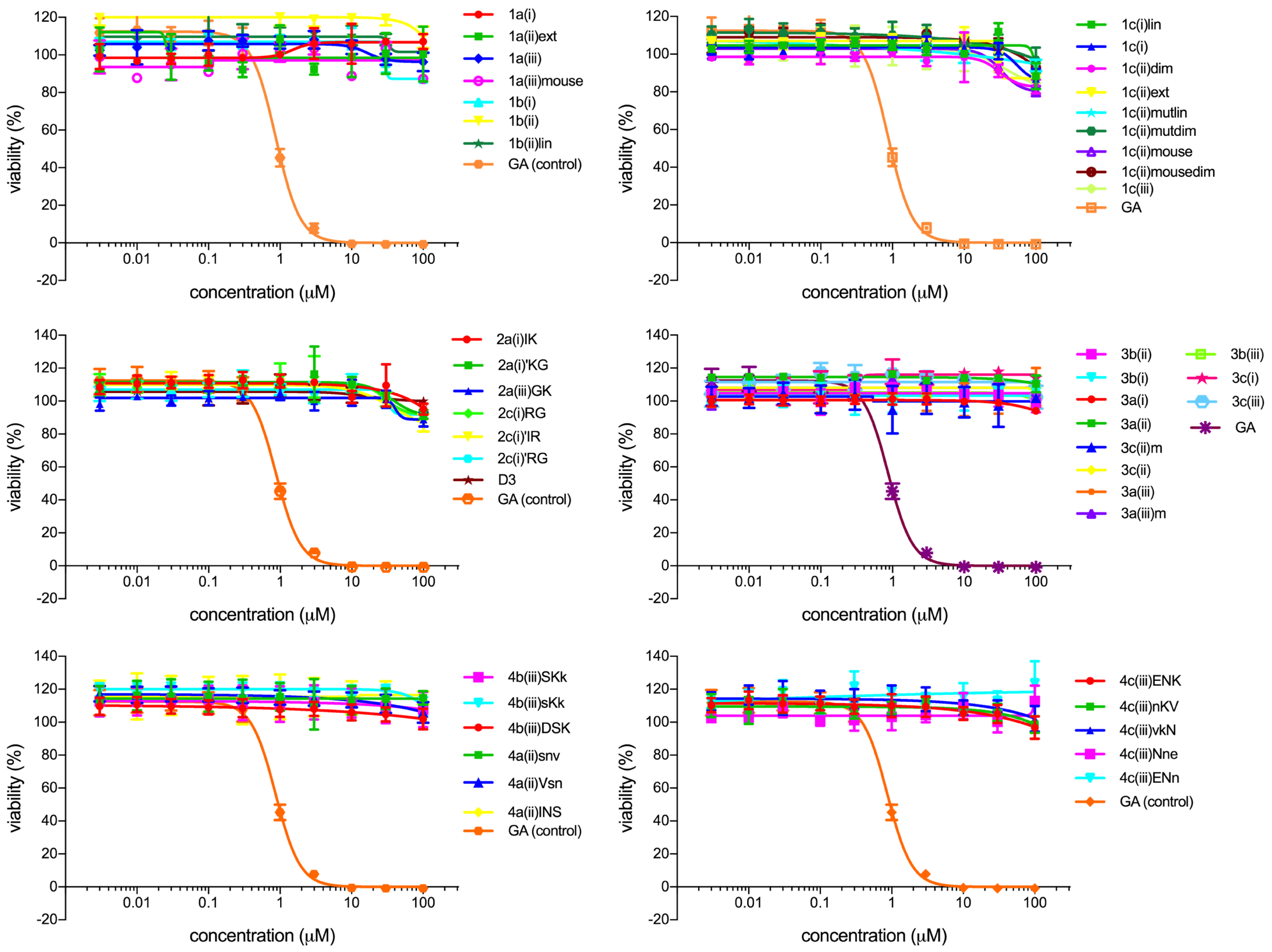

b

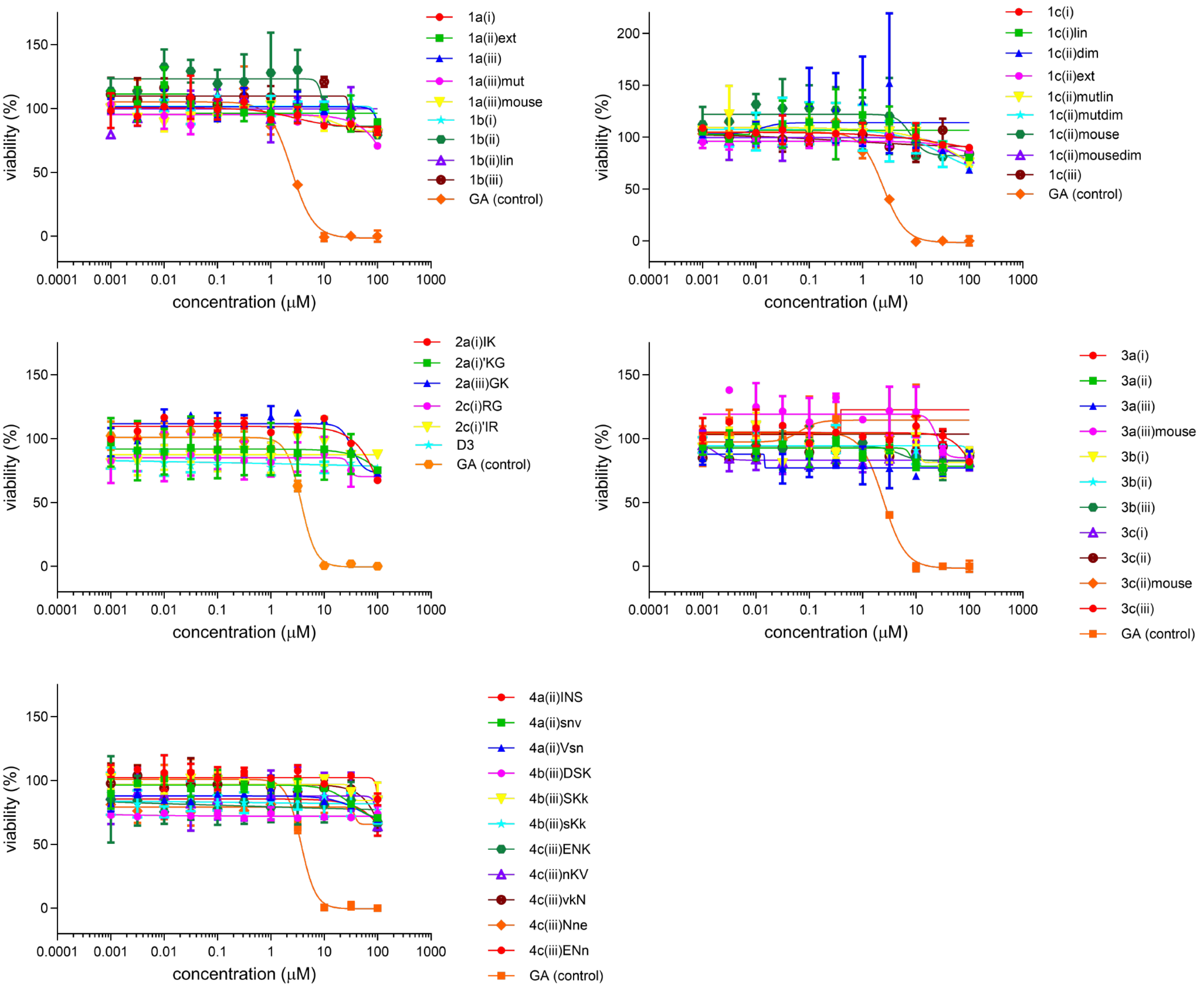
viability (%)
concentration (μM)
1a(i)
1a(ii)ext
1a(iii)
1a(iii)mut
1a(iii)mouse
1b(i)
1b(ii)
1b(ii)lin
1b(iii)
GA (control)
viability (%)
concentration (μM)
1c(i)
1c(i)lin
1c(ii)dim
1c(ii)ext
1c(ii)mutlin
1c(ii)mutdim
1c(ii)mouse
1c(ii)mousedim
1c(iii)
GA (control)
viability (%)
concentration (μM)
2a(i)IK
2a(i)'KG
2a(iii)GK
2c(i)RG
2c(i)'IR
D3
GA (control)
viability (%)
concentration (μM)
3a(i)
3a(ii)
3a(iii)
3a(iii)mouse
3b(i)
3b(ii)
3b(iii)
3c(i)
3c(ii)
3c(ii)mouse
3c(iii)
GA (control)
viability (%)
concentration (μM)
4a(ii)INS
4a(ii)snv
4a(ii)Vsn
4b(iii)DSK
4b(iii)SKk
4b(iii)sKk
4c(iii)ENK
4c(iii)nKV
4c(iii)vkN
4c(iii)Nne
4c(iii)ENn
GA (control)

c

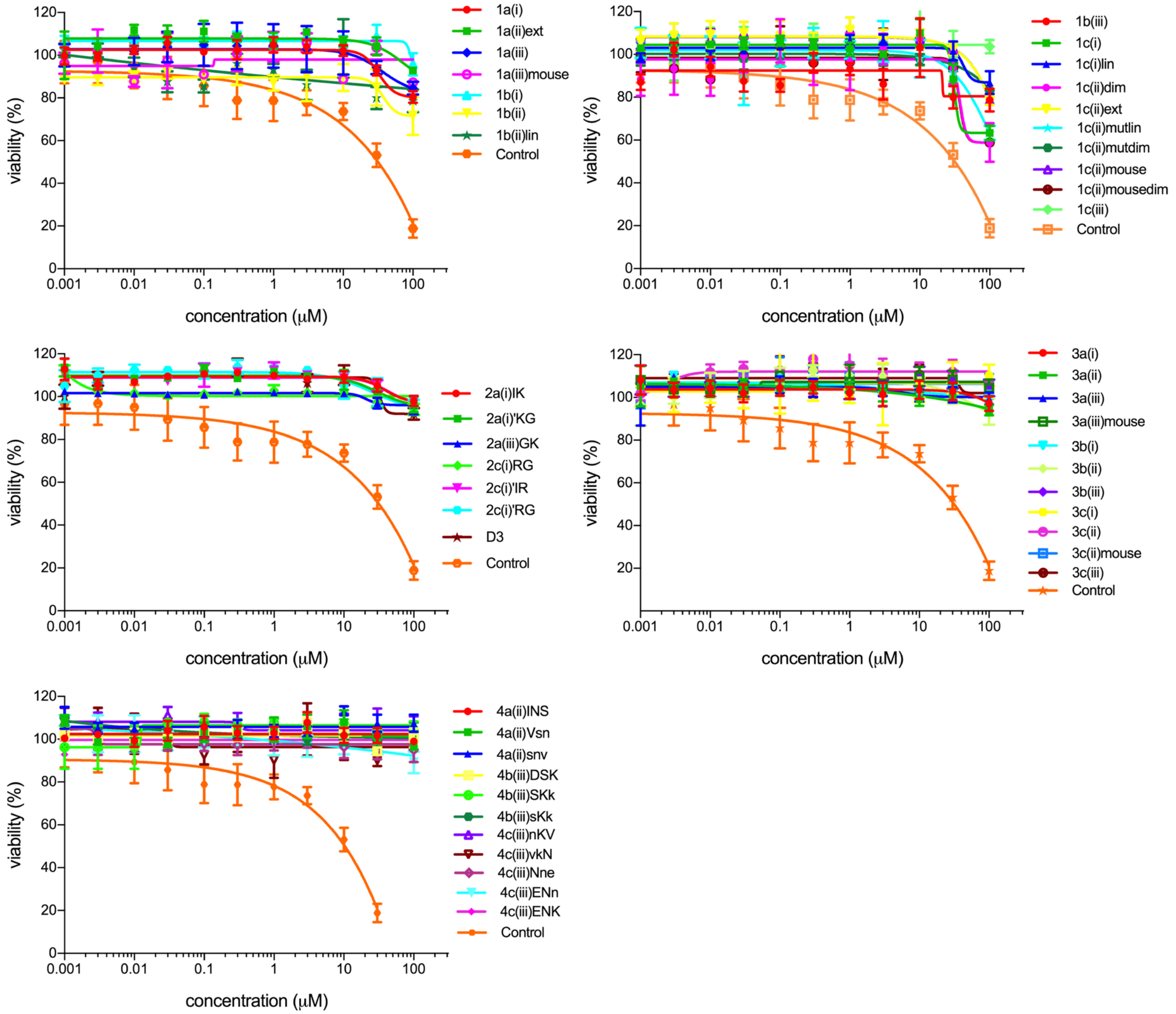

Fig S1 Cytotoxicity of all synthesized compounds in **a** Hela-TrkA, **b** HEK293-TrkB, and **c** NIH3T3-TrkC cell lines.

Cell Survival Compound Screen

General Procedure

Cells were seeded at a density of 2000 cells per well. Cells were incubated in complete media for 24 hours. The media was aspirated, the cells washed twice with Dulbecco's Phosphate Buffered Saline, and the media was replaced with serum-free media to induce apoptosis unless otherwise halted. Compound was added to cells (50 μM compound to HeLa-TrkA and NIH3T3-TrkC cells, 0.4 μM for HEK293-TrkB) alone (agonism) or in the presence of suboptimal neurotrophin (~25 to 30% survival, 0.2 nM NGF, 0.6 nM BDNF, or 0.2 nM NT3 for TrkA, B, or C expressing cells respectively). Cell survival was measured via alamarBlue assay after 48-72 hours to determine cell viability. Data was normalized to DMSO treatment (0%) and optimal neurotrophin (100%, 2.0 nM NGF, 1.0 nM BDNF, 2.0 nM NT-3 for TrkA, B, or C expressing cells, respectively).

Cell Survival of HeLa-TrkA Cells

HeLa-TrkA cells were treated with 50 μM compound with or without suboptimal (0.2 nM) levels of NGF and cell survival was analyzed after 48-72 hours by the alamarBlue assay. Data was normalized to DMSO (0%) and 2.0 nM NGF (100%). Data is represented as the average of 3-6 points +/- standard deviation from the mean.

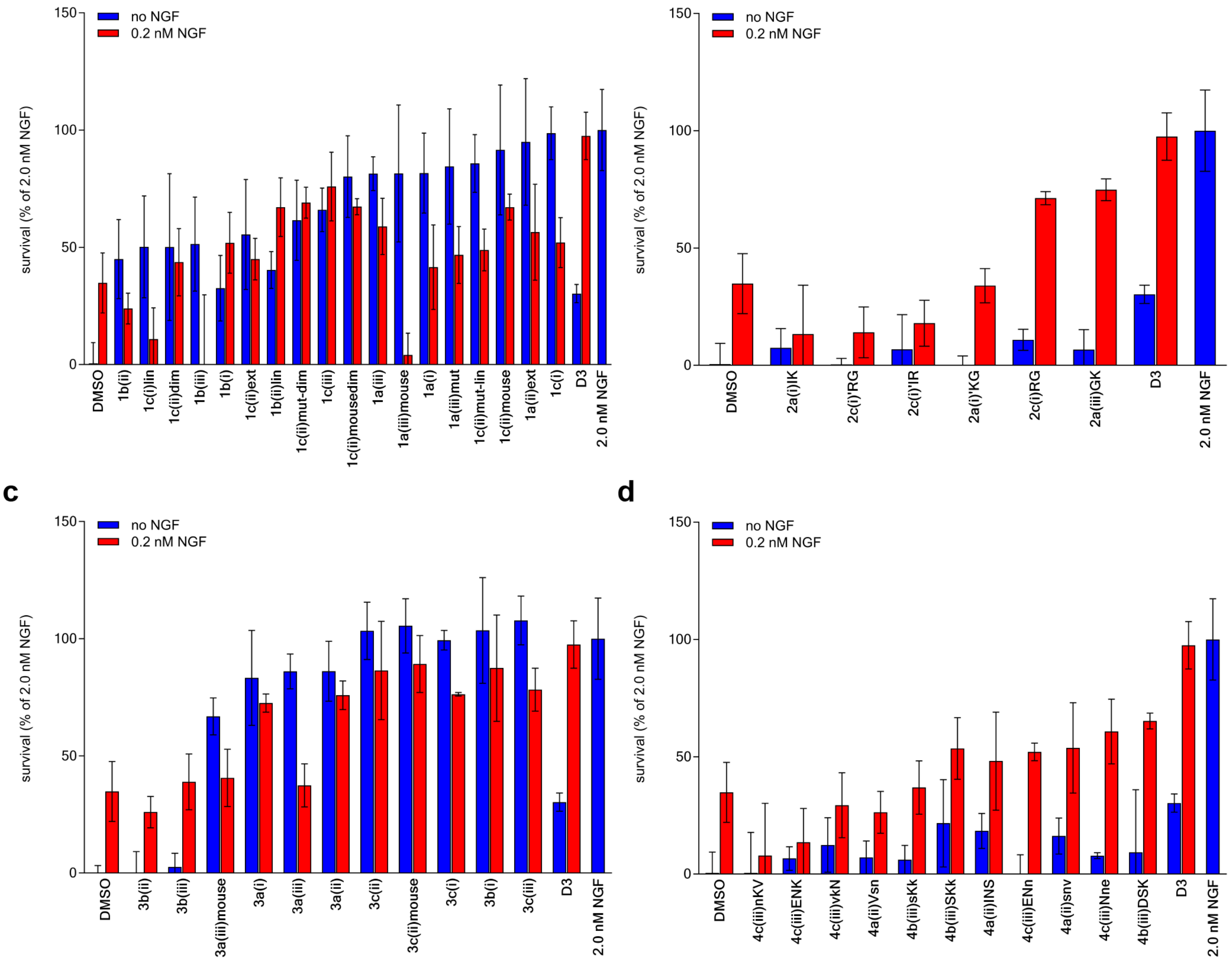

Fig S2 Cell survival of HeLa-TrkA cells with compound series **1-4** (**a-d**, respectively) at 50 μM.

Cell Survival of HEK293-TrkB Cells

HEK293-TrkB cells were treated with 0.4 µM compound with or without suboptimal (0.6 nM) levels of BDNF and cell survival was analyzed after 48-72 hours by the alamarBlue assay. Data was normalized to DMSO (0%) and 1.0 nM BDNF (100%). Data is represented as the average of 3-6 points +/- standard deviation from the mean.

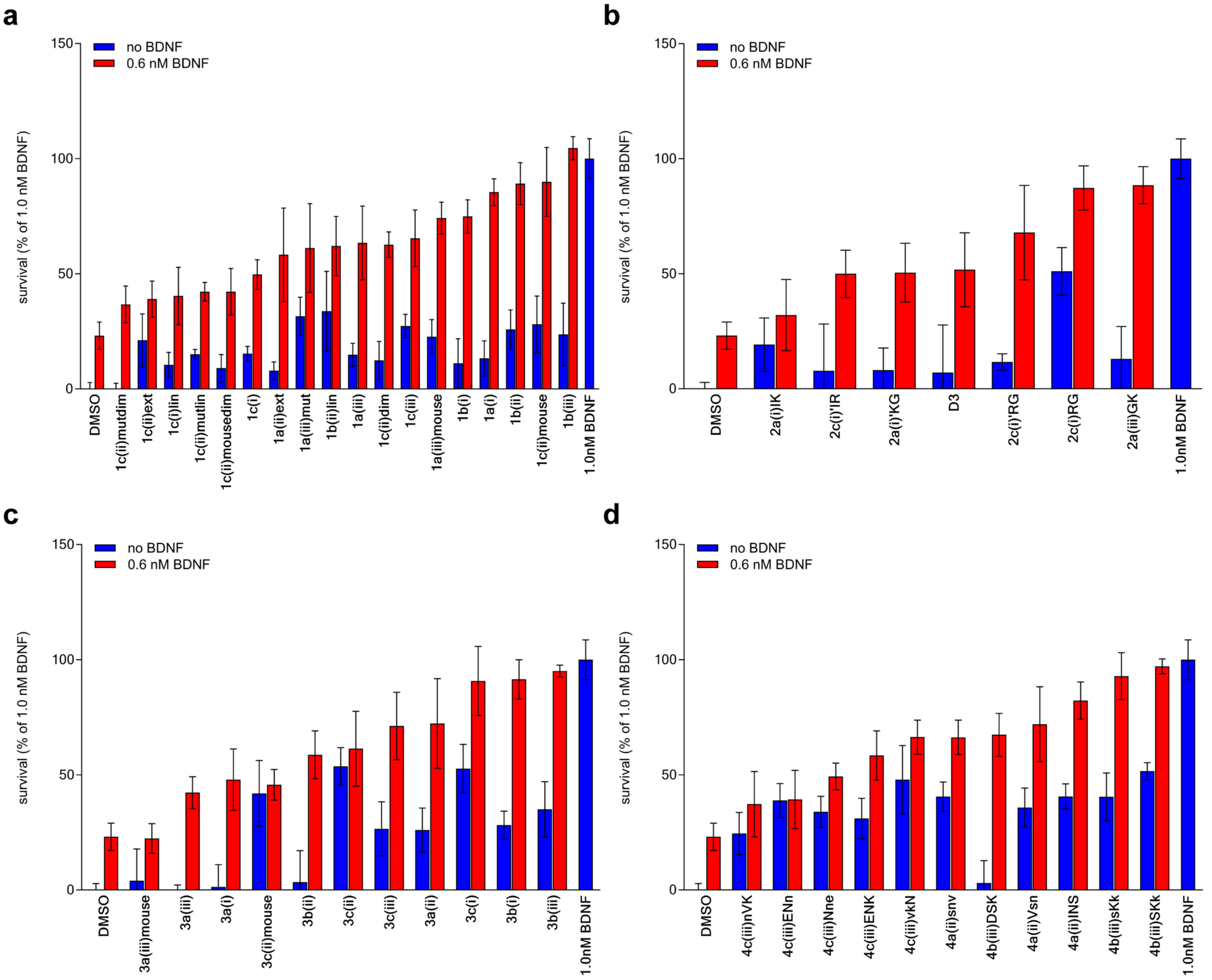

Fig S3 Cell survival of HEK293-TrkB cells with compound series **1-4** (**a-d**, respectively) at 0.4 µM.

Cell Survival of NIH3T3-TrkC Cells

NIH3T3-TrkC cells were treated with 50 μM compound with or without suboptimal (0.2 nM) levels of NT-3 and cell survival was analyzed after 48-72 hours by the alamarBlue assay. Data was normalized to DMSO (0%) and 2.0 nM NT-3 (100%). Data is represented as the average of 3-6 points +/- standard deviation from the mean.

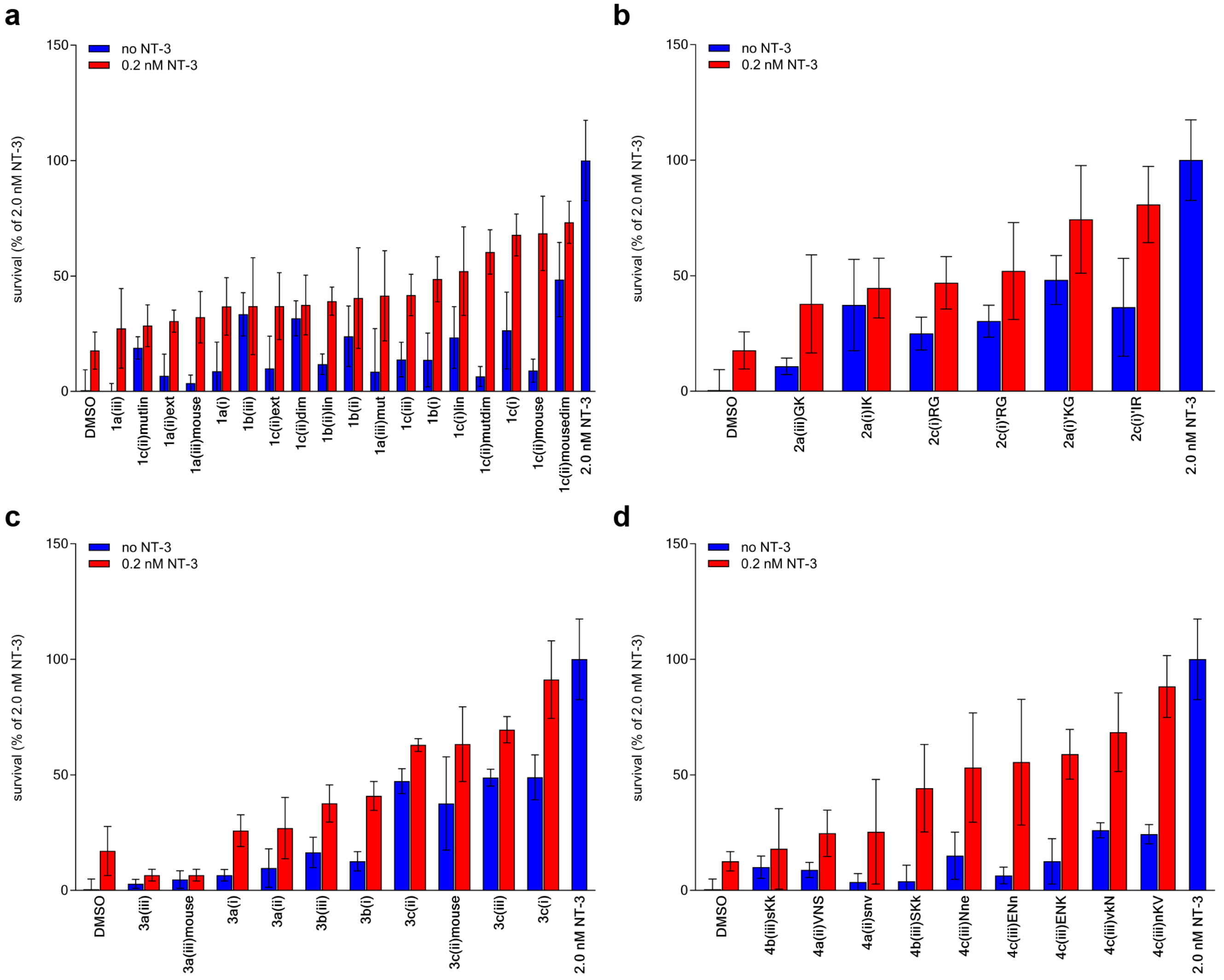

Fig S4 Cell survival of NIH3T3-TrkC cells with compound series **1-4** (**a-d**, respectively) at 50 μM

Cell Survival Dose Response

General Procedure

The most promising compounds from the screen were selected for each cell line. Cells were treated with a serial dilution of the compound in serum free media with or without suboptimal neurotrophin and incubated for 48-72 hours, after which an alamarBlue assay was done to determine cell viability. Cell viability was normalized to DMSO (0%) and optimal neurotrophin (100%), depending on cell type. EC_{50} was calculated in GraphPad Prism 10.2 using the dose response “[agonist] vs response – Variable slope (four parameters)” function. Data is presented as the mean +/- standard deviation of an experiment with 3-6 replicates.

Enzyme-Linked Fixed-Cell Immunoassay (ELFI)

General Procedure

Assays were conducted as previously reported.[7] Trk-positive cells (PC12, HEK293-TrkB, and NIH3T3-TrkC) were seeded at a density of $2x10^4$ cells per well in poly-D-lysine (PDL)-coated 96-well white flat-bottomed plates and allowed to adhere for 24 h. Cells were washed once with SFM, then incubated for 1-2 h in SFM. Serial dilution of compound and/or NT was added and incubated for 15 min (agonism experiments) or 1 h (antagonism). Cells were then washed with DPBS, then fixed for 20 min with buffered 4% formaldehyde solution. Cells were washed and permeabilized 6 times with washing buffer (WB; 0.01 M PBS, 0.05%/v Tween-20, pH 7.4), then blocked for 1 h with blocking buffer (BB; 0.01 M PBS, 0.05%/v Tween-20, 10% BSA, pH 7.4). Cells were incubated with primary antibody (anti-pAkt {Phospho-Akt (Ser473) (D9E) XP® Rabbit mAb #4060, Cell Signaling Technology} 1:200 dilution or anti-pMAPK {Phospho-p44/42 MAPK (Erk1/2) (Thr202/Tyr204) (D13.14.4E) XP® Rabbit mAb #4370, Cell Signaling Technology} 1:100 dilution) diluted in WB + 0.1% BSA for 4 hours at room temperature, then washed 6x with WB, followed by incubation for 1 h with 1:1000 dilution of 2° antibody-HRP conjugate (Anti-rabbit IgG, HRP-linked Antibody #7074, Cell Signaling Technology). Cells were washed 6x with WB, then levels of phosphorylation quantified using a SuperSignal ELISA Pico Chemiluminescent Substrate (Thermo Scientific). Data is normalized to DMSO (0%) and maximum signal imparted by neurotrophin (10 ng/mL NT, 100%). The antibodies are stripped by treating with stripping buffer (SB; 6M guanidine-HCl, 0.2%/v Triton X-100, 20mM tris-HCl, pH 7.5) for 5 min, followed by 6 washed with WB. The process is then repeated on the same cells using the other primary antibody. Statistical analyses are carried out using two-way ANOVA followed by Dunnett’s t-test in GraphPad Prism 10.2.

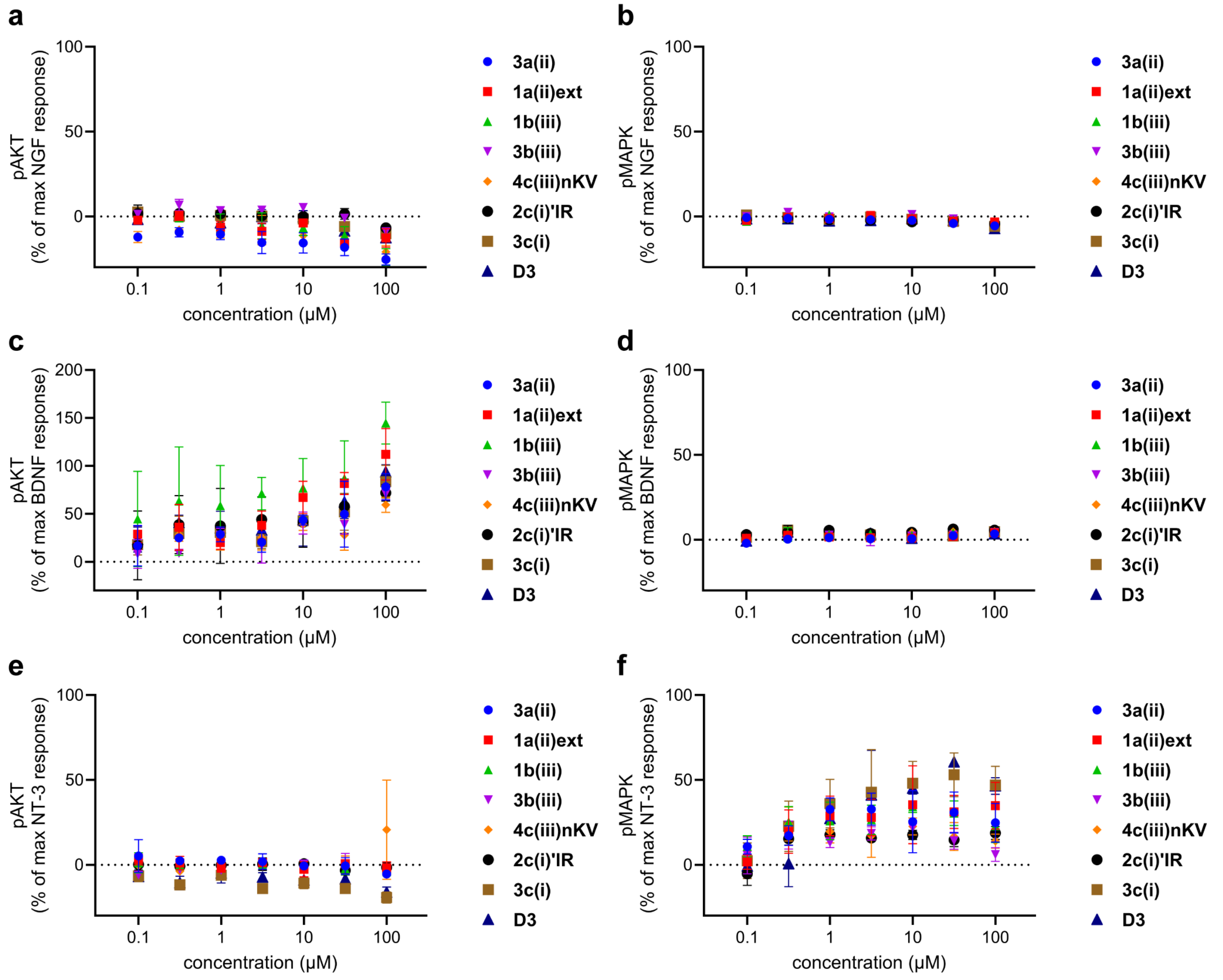

Fig S5 Enzyme-linked fixed-cell immunoassay on TrkA (**a, b**), TrkB (**c, d**), or TrkC (**e, f**)-expressing cells with no parent neurotrophin to test for compound agonism.

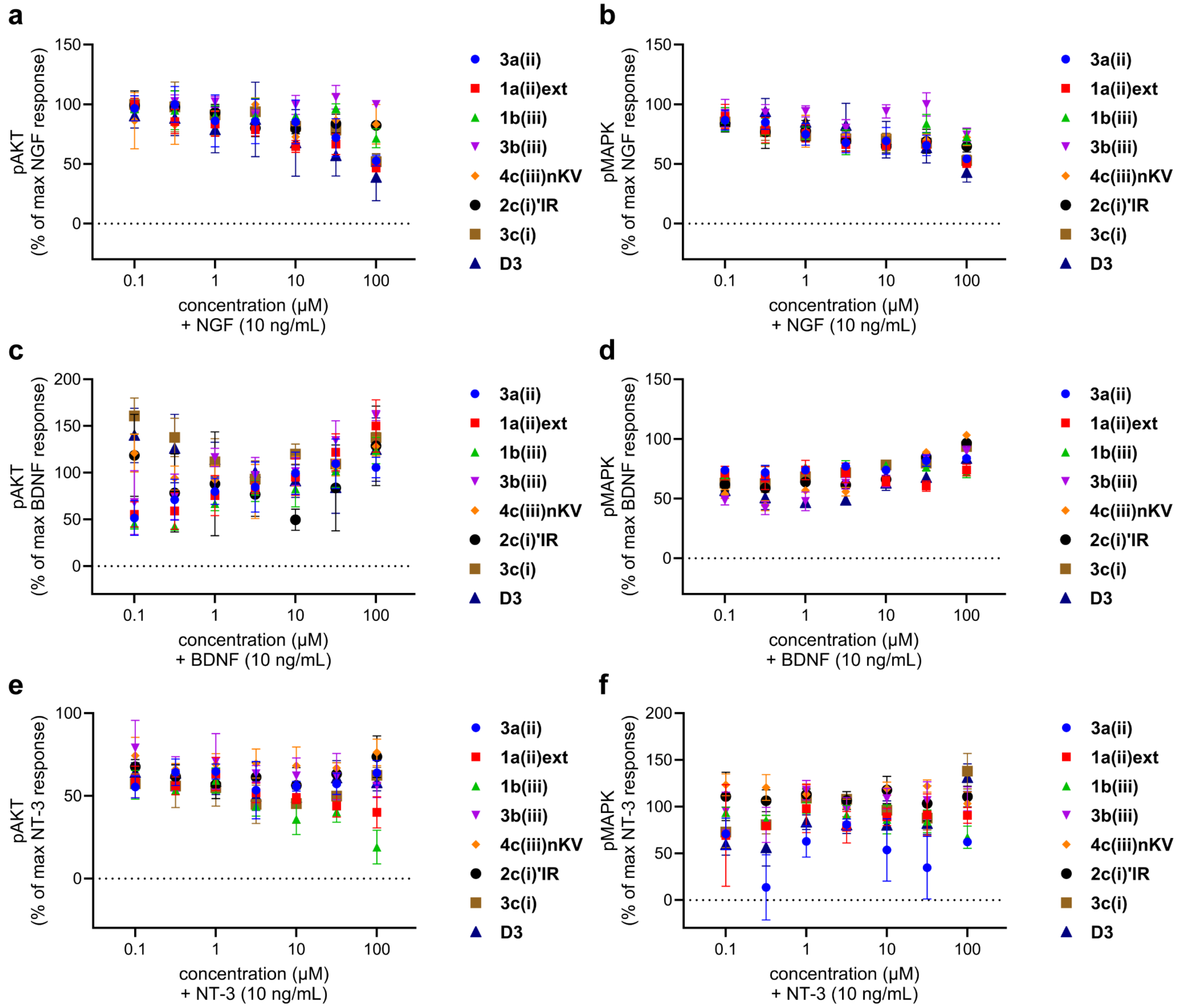

Fig S6 Enzyme-linked fixed-cell immunoassay on TrkA (**a, b**), TrkB (**c, d**), or TrkC (**e, f**)-expressing cells with optimal levels of parent neurotrophin to test for antagonism.

C. References

(1) Burgess, K.; Jacutin, S. E.; Lim, D.; Shitangkoon, A. An Approach to Photolabile, Fluorescent Protecting Groups. *The Journal of Organic Chemistry* **1997**, *62* (15), 5165-5168. DOI: 10.1021/jo9702608.

(2) Lundquist; Pelletier, J. C. Improved Solid-Phase Peptide Synthesis Method Utilizing α-Azide-Protected Amino Acids. *Organic Letters* **2001**, *3* (5), 781-783. DOI: 10.1021/ol0155485.

(3) Turner, R. A.; Oliver, A. G.; Lokey, R. S. Click Chemistry as a Macrocyclization Tool in the Solid-Phase Synthesis of Small Cyclic Peptides. *Organic Letters* **2007**, *9* (24), 5011-5014. DOI: 10.1021/ol702228u.

(4) Zaccaro, M. C.; Lee, H. B.; Pattarawarapan, M.; Xia, Z.; Caron, A.; L'Heureux, P.-J.; Bengio, Y.; Burgess, K.; Saragovi, H. U. Selective Small Molecule Peptidomimetic Ligands of TrkC and TrkA Receptors Afford Discrete or Complete Neurotrophic Activities. *Chemistry & Biology* **2005**, *12* (9), 1015-1028. DOI: https://doi.org/10.1016/j.chembiol.2005.06.015.

(5) Battistini, L.; Burreddu, P.; Carta, P.; Rassu, G.; Auzzas, L.; Curti, C.; Zanardi, F.; Manzoni, L.; Araldi, E. M. V.; Scolastico, C.; Casiraghi, G. 4-Aminoproline-based arginine-glycine-aspartate integrin binders with exposed ligation points: practical in-solution synthesis, conjugation and binding affinity evaluation. *Organic & Biomolecular Chemistry* **2009**, *7* (23), 4924-4935, 10.1039/B914836A. DOI: 10.1039/B914836A.

(6) Zanardi, F.; Burreddu, P.; Rassu, G.; Auzzas, L.; Battistini, L.; Curti, C.; Sartori, A.; Nicastro, G.; Menchi, G.; Cini, N.; et al. Discovery of Subnanomolar Arginine-Glycine-Aspartate-Based αVβ3/αVβ5 Integrin Binders Embedding 4-Aminoproline Residues. *Journal of Medicinal Chemistry* **2008**, *51* (6), 1771-1782. DOI: 10.1021/jm701214z.

(7) Boltaev, U.; Meyer, Y.; Tolibzoda, F.; Jacques, T.; Gassaway, M.; Xu, Q.; Wagner, F.; Zhang, Y.-L.; Palmer, M.; Holson, E.; Sames, D. Multiplex quantitative assays indicate a need for reevaluating reported small-molecule TrkB agonists. *Science Signaling* **2017**, *10* (493), eaal1670. DOI: 10.1126/scisignal.aal1670 (acccessed 2024/03/29).
